# Horizontal DNA Transfer Mechanisms of Bacteria as Weapons of Intragenomic Conflict

**DOI:** 10.1371/journal.pbio.1002394

**Published:** 2016-03-02

**Authors:** Nicholas J. Croucher, Rafal Mostowy, Christopher Wymant, Paul Turner, Stephen D. Bentley, Christophe Fraser

**Affiliations:** 1 Department of Infectious Disease Epidemiology, Imperial College London, London, United Kingdom; 2 Cambodia Oxford Medical Research Unit, Angkor Hospital for Children, Siem Reap, Cambodia; 3 Centre for Tropical Medicine and Global Health, Nuffield Department of Medicine, University of Oxford, Oxford, United Kingdom; 4 Pathogen Genomics, Wellcome Trust Sanger Institute, Wellcome Trust Genome Campus, Hinxton, Cambridge, United Kingdom; Institute of Science and Technology Austria (IST Austria), AUSTRIA

## Abstract

Horizontal DNA transfer (HDT) is a pervasive mechanism of diversification in many microbial species, but its primary evolutionary role remains controversial. Much recent research has emphasised the adaptive benefit of acquiring novel DNA, but here we argue instead that intragenomic conflict provides a coherent framework for understanding the evolutionary origins of HDT. To test this hypothesis, we developed a mathematical model of a clonally descended bacterial population undergoing HDT through transmission of mobile genetic elements (MGEs) and genetic transformation. Including the known bias of transformation toward the acquisition of shorter alleles into the model suggested it could be an effective means of counteracting the spread of MGEs. Both constitutive and transient competence for transformation were found to provide an effective defence against parasitic MGEs; transient competence could also be effective at permitting the selective spread of MGEs conferring a benefit on their host bacterium. The coordination of transient competence with cell–cell killing, observed in multiple species, was found to result in synergistic blocking of MGE transmission through releasing genomic DNA for homologous recombination while simultaneously reducing horizontal MGE spread by lowering the local cell density. To evaluate the feasibility of the functions suggested by the modelling analysis, we analysed genomic data from longitudinal sampling of individuals carrying *Streptococcus pneumoniae*. This revealed the frequent within-host coexistence of clonally descended cells that differed in their MGE infection status, a necessary condition for the proposed mechanism to operate. Additionally, we found multiple examples of MGEs inhibiting transformation through integrative disruption of genes encoding the competence machinery across many species, providing evidence of an ongoing “arms race.” Reduced rates of transformation have also been observed in cells infected by MGEs that reduce the concentration of extracellular DNA through secretion of DNases. Simulations predicted that either mechanism of limiting transformation would benefit individual MGEs, but also that this tactic’s effectiveness was limited by competition with other MGEs coinfecting the same cell. A further observed behaviour we hypothesised to reduce elimination by transformation was MGE activation when cells become competent. Our model predicted that this response was effective at counteracting transformation independently of competing MGEs. Therefore, this framework is able to explain both common properties of MGEs, and the seemingly paradoxical bacterial behaviours of transformation and cell–cell killing within clonally related populations, as the consequences of intragenomic conflict between self-replicating chromosomes and parasitic MGEs. The antagonistic nature of the different mechanisms of HDT over short timescales means their contribution to bacterial evolution is likely to be substantially greater than previously appreciated.

## Abstract 2

Modelling and high-resolution genomic data demonstrate the evolutionary significance of an ongoing arms race between bacterial uptake of environmental DNA ("transformation") and horizontal DNA transfer driven by mobile genetic elements.

## Author Summary

Bacteria are able to rapidly change their characteristics, such as antibiotic resistance, by acquiring genes from surrounding cells. Some sets of genes, called mobile genetic elements (MGEs), drive their own movement between bacterial cells by transmitting in viral or viral-like manners. These selfish genes spread themselves, even if harmful to their bacterial host. Some bacteria also actively absorb DNA from the environment, a process known as transformation. Why bacteria employ transformation is controversial. Importing all DNA molecules at the same rate means cells acquire detrimental and beneficial mutations at equal frequencies. However, transformation does exhibit a known bias: it preferentially imports shorter DNA molecules, meaning it tends to delete, rather than insert, genes. By incorporating this bias into computer simulations, we show that the spread of a selfish mobile genetic element between bacteria is inhibited when cells use transformation to delete it from their genomes. We hypothesised that this ability to delete selfish mobile genetic elements is an important function of transformation. To test this hypothesis, we analysed longitudinal bacterial samples from individuals colonised by *S*. *pneumoniae* and found the bacterial cells often differed in the mobile genes they contained while still in the same host and are, therefore, able to exchange DNA by transformation. Additionally, transformable bacteria had fewer selfish mobile genes than related nontransformable bacteria. We also identified examples of mobile elements that employ various tactics to prevent their host cells undergoing transformation, indicating that some selfish mobile genes are able to circumvent deletion-by-transformation. Hence, we conclude bacterial evolution is strongly influenced by an ongoing arms race between cells and selfish mobile genes.

Horizontal exchange of DNA is common in many bacterial species [1,2], and it has become clinically relevant in recent decades through facilitating the spread of antimicrobial resistance [3] and evasion of vaccine-induced immunity [4]. However, the horizontal movement of sequence between cells is an ancient process, as revealed by its substantial effects on the overall tree of life [5]. This is despite the many risks to a recipient cell from the acquisition of DNA from an external source, such as the replicative, transcriptional, and metabolic burden of new genes, as well as the possible disruption of regulatory and protein interaction networks [6]. Perhaps most importantly, there is the potential for the acquisition of genomic parasites, against which all genomes of self-replicating cells must defend themselves [7].

Horizontal DNA transfer (HDT), a term we define to encompass all movement of heritable genetic material whether or not it alters the recipient genome, is frequently driven by such parasitic loci, grouped together as mobile genetic elements (MGEs). MGEs encode at least some of the machinery required for their transfer between cells (horizontal transmission), and consequently drive two of the three principal mechanisms of HDT in bacteria: conjugation, the movement of DNA through an MGE-encoded conjugative pilus [8]; and transduction, the movement of DNA through an MGE-encoded virion particle [9]. To defend themselves, cells encode means of inhibiting this lateral spread, such as restriction-modification and clustered, regularly interspaced, short palindromic repeats (CRISPR) systems [10,11]. However, many MGEs insert into their host cell’s chromosome, and post-integration, these defences cannot prevent such genomic parasites from subsequently maintaining a stable association with their host and passing into descendants (vertical transmission).

The third principal mechanism of HDT is transformation, the import of exogenous DNA that can be incorporated into the genome through homologous recombination, first identified in *S*. *pneumoniae* [12,13]. Natural genetic transformation is not driven by MGEs, but instead facilitated by competence machinery encoded by the bacterium itself; while not ubiquitous across bacteria, the competence machinery is usually conserved across a species [14,15]. Typically, the first step of transformation involves binding of double-stranded DNA (dsDNA) to a surface receptor (ComEA in gram-positive bacteria; ComE in gram-negative bacteria). This requires a pseudopilus formed of ComY or ComG proteins (in gram-positive bacteria) or Pil proteins (in gram-negative bacteria) [14,16,17]. The bound DNA then passes through a specialised pore (ComEC in gram-positive bacteria; ComA in gram-negative bacteria) [18] that translocates the nucleic acid into the cytosol in a single stranded form, with the concomitant degradation of the complementary strand [19,20]. In gram-positive bacteria, ComFA appears to have a role in driving this DNA import [21]; in the gram-negative bacterium *Haemophilus influenzae*, ComM has been identified as playing a role in transformation post-import [22]. Upon import, this single-stranded DNA (ssDNA) is itself cut into fragments [23,24], with a median length of approximately 6.6 kb in *S*. *pneumoniae* [23]. These ssDNA fragments are then bound by proteins inside the cell, culminating in the formation of a RecA nucleoprotein filament [25,26]. This is the form in which the ssDNA can invade the host chromosome duplex, potentially resulting in homologous recombination [27]. The components of this highly-specialised competence machinery are encoded by multiple nonmobile loci within the cellular genome.

With the apparent exceptions of *Helicobacter pylori* and the Neisseriaceae, naturally transformable bacteria tend to tightly control expression of the competence machinery [15], making it difficult to identify the full range of species in which the system is active [28,29]. Distinct quorum-sensing systems based on secreted peptide pheromones are used in *S*. *pneumoniae* [30], *Bacillus subtilis* [31] and *Streptococcus thermophilus* [32], whereas nonpeptide autoinducers regulate expression in *Vibrio cholerae* [33]. These signals are then transduced to a regulator of transcription, such as the competence-specific sigma factor σ^X^ in *S*. *pneumoniae* [34] or the ComK transcriptional regulator in *B*. *subtilis* [35]. In addition to the genes directly required for competence, these often activate a range of other coordinated activities as part of what appears to be a broader stress response, referred to by terms such as ‘K state’ or ‘X state’ [15]. Notably, in the well-studied species *S*. *pneumoniae* and *B*. *subtilis*, competence has been found to be coordinated with the secretion of bacteriocins that kill noncompetent cells in a process termed “fratricide” or “cannibalism” [36], as well as cell cycle arrest [37,38]. In *B*. *subtilis*, the noisy transcription of *comK* results in phenotypic differentiation termed “bet hedging” [39,40], with the population reaching a dynamic equilibrium in which a subset of the population actively replicates, while around 15% of the population are competent, nonreplicative, persister cells [41,42]. Hence, competence is expressed in a diverse set of patterns in different species.

What selective advantage transformation provides to the cell remains controversial [15,43]. Three primary groups of hypotheses have been proposed as explanations. The first is that HDT facilitates the acquisition of beneficial genetic polymorphisms and, therefore, can be considered analogous to eukaryotic sexual reproduction. Straightforward forms of these models can demonstrate that recombining populations often have a higher fitness than nonrecombining populations [44–46], but such explanations are disfavoured, as they rely upon group selection [47]. Identifying an advantage at the level of the individual is difficult, as the competence system facilitates the acquisition of beneficial and deleterious sequence at the same rate in these models. One explanation is the existence of synergistic epistasis, the situation in which polymorphisms interact such that their cumulative effect on an individual’s fitness is greater than the sum of their individual effects [48–50]; however, experimental investigation of adaptive changes from in vitro evolution experiments has found antagonistic epistasis between mutations to be more common [51,52]. Another set of models uses the scenario of recombining cells entering a new niche [53], such that acquired alleles are biased toward being beneficial, as they arise from donors already better adapted to the new conditions. Transformation can, therefore, be advantageous if competent cells encounter different environments continuously [46] or periodically [41], akin to the “Red Queen” hypothesis that individuals must unceasingly change to avoid decreasing in fitness [54]. However, exchanges of DNA between different lineages of transformable bacteria are not frequent enough to disrupt predominately clonal population structures [55–57], with years often elapsing between substantial imports of divergent sequence [58].

The infrequent exchange of DNA between lineages does not preclude exchange of sequence between closely related genotypes facilitating the repair of deleterious mutations. Owing to the physical structuring of bacterial populations, it is much more likely that cells will undergo recombination with clonally related neighbours than with other, divergent lineages. Nevertheless, such a mechanism seems unlikely to be the primary purpose of the competence machinery. Transformation cannot distinguish recent point mutations from the alleles that repair such spontaneous changes; furthermore, previous models have identified the problem that if deleterious mutations are frequent and often result in cell lysis, then the pool of DNA available for transformation will be enriched for lower fitness alleles [48]. Additionally, transformation events that reverse the most commonly observed mutations in *S*. *pneumoniae* are efficiently inhibited by the mismatch repair system [59,60]. Effective against particular base substitutions but not large insertions or deletions, this repair system also has lower, but detectable, activity in *B*. *subtilis* [61,62] and *H*. *influenzae* [63] and is present in many transformable species [64].

The second set of hypotheses suggest that imported DNA is used as a template to repair dsDNA breaks [65]. Potential mutagens have been found to increase the rate of transformation in *S*. *pneumoniae* [66], *Legionella pneumophila* [67], and *H*. *pylori* [68], with conflicting data as to whether the same regulation is observed in *B*. *subtilis* [69–72]. However, experimental work detected no such regulation in *S*. *thermophilus* [73] or *H*. *influenzae* [71,74]. Similarly, there is some evidence for transformation increasing resistance to ultraviolet exposure from experimental work on *B*. *subtilis* [70], but the same result was not observed in *H*. *influenzae* [74,75] or *L*. *pneumophila* [67]. While all bacteria suffer dsDNA breaks as part of normal DNA replication, transformation is found in distantly related species that are unlikely to be subject to particularly high or variable burdens of DNA damage, such as the nasopharyngeal commensals *S*. *pneumoniae*, *H*. *influenzae*, and *Neisseria meningitidis*. Correspondingly, the SOS response of *H*. *influenzae* lacks the translesion repair system [76], important in repair of mutagen-induced damage in *B*. *subtilis* and many other species, while both *N*. *meningitidis* and *S*. *pneumoniae* generally lack identifiable SOS responses [64,77] despite the import of translesion repair genes into at least one *S*. *pneumoniae* isolate on a conjugative element [78]. Hence, there is no strong evidence that the competence system functions as an alternative to, or an enhancement of, the known role of the SOS response in ameliorating the effects of mutagens, although comparisons between the gene content of species in different niches are confounded by the associated variation in genome size [79].

The third set of hypotheses are based on the competence system functioning as a means of scavenging nucleotides from the environment [43,80]. This is consistent with only the minority of transforming DNA being integrated into the chromosome [19], as well as competence being induced by purine starvation in *H*. *influenzae* [81] and by nucleoside starvation in *V*. *cholerae* [82]. Although not known to be naturally transformable itself, orthologues of competence genes in *Escherichia coli* have been found to facilitate nutrient acquisition in vitro [83]. However, some species only develop competence in the absence of nutrient starvation, and others limit the molecules they import in a sequence-specific manner to avoid acquiring DNA from other species [15]. Additionally, it is difficult to understand how the import of a single DNA strand as a protected nucleoprotein filament, with the other strand degraded extracellularly, maximizes the acquisition of nutrients, whereas it is optimised for integration of imported DNA into the chromosome [41].

Here, we present a novel hypothesis motivated by several key characteristics of HDT by transformation. The first is that physical structuring of populations means most exchanges are between isogenic, or near-isogenic, cells. The second is that in vitro and in vivo characterisation of recombinant *S*. *pneumoniae* [4,60,84] and *H*. *influenzae* [85] isolates has demonstrated that transformation results in the integration of DNA tracts with an approximately geometric length distribution. The majority of such recombinations have been shown to be shorter than common MGEs [60,85,86], which are typically several kilobases in length and often much larger [87]. The third is that homologous recombination is able to span regions of dissimilar sequence in either imported or chromosomal DNA, meaning transformation can alter genome content [88,89]. As the acquisition of longer DNA molecules is limited by extracellular degradation, cleavage on import [23,24], and restriction endonuclease cleavage of novel sequence [89], transformation has a tendency to delete, rather than import, genes. Hence, if infected and uninfected cells exist in close proximity, transformation should act to remove MGEs far more efficiently than it spreads them, making it an effective means of inhibiting the vertical transmission of genomic parasites.

## Results

### Asymmetrical Transfer Benefits Transformable Cells

**Fig 1:**
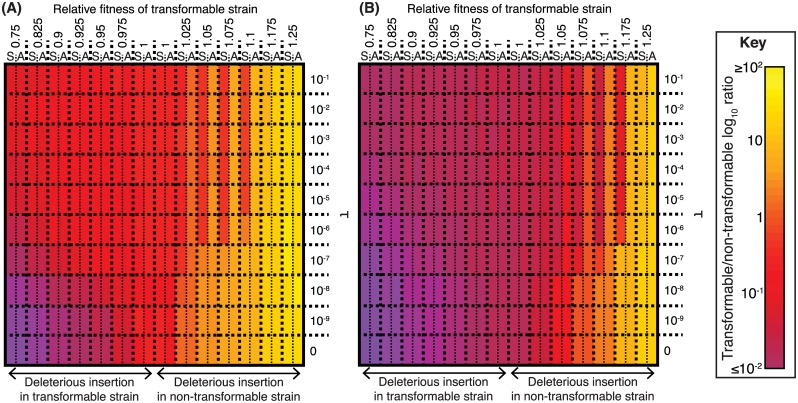
Variation in the effectiveness of transformation when importing DNA from a different strain. (**A**) Heatmap showing the outcome of simulated competitions between two strains, of which only one is transformable. Each cell in the grid represents a specific transformation rate (τ) and relative fitness of the allele at an exchangeable locus within the transformable strain, displayed at the top of each column. Relative fitnesses greater than one indicate the transformable strain has an initial advantage over the nontransformable strain; relative fitnesses below one indicate the nontransformable strain has the initial fitness advantage. Each cell is split in two: the “S” component shows the outcome of simulations in which transformation is symmetrical, and the “A” component shows the outcome of simulations in which transformation is asymmetrical, with the lower fitness allele acquired at a rate 10-fold lower than that of the higher fitness allele. (**B**) Heatmap showing the outcome of simulated competitions between two strains, of which only one is transformable, when there is a cost associated with the expression of the competence machinery. This figure shows the outcome of simulations analogous to those displayed in panel A, except that the transformable strain has a growth rate, γ, 5% lower than the nontransformable strain to represent the cost of the competence machinery. This cost was constant across simulations and independent of the relative fitness difference between the alleles of the exchangeable locus. Raw data are tabulated in S1 Data.

To explore potential benefits of different evolutionary strategies to bacterial cells, a stochastic compartmental model was developed to simulate genetic exchange through HDT (see Methods). The first simulations investigated scenarios in which transformation might facilitate the import of beneficial foreign DNA, corresponding to either the acquisition of novel loci or the reversal of Muller’s ratchet by repairing deleterious mutations. These featured two strains of different fitnesses, one of which was competent for transformation, growing and competing in a homogeneous environment. Increasing the rate of transformation, τ, was advantageous to the transformable strain so long as the nontransformable strain was fitter and, therefore, donating beneficial alleles (Fig 1A). The greater the fitness advantage of the donor, the greater τ needed to be for the transformable strain to acquire the beneficial allele before it was outcompeted. However, when the nontransformable strain was less fit, increasing τ was detrimental to the transformable strain, which could only diversify through the acquisition of deleterious alleles. This situation was exacerbated if transformation has an associated fitness cost, set at 5% in Fig 1B. In these simulations, the cost of expressing the competence machinery meant the transformable strain was outcompeted by a fitter competitor more quickly, limiting the opportunity for the acquisition of beneficial loci, and outcompeted a less fit competitor more slowly, increasing the opportunity for the acquisition of deleterious alleles. Hence, in these simulations, transformation’s ability to facilitate exchange between strains can only provide a long-term benefit to cells if they continuously encounter potential donors of alleles that increase their fitness to an extent that outweighs the cost of expressing the competence machinery, and those beneficial alleles can be acquired more quickly than the transformable cells are outcompeted by the potential donors.

**Fig 2:**
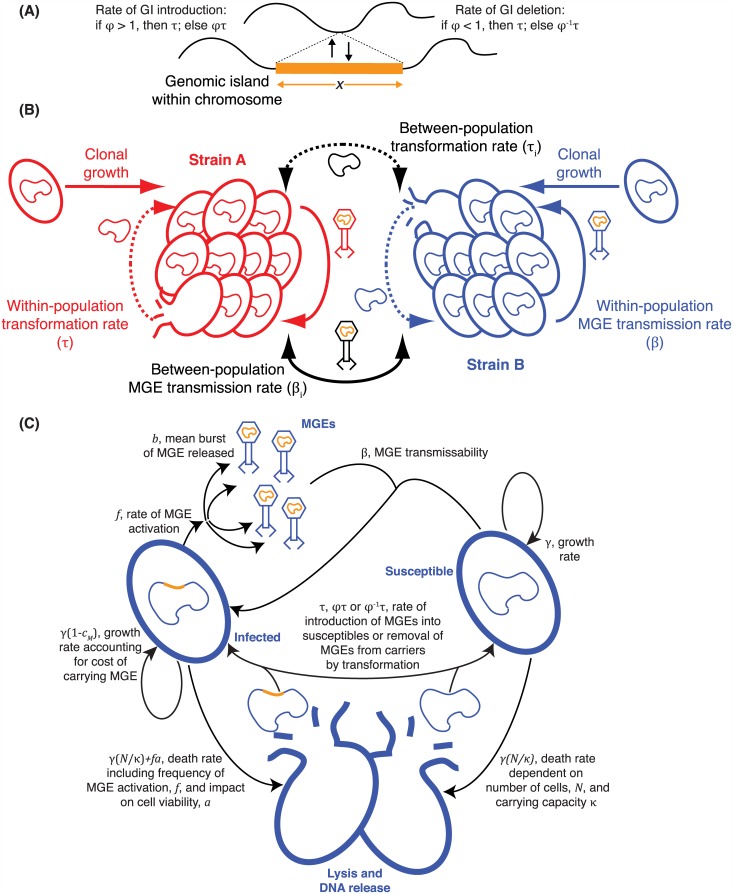
Modelling the interaction of HDT mechanisms in a simple bacterial population. (**A**) Definition of the asymmetry parameter, φ. (**B**) Illustration of the exchange of DNA within and between clonally related cells. (**C**) Description of the stochastic compartmental model of within-population HDT.

The symmetrical shuffling of alleles in this scenario only occurs if donor and recipient have similarly sized alleles of a shared locus. However, homologous recombination is able to transfer DNA when there is similar sequence only at both ends of an otherwise divergent locus, even if the intervening sequence is the length of a typical genomic island [60]. Homologous recombination can, therefore, change a genome’s content through integrating or deleting components of the accessory genome, depending on whether the intervening locus is present in the imported DNA or recipient genome, respectively (Fig 2A). If such allelic variation in the length of a locus exists, then all else being equal, transformation has a bias towards shorter alleles, resulting in a tendency to delete, rather than import, sequence [90–93]. This is a consequence of the necessity for similarity at each end of an imported DNA fragment for it to be stably integrated into a chromosome; any cleavage that separates one of these regions from the other, which becomes increasingly likely as the distance between the homologous arms lengthens, prevents the integration of the entire intervening imported sequence [94].

The parameter φ is included in our model to represent the asymmetric integration of alleles of different lengths (model parameters are summarised in S1 Table). When φ = 1, both alleles of such loci are exchanged at an equal rate (symmetrical transformation); if φ < 1, then the shorter allele is imported at a higher rate than the longer allele, a situation reversed if φ > 1. Assuming imported DNA fragments have a geometric length distribution with a rate parameter of λ_R_ bp^-1^ [60], then φ is a function of an insertion’s length, *x*, of the form *φ*_*x*_ = (1-*λ*_*R*_)^*x*^. The simulations were run as previously described, with a transformable and nontransformable strain initially distinguished by a biallelic locus, with one strain having a neutral short allele and the other a long allele associated with a fitness cost. The transformable cells acquired long alleles at a rate of φ = 0.1 relative to the short allele (see Methods). In simulations in which the deleterious insertion was initially present in the transformable strain, transformation was beneficial in facilitating removal of the locus. However, increasing rates of transformation were neutral when the transformable strain was fitter, as any rise in the rate at which the longer allele was acquired was compensated for by its more rapid removal, a pattern observed whether or not there was a fitness cost associated with the expression of the competence machinery (Fig 1A and 1B; S1 Data). Hence, asymmetric transformation can purge genomes of deleterious insertions.

### Asymmetrical Transformation Counteracts MGE Spread

In these simulations, the deleterious long alleles were eventually removed from the population by transformation; following their elimination, there is no longer a benefit to retaining the competence system. However, deleterious insertions are continuously generated within bacterial populations through MGE movement [57]. We extended the stochastic compartmental model of bacterial HDT such that the deleterious MGEs transmitted horizontally within a community of cells at a rate parameterised by β, assumed to be much faster than the rate of MGE transmission between communities (β_i_; Fig 2B and 2C). The model simulated the growth, competition, and DNA exchange between susceptible (S) and infected (I) bacteria, isogenic except for a single locus at which an MGE was present in the I bacteria. Transformation was only able to eliminate MGEs when they were integrated into the host chromosome. MGEs excised themselves from the host chromosome at rate *f*, which determined the rate at which they transitioned from vertical to horizontal transmission. Simulations were conducted with two MGE types: “more horizontal” (MH; β = 10^−6^ unless stated, *b* = 10, *f* = 0.05, *c*_M_ = 0.075, *a* = 1), which frequently transmitted between cells, and “more vertical” (MV; β = 10^−3^ unless stated, *b* = 5, *f* = 0.005, *c*_M_ = 0.0025, *a* = 0), which more stably associated with host chromosomes.

**Fig 3:**
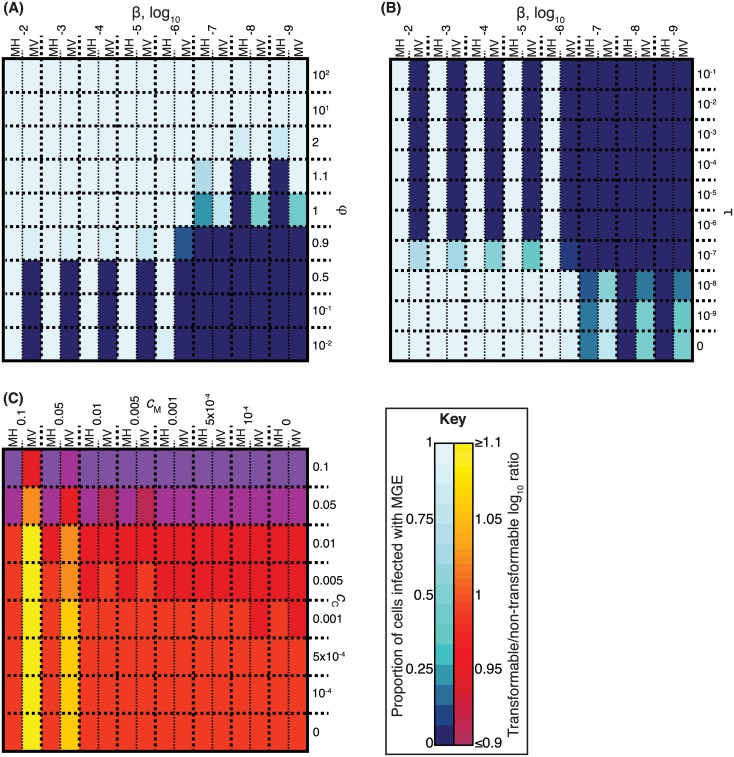
Identifying the necessary conditions for constitutive competence to be effective in inhibiting MGE transmission. (**A**) Heatmap showing the outcome of simulations in which MGEs infect cells competent for transformation at rate τ = 10^−4^. The colour of the heatmap represents the proportion of the population infected by MGEs over the course of each simulation. Each cell represents a specific MGE transmissibility (β) and transformation asymmetry (φ); the “MH” and “MV” components show simulations with MGEs having relatively greater propensities for horizontal and vertical transmission, respectively. (**B**) Heatmap, displayed as in panel A, but this time comparing the effects of varying β against changing the rate of transformation (τ) with a fixed value of φ = 0.1. (**C**) Conditions necessary for transformation to provide a fitness advantage to the population. This heatmap shows the ratio of the total number of cells in two independent sets of simulations: one in which cells were nontransformable, and another in which the cells were transformable and suffered an associated cost (*c*_C_). In both sets of simulations, either the MH or MV MGEs were present in the populations, each of which was associated with a varying cost to the host (*c*_M_); transformation was only effective at inhibiting the transmission of MV. The higher the value of the ratio, indicated by the heatmap colour, the relatively greater the size of the bacterial population when cells were transformable. Raw data are tabulated in S1 Data.

The first set of simulations (Fig 3A) compared different φ values against MGEs of varying transmissibility, with a fixed transformation rate of τ = 10^−4^. We found that simulations with φ > 1, which favoured the transfer of insertions, actively drove the spread of MGEs through the cell population. However, when asymmetry favoured the import of shorter alleles (φ < 1), transformation was highly effective at inhibiting the spread of MH MGEs over a narrow range β values, and MV MGEs across all tested β values. Similarly, when φ = 0.1, higher rates of τ were found to be more effective against MV across all tested values of β, whereas MH could still spread through the population if sufficiently transmissible (Fig 3B). Sensitivity analyses indicated elimination of MGEs was dependent upon a sufficiently high concentration of extracellular DNA for homologous recombination, facilitated by higher cell growth rates and greater stability of extracellular molecules (S1 Fig).

This mechanism was also found to provide a benefit at the level of the population. Independent simulations were run in which cells were either nontransformable or transformable (φ = 0.1, τ = 10^−4^) and, therefore, affected by an associated cost of expressing the competence machinery (*c*_C_). Each population featured either MH or MV, with the associated cost of being infected by such an MGE, *c*_M_, varying between simulations. The heatmap summarizing the ratio of the total cell population recorded from the simulations with identical parameterization, but differing in whether or not the competence machinery was expressed, is shown in Fig 3C. In simulations involving MH, against which transformation was ineffective, either the total number of cells was similar in the matched sets of simulations, if *c*_C_ was negligible, or the nontransformable cells were detectably more successful, if *c*_C_ was high. However, transformation was effective at inhibiting the spread of MV, and, therefore, in simulations involving this MGE in which *c*_M_ was greater than *c*_C_, transformation resulted in the cell population being more numerous when transformable, despite the associated costs of the competence machinery. Hence, constitutive asymmetric transformation inhibits the vertical transmission of deleterious MGEs, thereby allowing bacteria to purge such parasites from their genomes, potentially resulting in increased fitness of individual cells and populations.

### Transient Competence Removes Deleterious MGEs

**Fig 4:**
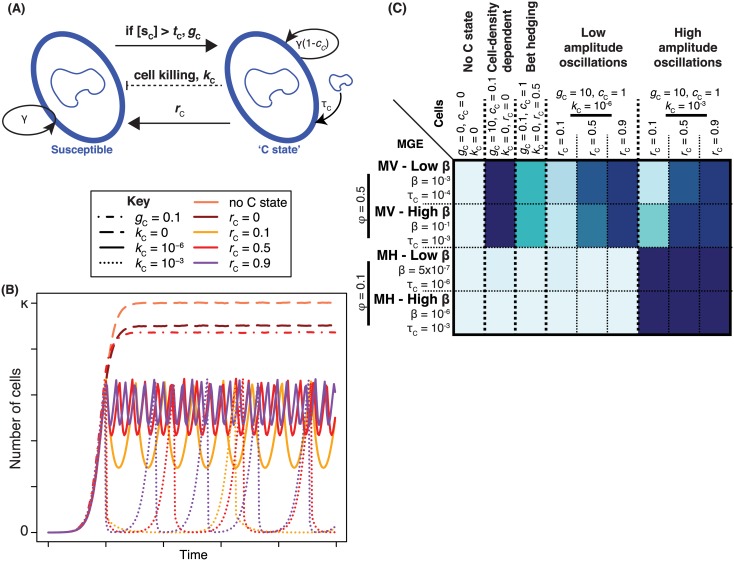
Inhibition of MGE transmission by transient competence. (**A**) Model of a transient competent (“C”) state and cell–cell killing of non-C-state cells. (**B**) Graph showing the original cell growth curve (no C state), cells entering the C state in a cell-density-dependent manner, then never leaving (*k*_C_ = 0 and *r*_C_ = 0); a “bet hedging” strategy (*k*_C_ = 0, *g*_C_ = 0.1, *r*_C_ = 0.5) in which only a fraction of the population is competent for transformation at any one time; cells undergoing small population oscillations (*k*_C_ = 10^−6^) and cells undergoing large population oscillations (*k*_C_ = 10^−3^) at frequencies determined by *r*_C_. (**C**) Heatmap summarising the outcomes of simulations comparing patterns of growth and competence expression in panel B with different MGEs. Colours are scaled as in Fig 3. Results for two representatives of MH and MV are shown, each associated with different rates of HDT. Transformation at the specified τ_C_ rate occurred in the C state, such that the cells that never entered the C state were never competent for transformation. Raw data are tabulated in S1 Data.

In many species, competence is only transiently expressed, rather than being constitutive. To test whether these expression patterns are compatible with inhibiting the spread of MGEs, populations were simulated in which cells were only competent in a “C state.” As competence is often regulated by diffusible signals, cells were assumed to enter C state upon an intercellular signal, a “C signal” (*s*_C_) constitutively generated by all cells, surpassing a threshold, *t*_C_. C-state cells suffered a cost to expressing competence, quantified as a growth inhibition *c*_C_, and exited C state at the rate *r*_C_ (Fig 4A). These parameters were sufficient to define a cell-density-dependent C state (*g*_C_ = 10, *r*_C_ = 0, *c*_C_ = 0.1), in which cells became irreversibly competent above a particular density threshold, and a “bet hedging” strategy (*g*_C_ = 0.1, *r*_C_ = 0.5, *c*_C_ = 1), which resulted in a dynamic equilibrium in which approximately 13% of the population was in C state, with their growth completely arrested, at any point in time. Neither caused much change in the overall growth pattern of the population (Fig 4B).

C state can also mimic “fratricide” or “cannibalism” [15]. Lysis of surrounding bacteria, which are usually near-isogenic clonal descendants of a recent common ancestor, may be important in raising the concentration of DNA available for transformation. To explore the biological significance of this, C-state cells were enabled to kill non-C-state cells at a rate *k*_C_ (Fig 4A). This inclusion of transient arrest of cell growth and cell–cell killing (*c*_C_ = 1, *k*_C_ > 0) resulted in oscillatory growth patterns in which populations went through alternate phases of clonal growth and competence. At *k*_C_ = 10^−6^, competence was transient but associated with little change in population size, while *k*_C_ = 10^−3^ drove large population oscillations. Increasing *r*_C_ had the opposite effect on oscillation amplitude and frequency (Fig 4B). Large, unexplained population oscillations have been observed during the growth of *S*. *pneumoniae* in a chemostat in the absence of phage [95,96], and their occurrence during carriage could explain the discrepancy between census and effective population sizes in animal models [97].

Like constitutive competence, transient competence was also effective in inhibiting the spread of MV (Fig 4C). Irreversible cell-density-dependent C state eliminated MV from the population more quickly than bet hedging, although the latter strategy confined the costs of expressing competence to only a subset of the population. Oscillatory growth patterns were effective when transformation was rapid, necessitating a high value of τ and sufficient DNA release to sustain transformation throughout the competence period. This was facilitated by the cycles of cell–cell killing and rapid growth driven by *k*_c_ > 0, and a shorter period during which competence-associated arrest of growth applied (S2 Fig). By contrast, the spread of MH was only inhibited by large oscillations in cell population size (Fig 4C), also driven by higher values of *k*_C_, although this effect was contingent upon the MGEs being relatively unstable outside the cell, such that they could not survive extracellularly from one population boom to the next (S3 Fig). Hence, we find that coregulation of cell–cell killing with asymmetric transformation, as modelled by this C state, can synergistically combine to block the transmission and spread of MGEs. Horizontal transmission is limited by the large reductions in the density of susceptible cells during population crashes, which are contemporaneous with large releases of DNA that can limit vertical transmission through transformation. Importantly, all simulated patterns of growth and competence were found to be compatible with the elimination of parasitic MGEs.

**Fig 5:**
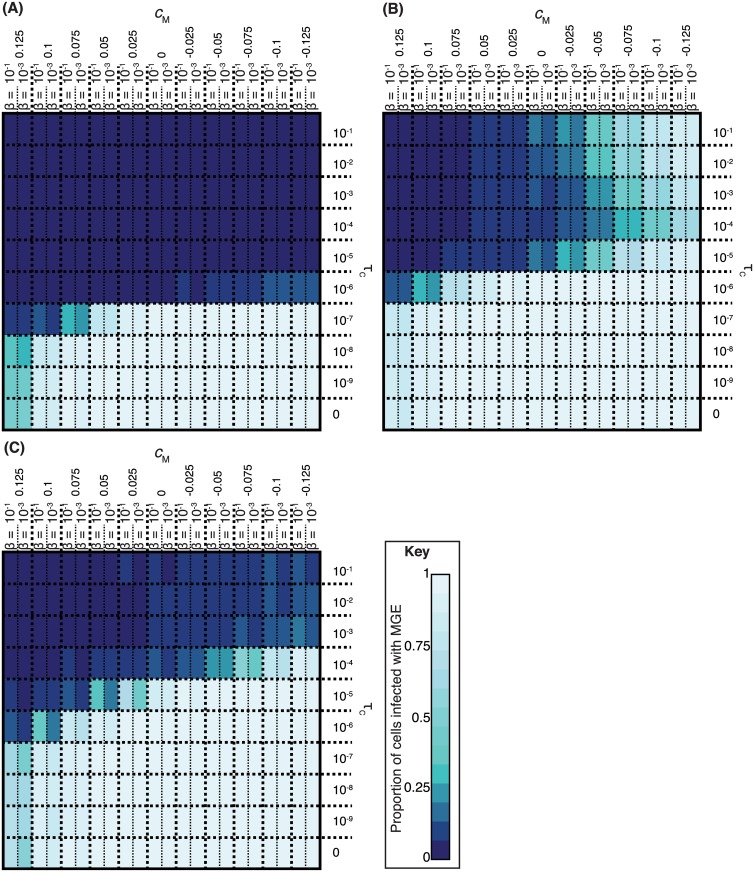
The effect of different patterns of competence expression on the transmission of MGEs that benefit their host. (**A**) Heatmap summarising the outcome of simulations in which MV-type MGEs infected cells that entered the C state in a cell-density-dependent manner (*r*_C_ = 0). Each cell represents a specific transformation rate τ_C_ and “cost” of the MGE (*c*_M_); negative costs imply the MGE benefits the cell. Each cell is split into two components, representing an MV MGE with high (β = 10^−1^) or low (10^−3^) transmissibility. (**B**,**C**) Heatmaps, displayed as in panel A, but comparing cells transiently entering the C state with *k*_C_ = 10^−6^ (panel B) or *k*_C_ = 10^−3^ (panel C; *r*_C_ = 0.9 and φ = 0.5 in both cases). Compared to cell-density-dependent C state, these patterns of C state expression were still effective at inhibiting the spread of MV that were detrimental to the cell, but were a relatively lower impediment to the spread of MV that benefitted the cell. Raw data are tabulated in S1 Data.

Some MGEs have spread successfully while carrying cargo, such as antibiotic resistance, that can be beneficial to their bacterial host [4,84]. To explore the spread of beneficial MGEs, we allow fitness “costs” to be negative, corresponding to advantages that increase the replication rate. Fig 5 shows the spread of beneficial and deleterious MV when the cell population undergoes irreversible density-dependent competence (Fig 5A), low-amplitude oscillations (Fig 5B), or high-amplitude oscillations (Fig 5C). Constitutive, density-dependent transformation is the most effective at eliminating MV, regardless of whether they are detrimental or beneficial. By contrast, transient competence is able to remove deleterious MV but permit those that are beneficial to the cell to spread over multiple orders of magnitude variation in τ. This is the consequence of the MGEs increasing in frequency between phases of competence through both transmission and selection. However, at the population level, asymmetric transformation is still required to preferentially acquire beneficial alleles, with tightly regulated expression of competence alone being insufficient (S3 Fig). Hence, transiently competent populations biased against acquiring insertions through transformation can often allow beneficial MGEs to spread, while eliminating deleterious MGEs.

### Frequent Opportunities for MGE Removal by Transformation

For cells to derive a benefit from transformation acting to remove MGEs, there must be frequent instances of deleterious MGEs being polymorphic within otherwise isogenic cell populations. We explored the potential for such situations within a dataset of over 3,000 sequenced isolates of *S*. *pneumoniae*, of which 1,715 represented longitudinal sampling of 371 hosts [98]. By performing a systematic search for variation in the three major classes of MGEs found in the pneumococcus (phage, integrative conjugative elements, and phage-related chromosomal islands [PRCI] [57]), we uncovered multiple instances of MGE variation between closely related isolates from the same host sampled on different days, despite the comparative insensitivity of sampling a single colony per timepoint. All but one of the well-characterised examples involved changes in prophages, which integrate into the genome and do not typically contain beneficial cargo genes in *S*. *pneumoniae* [57].

**Fig 6:**
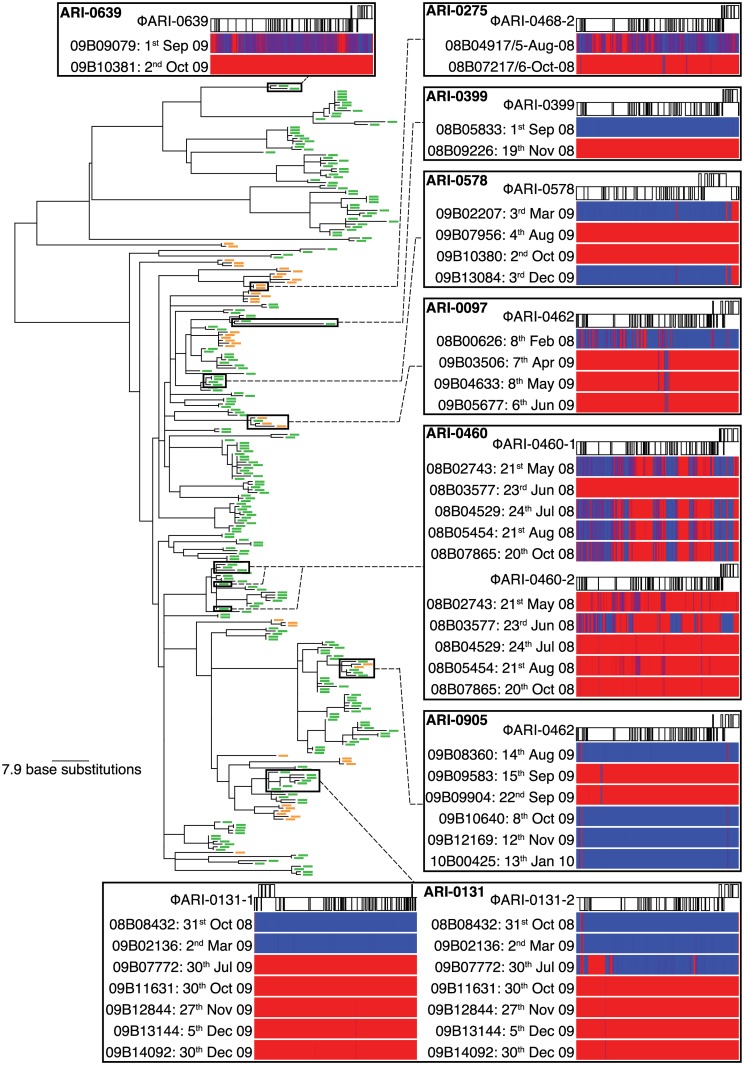
Evidence for coexistence of susceptible and infected BC1-19F cells within individual carriage episodes. The displayed phylogeny was generated based on point mutations, excluding base substitutions likely to have been introduced by recombination (S4 Fig). Leaf nodes are annotated to indicate whether the *comYC* gene, required for efficient transformation, is intact (green dash) or disrupted by a prophage insertion (orange dash; see key in Figs 8 and S5). Specific cases of changes in prophage content within what are likely to represent individual carriage episodes are highlighted. Each box displays the annotation of a specific prophage (S2 Table), along with sequence read mapping heatmaps beneath showing the depth of coverage across the viral sequence for individual isolates from a single host: blue for low levels of mapping, indicating the sequence is absent, and red for high levels of mapping, indicating it is present (see scale in Fig 8). The isolates are ordered by the date of isolation. Epidemiological data are summarised in S3 Table.

Phylogenies were constructed that accounted for divergence through interstrain transformation, thereby reconstructing clonal descent (S4 Fig) [99]. Such analysis of the most common lineage in the sampled population, BAPS cluster (BC) 1-19F, demonstrated that the MGE variation within carriage represented changes in the prophage content of stably carried bacteria with very similar core genomes, rather than a host acquiring new genotypes with stable MGE content (Fig 6, S2 and S3 Tables). The detectable frequency of integrative MGE acquisition indicates these otherwise isogenic, recombining populations will frequently consist of coexisting S and I cells. That this coexistence may persist over weeks or longer is suggested by the repeated identification of the same MGE within a particular host, contrasting with its absence from other timepoints during individual carriage episodes. However, many bacteria were nonlysogenic, and there is little evidence of prophages being conserved over substantial proportions of the lineage’s overall evolutionary history, consistent with selection against cells infected with these parasites (S5 Fig) [57,100,101].

The precise mechanisms by which the MGEs may be eliminated are difficult to differentiate in populations with few distinguishing genetic markers. However, in one of the rare cases in which interstrain DNA transfer was detected within a single carriage episode, a transformation event was associated with the loss of an otherwise stable PRCI (S6 Fig) [57]. This observation of an MGE apparently being removed by transformation demonstrates the feasibility of the mechanism underlying our model.

### Recurrent Inhibition of Transformation by Prophage

The leaf nodes of the phylogenies shown in Fig 6 are marked according to the status of their *comYC* gene, required for effective transformation [4,15]. S5 Fig shows that all these instances of *comYC* disruption result from the insertion of a prophage into the coding sequence (CDS). Whereas the integrases of most MGEs target them into the small noncoding fraction of the bacterial genome, thereby minimising the selective cost imposed on the host, this was the only example of an MGE disrupting a CDS observed in genomic data from a *S*. *pneumoniae* population [57]. Past investigation of resistant *S*. *pneumoniae* have found a second instance of a genomic island disrupting a CDS; a gene cassette encoding a *mefA* macrolide resistance determinant was observed to inhibit transformation through insertion into *comEC* [102], which encodes a protein critical for forming the DNA import pore.

**Fig 7:**
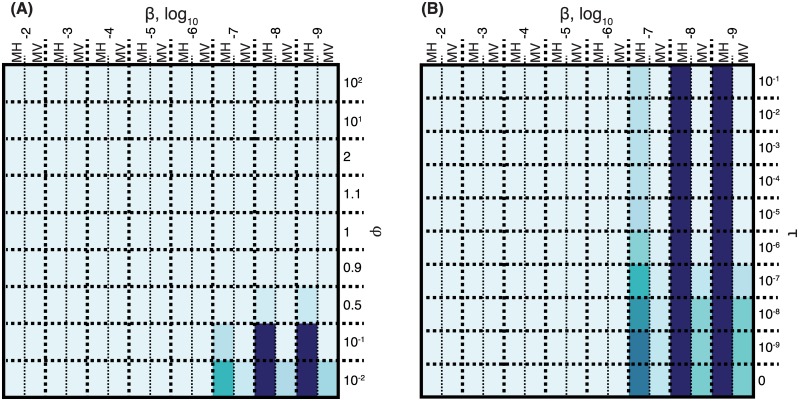
The benefit to MGEs of inhibiting rapid, asymmetric transformation. Panels **A** and **B** repeat the simulations displayed in Fig 3A and 3B, respectively, with the difference that the MH and MV MGEs in these simulations disrupt the host’s ability to undergo transformation when they insert into the cell’s chromosome. Raw data are tabulated in S1 Data.

The benefit to the MGE of disrupting the competence system of the host cell is illustrated in Fig 7A and 7B. These display repeats of the simulations shown in Fig 3A and 3B, except that infection by either MH or MV prevented their host undergoing transformation. The results indicate MGEs derive an advantage from such abrogation of host competence when the transformation rate was sufficiently high (τ > 10^−7^), and asymmetry in favour of shorter alleles sufficiently strong (φ ≤ 0.1), to impede their transmission through the population. Hence, in this model, transformation’s potential to prevent the spread of MGEs provides the selection pressure for such mobile elements to target competence genes for disruption when they integrate into the genome.

**Fig 8:**
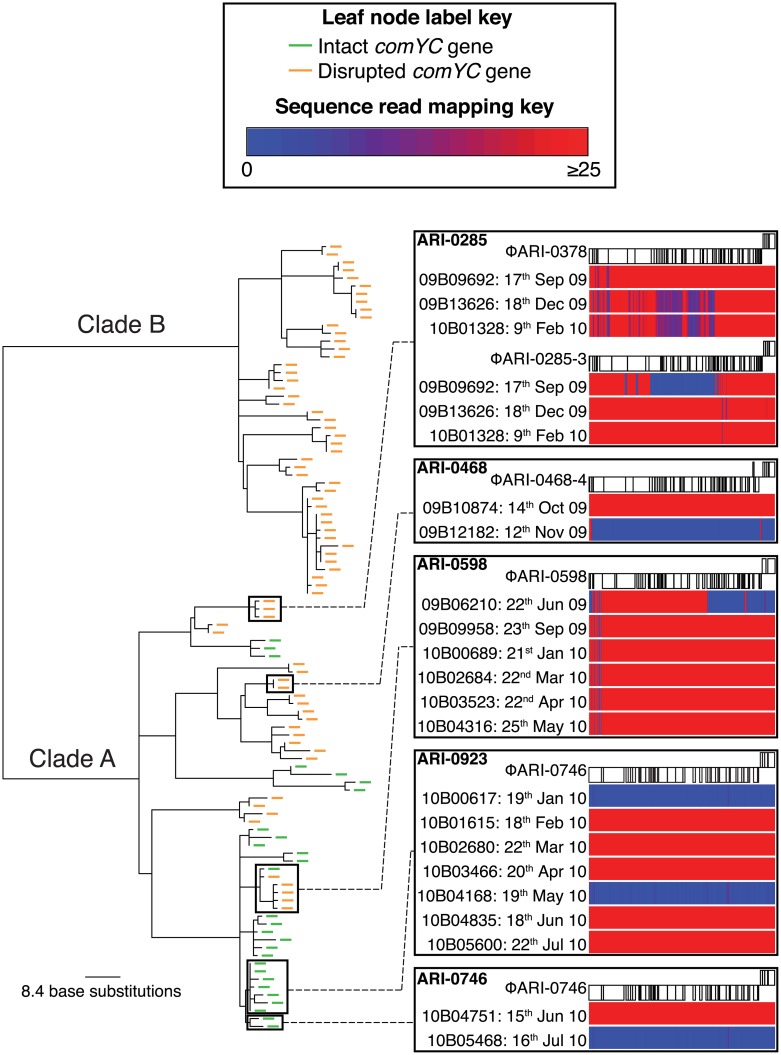
Evidence for coexistence of susceptible and infected BC4-6B cells within individual carriage episodes. The displayed phylogeny was generated based on point mutations, excluding base substitutions likely to have been introduced by recombination (S7 Fig). Leaf nodes are annotated to indicate whether the *comYC* gene, required for efficient transformation, is intact or disrupted by a prophage insertion (see key and S8 Fig). Specific cases of changes in prophage content within what are likely to represent individual carriage episodes are highlighted. Each box displays the annotation of a specific prophage (S2 Table), along with sequence read mapping heatmaps beneath showing the depth of coverage across the viral sequence for individual isolates from a single host, ordered by the date of isolation. Epidemiological data are summarised in S3 Table.

A second lineage from the same population, BC4-6B, included clade A that diversified similarly to BC1-19F, with intermittent import of diversity through transformation and disruption of *comYC* (Fig 8). Yet clade B shows little evidence of diversification through transformation or MGE variation (S7 and S8 Figs). This reduction in all HDT mechanisms is associated with the stable inheritance of two prophages within clade B, one of which disrupts *comYC* (S8 Fig), exemplifying the efficient vertical transmission of prophages in the absence of transformation. However, as is necessary for competence to be preserved in the species [74], most insertions into *comYC* were not successful. Instead, across BC1-19F and clade A of BC4-6B, noncompetent bacteria appear to have been removed by selection at a faster rate than diversification through interstrain transformation, with “clonal” lineages only rarely found to transmit between multiple hosts.

### MGEs Inhibit Transformation in Many Species

**Fig 9:**
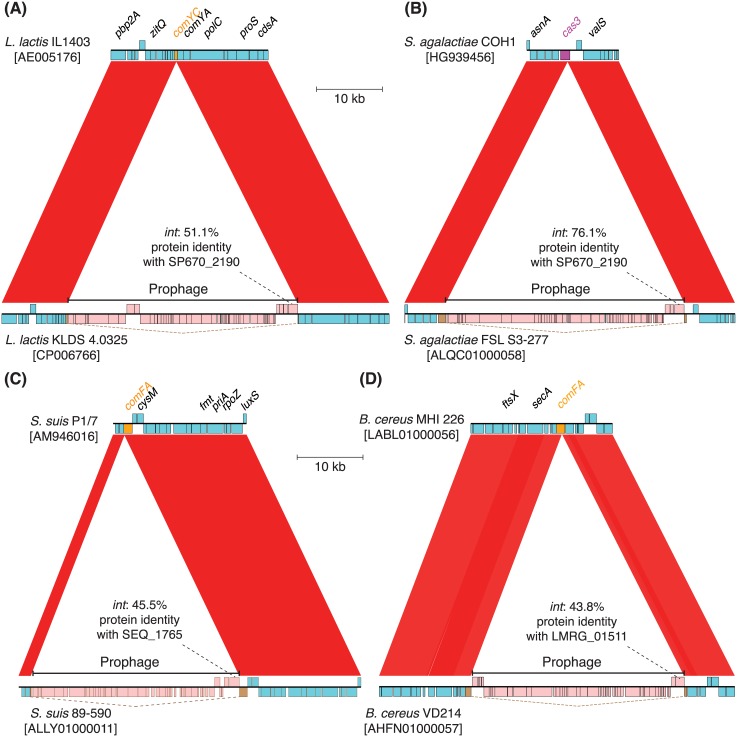
Selected examples of MGE insertions disrupting chromosomal protein coding sequences. (**A**) Comparison between *Lactococcus lactis* isolates IL1403 and KLDS 4.0325, the latter of which has a prophage inserted into the *comYC* gene, encoding the major structural component of the competence pilus. The sequences’ accession codes are given in brackets underneath the isolate names. Blue and orange boxes represent CDSs, with the direction of their transcription indicated by their vertical position relative to the horizontal line; pink boxes indicate putative MGE CDSs in the same way. Brown boxes linked by dashed lines mark the fragments of a pseudogene disrupted by MGE insertion. The red bands link regions of similar sequence in the two loci, as identified by BLAST-like alignment tool (BLAT); the intensity of the colour indicates the strength of the match. The prophage integrase has ~51% identity with the protein that drives integration into the orthologous gene in *S*. *pneumoniae* 670-6B (SP670_2190). (**B**) Comparison between *Streptococcus agalactiae* isolates COH1 and FSL S3-277, the latter of which has a prophage inserted into the *cas3* gene of the *S*. *agalactiae* CRISPR2 locus. This prophage integrase is ~76% identical with that of the prophage disrupting the *comYC* gene of *S*. *pneumoniae* 670-6B. (**C**) Comparison of *Streptococcus suis* isolates P1/7 and 89–590, the latter of which has a prophage inserted into the 3’ half of the *comFA* competence gene. This prophage integrase is ~46% identical with that of the prophage inserted into the orthologous gene in *Streptococcus equi* (SEQ_1765). (**D**) Comparison between *Bacillus cereus* isolates MHI 226 and VD214, the latter of which has a prophage inserted into the 5’ half of the *comFA* competence gene. The prophage’s integrase is ~44% identical with that of the prophage inserted into the *comK* competence gene in *Listeria monocytogenes* (LMRG_01511).

The insertion of a prophage into *comYC* is not restricted to *S*. *pneumoniae*. Searches for orthologues of the relevant phages’ integrases, which determine the site into which a phage inserts, identified examples of a prophage targeting the same gene in several other related species; these included *Streptococcus mutans* and *Streptococcus parauberis*, from the same genus, as well as an example in *Lactococcus lactis* (Figs 9A and S9). These all inserted at an orthologous, but not perfectly conserved, site within the gene (S10 Fig). Unexpectedly, an orthologous integrase was found in *Streptococcus agalactiae*, a species not considered to be naturally competent (Fig 9B) [28,103]. This integrase was part of a phage that inserted into the *cas3* gene of *S*. *agalactiae*’s CRISPR2 locus [104]. While disruption of loci that inhibit infection of the cell would not be expected to benefit a prophage, CRISPR systems are capable of targeting integrated prophages, resulting in cell suicide, or the post-activation excised, replicating form of the phage [105–107]. Under either scenario, depleting the cytosol of functional CRISPR proteins would provide the phage with an advantage prior to transmitting horizontally to the next cell. Hence, cellular countermeasures to both defences against horizontal and vertical transmission of MGEs are targeted by similar integrases directing the insertion of prophages in streptococci and related genera.

A different example was observed in *Streptococcus equi* [108], in which a prophage inserted into the *comFA* gene. Orthologues of this distinct integrase were identified in other species including the zoonotic pathogen *Streptococcus suis*, in which the protein again directed a prophage to insert into the host cell’s *comFA* gene (Fig 9C). Similarly, some strains of *Listeria monocytogenes* have a prophage inserted into their *comK* genes [109], encoding a regulator of competence. A similar insertion was identified in a representative of *Listeria innocua* [110], a further example of which is shown in S11 Fig. Searching for proteins similar to the *L*. *monocytogenes* prophage integrase identified a prophage inserted into *comFA* in representatives of the *Bacillus cereus* and *thuringiensis* group (Figs 9D and S11). However, codon alignments demonstrated that the insertion site within the *Bacillus comFA* genes was distant from that of the distinct prophage identified in *S*. *suis* (S10 Fig), suggesting the targeting of this gene represented convergent evolution between the two MGEs. Another orthologue of the integrase found in *L*. *monocytogenes* was present in a phage of *Enterococcus faecalis*, this time targeting the MGE to insert into *radC* (S11 Fig); the same gene was reported to be targeted by a prophage inserted into *B*. *subtilis* that inhibited transformation [111], although the insertion site was not demonstrated to be the cause of this inability to integrate exogenous DNA. While RadC levels increase during competence, it does not always appear to be essential for transformation in the laboratory [112].

Another previously identified example of MGEs inhibiting competence was the observation from *Aggregatibacter actinomycetemcomitans* genomes that some MGEs inserted into *comM* [113], which encodes a protein important for efficient incorporation of DNA into the chromosome through homologous recombination [22]. MGEs were also inserted into *comM* in a single representative of *Acinetobacter baumannii* [114], and a large insertion was identified in the same gene in *Mannheimia succiniciproducens* [115]. Searching for orthologues of the integrase targeting *comM* from *A*. *actinomycetemcomitans* identified examples in other strains of *A*. *baumannii* (S12 Fig), and similar insertions were observed in genes encoding orthologues of ComM across a diverse set of species, including the animal pathogen *Mannheimia haemolytica*, the human pathogen *Francisella philomiragia*, and the plant pathogen *Pseudomonas syringae*.

### The Effect of Competition between MGEs

**Fig 10:**
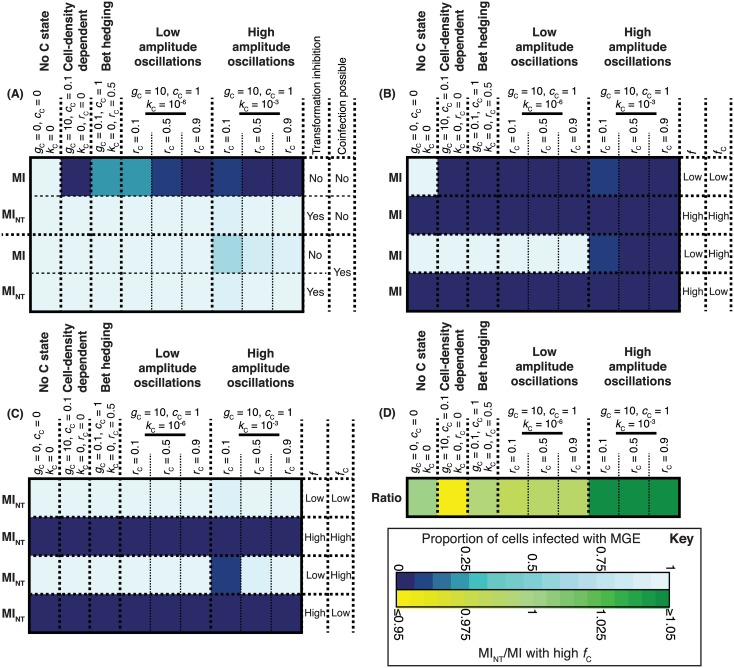
The effectiveness of MGE strategies for reducing elimination by transformation. (**A**) Heatmap summarising the outcomes of simulations comparing the patterns of cellular competence shown in Fig 4C in the presence of the MGE MI (top row), which has properties intermediate between those of MH and MV. The colour of each cell represents the proportion of the population infected by the MGE over the course of the simulations. In the second row, the same simulations are performed, but in this case the MGE MI_NT_ inhibits transformation in the host cell. The bottom two rows show the outcome of simulations in which both MI and MI_NT_ infect the same population. (**B**) Heatmap summarising the outcomes of simulations comparing cell growth patterns with different MI activation patterns: *f*, the normal rate of activation, is either low (0.005) or high (0.5), as is *f*_C_, the rate of activation in C state. (**C**) Heatmap summarising the same simulations shown in panel B, but for MI_NT_. (**D**) Competition between MI_NT_ operating with its optimal strategy (*f* and *f*_C_ low) and MI operating with its optimal strategy (*f* low, *f*_C_ high). Both MGEs were allowed to infect the same population in these simulations. The heatmap shows the ratio of strains infected with MI_NT_ to those infected with MI when cells grew and expressed competence for transformation under different strategies. Raw data are tabulated in S1 Data.

Despite the potential advantage to individual mobile elements, the disruption of competence by MGEs is not ubiquitous. To investigate the reasons underlying this, we modelled an MGE “MI,” intermediate in properties between MV and MH (β = 5×10^−6^, *b* = 7, *f* = 0.01, *c*_M_ = 0.005, *a* = 1), which is able to spread through a nontransformable cell population but is eliminated by cell-density-dependent, bet-hedging, and transient patterns of competence expression (Fig 10A). However, “MI_NT_,” which has the same properties but disrupts the competence system of the host cell, is able to spread regardless of the type and rate of transformation of the host cell. When MI and MI_NT_ coinfect the same cell population, both MGEs achieve similar levels of transmission, mirroring the stability of both prophages within BC4-6B clade B, despite only one inhibiting competence (S8 Fig). This limits the advantage of strategies such as disrupting *comYC*, as the affected cell cannot eliminate superinfecting MGEs, benefitting all other elements infecting the same host.

Individual MGEs can also escape the consequences of transformation by switching from vertical to horizontal transmission when a cell becomes competent. This is achieved through increasing the activation rate, *f*, to an elevated value *f*_C_ during C state. Evidence for the relevance of this mechanism is the observation that many prophages [116–118] and integrative and conjugative elements (ICEs) [119,120] excise from the chromosome in response to elevated levels of RecA, the protein required for homologous recombination. Simulating the spread of MI with low (*f* or *f*_C_ = 0.005) or high (*f* or *f*_C_ = 0.5) rates of activation during either clonal growth or C state shows that relying heavily on vertical transmission (*f* = *f*_C_ = 0.005) makes the MGE highly susceptible to transformation (Fig 10B). By contrast, high levels of activation outside of C state (*f* = 0.5) results in substantial costs to the host population, with the consequent low host cell density reducing the efficiency of horizontal MGE transmission (S13 Fig). However, activating only at a high rate during C state permits stable vertical transmission when cells are not competent, while greatly reducing elimination through transformation. Nevertheless, cells’ synergistic coupling of reduced cell density with transformation can still strongly inhibit the spread of MGEs adopting this optimal strategy.

To test whether both these strategies for avoiding elimination by transformation might be synergistic, the analysis of activation rates was repeated for MI_NT_. This found that the optimal strategy for MI_NT_ was different; as the MGE could not be removed by transformation, it could achieve fixation in a population without elevated rates of activation in C state, and instead benefitted from a low rate of activation regardless of the cell’s behaviour (Fig 10C). However, when both MI and MI_NT_ were allowed to compete in the same population, both operating under optimal strategies (*f* low and *f*_C_ high for MI; *f* and *f*_C_ low for MI_NT_), MI was more successful in a greater number of scenarios (Fig 10D). MI_NT_, by contrast, was more successful only if there were large changes in population size that inhibited horizontal transmission. This was the consequence of the rapid activation of MI, following the onset of C state, allowing it to spread horizontally at a higher rate; additionally, this behaviour facilitated killing C-state cells in which MI_NT_ was also inserted but not yet activated. Hence, elevated *f*_C_ provides an advantage to MGEs over competitors in the same cell, regardless of whether they inhibit transformation or not. This contrasts with the disruption of host cell competence, which is an effective strategy for individual MGEs, but provides the same benefit to all MGEs parasitizing the same host. Hence the effectiveness of transformation against MGEs is likely to be partly maintained by competition between MGEs that infect the same cell.

### Transformable Streptococci Harbour Fewer Prophages

Our hypothesis predicts that parasitic MGEs integrated into chromosomes that have not achieved fixation should be vertically transmitted at a lower rate in recombining populations of naturally transformable bacteria relative to equivalent nontransformable bacteria. The implication is, all else being equal, that transformable bacteria should harbour fewer MGEs. This is difficult to test with draft assemblies of bacterial genomes, which are fragmentary, often particularly so in regions encoding MGEs; this situation is exacerbated when multiple similar elements are present in the same genome [57]. An alternative approach is to use complete and high quality draft genomes from species or genera containing a mix of transformable and nontransformable bacteria. There are over 140 suitable genome assemblies available for streptococci, of which a subset have been demonstrated to be naturally transformable [28,32,84,121–123]; additionally, *Streptococcus pseudopneumoniae* was assumed to be naturally transformable, based on its close relationship with *S*. *pneumoniae* and *Streptococcus mitis*. In agreement with a previous comment by Beres et al. [124] based on the small number of genomes available at the time, streptococci demonstrated to be naturally transformable had significantly fewer prophages than nontransformable streptococci (mean number of prophages per transformable genome: 0.74; mean number of prophages per nontransformable genome: 1.85; Wilcoxon rank sum test: W = 3065.5, *p* = 0.00021; S4 Table and S14 Fig) despite tending to have larger genomes (mean size of transformable genome = 2,073,450 bp; mean size of nontransformable genome = 2,002,130 bp; Wilcoxon rank sum test: W = 1702, *p* = 0.013; S4 Table). However, there are several caveats to such an analysis. Firstly, the sampling of genomes is nonrandom, something that this analysis partly seeks to address (see Methods). Secondly, all samples are filtered by selection, and, therefore, low-fitness isolates that accumulate large numbers of MGEs are likely to be lost from the population quickly, thereby limiting the opportunity for sampling. Thirdly, there are other systems that inhibit the transmission of MGEs, thereby making the comparison uncontrolled; it is noteworthy, for instance, that an analogous comparison of MGEs and CRISPR systems in streptococci did not find evidence for these systems providing a protective effect against phage infection [125]. In another example, a within-species analysis of *A*. *actinomycetemcomitans* cells, of which a subset were rendered nontransformable by MGE insertions into the *comM* gene, reported noncompetent isolates to have an increased susceptibility to further MGE infection, although this was confounded by the associated loss of some CRISPR functionality [113].

## Discussion

The simulations and genomic analyses presented here were motivated by the unanswered question of what function underlies the major evolutionary benefit of genetic transformation. The model structure followed from the assumption that most HDT through transformation would be between clonally related cells, as a consequence of the physical structuring of populations, combined with the observations that imports are skewed toward shorter recombinations [60,85], the import of deletions is highly efficient in the absence of recognition by mismatch repair [59] or restriction-modification systems [89], and prophages are highly variable over short evolutionary timescales [57,101]. The resulting model shows a benefit, at the individual and group levels of selection, when transformation is sufficiently fast and asymmetric to remove deleterious MGEs from chromosomes. This benefit was found to be independent of whether competence was transiently or constitutively expressed; one difference was that intermittent periods of competence alleviated the elimination of some MGEs with beneficial cargo genes. Coordination of competence with cell–cell killing resulted in oscillatory patterns of growth that synergistically inhibited both the vertical and horizontal spread of MGEs. “Bet hedging” could also be effective at inhibiting the transmission of MGEs; elimination of parasitic elements from the chromosome would be beneficial to the competent subpopulation, while the noncompetent, more quickly replicating cells would have the advantage when such MGEs were not present. Models of this behaviour may be improved when the interaction of MGEs with spores and “persister” cells [42] are better understood.

In order to be plausible, the model depended on clonally related infected and uninfected cells coexisting within populations that could exchange DNA through transformation. This was demonstrated to occur using genomic data on within-host bacterial diversity, albeit limited to a single colony per timepoint. Additionally, a likely example of a detectable transformation event removing a chromosomally integrated MGE from an infected cell was identified (S6 Fig). Even if the rate at which transformation events occur at a given locus per unit time is low, there is the opportunity for a homologous recombination event to be beneficial through removing an inserted MGE up until the point at which it activates or becomes fixed in the local population; this contrasts with the hypothesised role of transformation in the repair of dsDNA breaks, for instance, as in these circumstances homologous recombination is only beneficial if it affects the damaged locus before repair by other means or the next round of chromosomal replication occurs.

However, that our hypothesised mechanism is possible and potentially of benefit to cells does not necessarily imply that it is the primary biological role of transformation. The extent to which cells benefit from this function may be inferred from the countermeasures employed by MGEs to avoid elimination by homologous recombination. It is highly unlikely that any function other than transformation is targeted, based on the plethora of competence genes disrupted by MGE insertions: preventing transcriptional activation of the competence machinery (*comK*); eliminating the major structural component of the pseudopilus necessary for DNA binding (*comYC*), and inhibiting post-binding processing of the DNA (*comFA* and *comM*). The loci at which these MGEs integrate are unlikely to be random [126], particularly in cases such as these in which they are conserved across species and genera despite divergence of the insertion sites (S10 Fig). Additional evidence is the convergent evolution of MGEs inserting into two distinct sites of the *comFA* gene in different genera, driven by divergent integrases with only 34.1% amino acid identity with one another. This is despite MGE insertion sites generally being enriched outside of CDSs [57,126].

Targeting of competence genes for disruption is not the only mechanism by which MGEs inhibit transformation events that may eliminate them from the chromosome. Some encode secreted DNases that degrade exogenous dsDNA. Secreted “streptodornases” have been identified on prophages in a number of streptococcal and lactococcal species [127]. In *B*. *subtilis*, the prophage-encoded DNase YokF was found to decrease transformation rates by an order of magnitude [128]. A search of *Campylobacter jejuni* isolates that transformed at a reduced rate identified the DNase Dns, encoded by the CJIE1 MGE [129]; the presence of this protein reduced transformation rates by three orders of magnitude. Similarly, two further orthologous DNases were identified in the *C*. *jejuni* MGEs CJIE2 and CJIE4, which were each capable of reducing transformation frequencies of their host cells by two orders of magnitude [130]. Recently, an ICE in *V*. *cholerae* encoding the IdeA DNase was found to inhibit transformation by two to three orders of magnitude [131].

These countermeasures are only advantageous to MGEs if transformation inhibits their transmission in natural populations (Fig 7). If the condition that the cost of the competence machinery to cells is less than that of parasitic MGEs is fulfilled (Fig 3C), this implies transformation is likely to provide a net benefit to cells. That MGEs cause a severe cost to their hosts seems a reasonable inference, given the presence of defences against horizontal transmission of MGEs. Analogously, these defences are also part of an arms race, given the existence of MGE-encoded proteins that inhibit restriction modification [132] and CRISPR systems [133], alongside the prophage insertion into a CRISPR locus identified in this work. Therefore, while MGE removal may not be the only activity facilitated by the competence system, there is evidence that it is a function that provides a fitness advantage to the cell. Hence, this role alone may be of sufficient selective benefit to drive the evolution of the necessary machinery. However, our model does not predict that MGEs are likely to become sufficiently adept at preventing transformation that they would render it ineffective over long evolutionary timescales. Only a subset of MGEs would be expected to disrupt the activity of the competence machinery, because in so doing they benefit any superinfecting MGEs that would also likely progressively reduce the fitness of their host cell. The accumulation of further deleterious MGEs could potentially be inhibited by a compensatory improvement in defences against horizontal MGE transmission, which could account for the occasional success of nontransformable, clonally evolving lineages such as clade B of BC4-6B, *S*. *pneumoniae* CC180 [134], and PMEN2 [84].

The atypical stability of the prophage, and, indeed, the rest of the accessory genome, in these clonal *S*. *pneumoniae* lineages is consistent with transformation being important in inhibiting the vertical transmission of viral sequences in bacteria. Nevertheless, there are also alternative mechanisms that can cause the loss of MGEs that should be considered. The first is spontaneous deletion of sequence; this would usually be disadvantageous if occurring at random, as it would remove beneficial sequences far more frequently than detrimental sequences. The second is removal of MGEs by intragenomic recombination events, potentially mediated by the tandem *att* site duplications flanking many mobile elements. However, the length of the *att* sites is generally determined by the MGE, and any *att* sites long enough to trigger these rearrangements would be selected against through their inhibition of vertical MGE transmission. Thirdly, MGEs may drive their own excision. Some conjugative episomes can switch between integrated and extrachromosomal forms [135], the latter of which are resistant to elimination by transformation. However, in the case of prophages, it seems very likely that the majority of excision events result in host cell lysis. This may be inferred from the large population falls observed on mitomycin C addition to lysogenic populations of relevant species [136], and, furthermore, the existence of apparently altruistic cell death from “abortive infection” systems upon phage replication would be undermined if cells frequently survived phage infection [137]. Hence, alongside transformation’s efficiency in deleting DNA [91–94], it is also a very effective mechanism of eliminating integrated MGEs; the imported sequence will restore the uninfected insertion site without affecting flanking regions, which are likely to contain beneficial genes, with no dependency on MGE-encoded loci to facilitate the process.

The speed and frequency with which phage infection is observed to occur in *S*. *pneumoniae*, with competence genes disrupted by a subset of such events, contrasts with the observed population dynamics following the introduction of the anti-pneumococcal polysaccharide conjugate vaccines [138]. This represents the type of environmental change to which transformation has been predicted to speed adaptation [42,46], as strains targeted by the vaccine can evade the effects of immunisation by means of transformation events that switch the bacterium’s serotype through allelic replacement at the relevant genetic locus [139]. There was substantial opportunity for switching to occur, as serotypes targeted by the vaccine nevertheless persisted for several years after the immunisation programme began [138]. Additionally, there was the appropriate motivation for sequence transfer. Although the fitness disadvantage of serotypes targeted by the vaccine was small enough for them not to be immediately eliminated from the population after immunisation began, these serotypes did eventually disappear, suggesting the benefit of acquiring a nonvaccine serotype was greater than the cost of expressing the competence machinery. However, only a subset of the targeted population showed evidence of diversifying in response to vaccination years after immunization had begun, and the examples of serotype switching that could be thoroughly characterised were found to represent the outgrowth of variants that originated prior to the vaccine’s introduction [4,138]. That the non-prophage accessory genomes of pneumococci, including the genes determining serotype, were largely stable post-vaccination [57] confirms it is unlikely that transformation’s primary role is in facilitating adaptation through sequence diversification. Furthermore, such a role is not consistent with a gene-centric view of evolution, as if diversification is the primary purpose of transformation, then the fitnesses of all chromosomal genes in a competent cell are reduced as a consequence of them potentially being replaced by a different allele from the pool of exogenous DNA.

By contrast, this “chromosomal curing” model, in which transformation is primarily a mechanism for maintaining the integrity of a cooperating set of self-replicating genes (be they a chromosome, chromid, or plasmid) against invasion by selfish parasites, is consistent with selection at the level of the gene, individual, and group. If the import of DNA from divergent genotypes is sufficiently rare, then the fitness of individual genes is reduced by a negligible degree, as their probability of being replaced with a different allele is low. However, each gene may frequently benefit from the loss of linkage with a genomic parasite. The model is also able to rationalise the counterintuitive cell–cell killing within clonal populations, which should be strongly opposed by kin selection, as a mechanism to mitigate against the external threat of parasitic MGEs.

In this model, exchanges between diverse genotypes can be viewed as an accidental byproduct of otherwise beneficial exchanges between clonally related cells. This does not preclude some such diversification through HDT being advantageous, particularly after filtering by selection. Hence, cocirculating lineages within naturally transformable species may differ in their rates of diversification through transformation by orders of magnitude, without a substantial fitness difference being evident [138,140]. That the most frequent sequence exchanges are between near-isogenic cells also explains how transformable bacteria can import substantial lengths of DNA in minutes, yet maintain pseudoclonal population structures over decades [57]. Rather than this reflecting the rarity of sequence exchange, such population stability may reflect the continual antagonism between different mechanisms of HDT.

## Methods

### Description of the Microevolutionary Model of HDT

We developed a stochastic compartmental model that included four types of compartments: cells, MGEs, DNA, and a signalling molecule, “C signal.” The overall structure of the model is displayed in S15 Fig.

Bacterial cell growth (green arrows in S15 Fig) followed a logistic growth model. In the absence of MGE infections, cells grew at a constant rate γ (set to 0.2 *t* ^-1^, unless otherwise specified). Analyses of the model output sensitivity to different values of γ are shown in S1 and S3 Figs. Cells died at a density-dependent rate (brown arrows in S15 Fig) determined by γ and a carrying capacity, κ (10^6^ in all simulations). For the *k* cell compartments in the model, the number of cells (*N*_i_) in compartment *i* at time *t* changed at time *t*+1 through the demographic processes of birth and death by *P*_i_, which was distributed as:

$$P_{i,t+1}\sim Bin\left(\gamma .dt,N_{i,t}\right)-Bin\left(\frac{\gamma \sum_{j=1}^{j=k}N_{j,t}.dt}{\kappa },N_{i,t}\right)$$

In all simulations, the starting inoculum of each distinct genotype was 100 cells. All cell compartments immutably belonged to one of two strains, each of which could be independently parameterised. The “plastic” aspect of cells’ genotypes was defined by two biallelic loci: the first locus could either be “empty” (allele E1) or have an inserted MGE, M1; analogously, the second could be empty (allele E2) or contain a different inserted MGE, M2. Upon density-dependent cell death, one DNA molecule was released from each locus, the type of which depended on the host cell genotype (S15 Fig; blue arrows).

Any cell with an “M1” or “M2” allele therefore carried an MGE and, consequently, grew at a rate γ(1-*c*_M_), where *c*_M_ was the reduced growth of the host cell owing to the cost of the inserted MGE. This factor only applied to the growth term of the demographic model; cells carrying MGEs were killed through cell density-dependent death at the same rate as noncarriers. Analogously, cells infected with two MGEs grew at a rate γ(1-*c*_M1_) (1-*c*_M2_). For cells in the *i*th compartment carrying MGE *M*_q_, the number of MGEs that activated per timestep interval at time *t*, *A*_q,i,t_, was distributed according to the number of cells *N*_i_ at time *t* and activation rate of the MGE type *q* in cell type *i f*_q,i_:

$$A_{q,i,t}\sim Bin\left(f_{q,i}.dt,N_{i,t}\right)$$

The number of MGEs of type *M*_q_ released by activations occurring in cell type *i*, *R*_q,i_, at time *t* (dark blue and purple arrows in S15 Fig) depended on the mean burst size, *b*_q_:

$$R_{q,i,t}\sim Pois\left(b_{q}A_{q,i,t}\right)$$

As *A*_q,t_ determines *M*_q,t_, rather than *M*_q,t+n_ where *n* > 0, the activation and packaging of MGEs is effectively instantaneous in this model. This means there is no eclipse period. If included, this would limit the fitness of horizontal transfer by slowing the rate of transmission between cells, but as this study focused on inhibition of vertical transmission, it did not form part of this model. The parameter *a* determined the consequence of MGE activation for the cell; if *a* = 1, then MGE activation killed the host cell, as is typical for prophages; but if *a* = 0, then MGE activation did not affect the host cell, as is typical for ICEs. As MGE activation involves excision from the host chromosome and packaging of the MGE DNA, any cells killed through this mechanism did not release DNA molecules corresponding to the activated MGE, but the appropriate allele was released from the other locus (dark blue and purple arrows in S15 Fig).

Horizontal DNA transfer was modelled as a two-step process using one of two association parameters: β, the infectivity associated with a particular MGE, and τ, a transformation rate associated with a particular cell type. The noncellular components of the model were removed from the environment at a constant “washout” rate, ω, set at 0.6 *t* ^-1^ unless otherwise specified (grey arrows in S15 Fig). Analyses of the model output’s sensitivity to different values of ω are shown in S1 and S3 Figs. Hence, the overall rate at which noncellular agents were removed from the simulation was a composite of cellular binding and elimination from the extracellular environment. For the *q*th DNA compartment, molecules were removed at a composite rate *r*_q_ representing “washout” and binding to each of the *k* cell compartments, each containing *N*_i_ cells and undergoing transformation at a cell-determined rate τ_i_:

$$r_{q}\left(DNA\right)=\omega +\sum_{i=1}^{i=k}\tau_{i}N_{i}$$

Similarly, for the *q*th MGE compartment, elements were removed at a composite rate *r*_q_ representing washout and binding to each of the *k* cell compartments, each containing *N*_i_ cells, at the MGE-determined rate β_q_:

$$r_{q}\left(MGE\right)=\;\omega +\sum_{i=1}^{i=k}\beta_{q}N_{i}$$

For the *q*th type of noncellular agent in the model, the number of agents of that type (*N*_q_) decreased by *d*_q_ per timestep as determined by the relevant value of *r*_q_:

$$d_{q}\sim Bin\left(1-e^{-r_{q}.dt},N_{q}\right)$$

The noncellular agents were then assigned to cell compartments through a multinomial distribution, which allowed for differences in τ between cell types. Within each compartment, the bound DNA molecules and MGEs were then randomly assigned to individual cells. In cases in which a single cell was bound to multiple noncellular agents, a single noncellular agent was randomly selected for interaction at that timestep; MGEs interacted through causing infection (maroon arrows in S15 Fig), while DNA interacted through causing a transformation event (orange arrows in S15 Fig). This structure permitted a single association constant to parameterise interactions between noncellular agents and cells in a manner that could be limited by either partner in the interaction. However, this structure has the disadvantage of artefactual antagonism between MGEs and DNA in the cases where both are bound to a single cell, but only one is selected to interact with the cell. This effect is small unless there are large numbers of noncellular agents interacting with individual cells per timestep. Simulations mirroring those in Figs 3B and 4C were carried out in which cells preferentially interacted with MGEs if DNA was also bound to the cell, rather than the selection being random; the results are shown in S13E and S13F Fig, demonstrating that any artefactual antagonism between DNA transformation and MGE infection was negligible at the *dt* interval used in all simulations (10^−3^).

In cases in which MGEs were introduced at a constant rate, such as S13A–S13D Fig, the number of MGEs of type *q* entering at each timestep, *E*_q_, was determined by the entry rate, *e*_q_, and the MGE burst size:

$$E_{q}\sim Bin\left(e_{q}.dt,b_{q}\right)$$

The relative rate at which the “M” and “E” alleles at the two loci were exchanged was also determined by the asymmetry parameter, φ. In the case of symmetrical transformation (φ = 1), the rate at which the *i*th cellular compartment underwent transformation was determined by τ_i_ and the number of available DNA molecules. These factors also determined the rate at which any transformation in which the donor and recipient alleles were the same occurred; such recombinations did not affect cell genotype, but nevertheless depleted DNA molecules. When φ > 1, favouring the import of longer alleles, if *B*_i,q_ complexes of a DNA molecule of the *q*th compartment, corresponding to an “E” allele DNA molecule, were bound to a cell of the *i*th compartment, with an “M” allele at the relevant locus, the number of transformation events *T*_i,q_ was distributed as:

$$T_{i,q}\sim Bin\left(\varphi^{-1},B_{i,q}\right)$$

Correspondingly, when φ < 1, favouring the import of shorter alleles, if *B*_i,q_ complexes of a DNA molecule of the *q*th compartment, corresponding to an “M” allele DNA molecule, bound to a cell of the *i*th compartment, with an “E” allele at the relevant locus, the number of transformation events *T*_i,q_ was distributed as:

$$T_{i,q}\sim Bin\left(\varphi ,B_{i,q}\right)$$

In all other cases, *T*_i,q_ = *B*_i,q_. The default value of φ used in these simulations, 0.1, is a conservative estimate assuming a geometric distribution of imported DNA lengths parameterised according to the typical length of shorter classes of MGEs (~15 kb) [57] and an estimate of the transformation length distribution biased away from shorter transformation events (mean length of ~6.6 kb) [4,60].

The transformation rate also varied through the simulations in cases where the “C state” was included (S15 Fig), representing the regulated, transient competence state observed in many bacterial species. The trigger for entering C state was the “C signal,” the levels of which (*S*) were determined by the rate of production by all *k* compartments of cells at a rate of *e*_C_ per cell (set to 10 *t* ^-1^ per cell; light green arrows in S15 Fig), and elimination at the extracellular washout rate, ω (grey arrow in S15 Fig):

$$S_{t+1}\sim S_{t}+Pois\left(e_{C}\sum_{i=1}^{i=k}N_{i,t}.dt\right)-Bin\left(\omega .dt,S_{t}\right)$$

When the C signal surpassed the threshold *t*_C_ (set to 10^7^), the number of non-C-state cells (*N*) of compartment *i* entering C state (*C*_i_^entry^) was distributed according to a rate *g*_C_ (set to 10 *t* ^-1^ unless stated; red arrow in S15 Fig):

$$C_{i}^{entry}\sim Bin\left(g_{c}.dt,N_{i}\right)$$

Independently of the C signal, cells of compartment *i* in C state (*C*_i_) exited C state at a rate *r*_C_ (set between 0 and 0.9 *t* ^-1^, depending on the growth pattern):

$$C_{i}^{exit}\sim Bin\left(r_{C}.dt,C_{i}\right)$$

Cells in the C state grew at a reduced rate γ(1-*c*_C_), in which *c*_C_ was the cost associated with the C state. In oscillatory growth patterns, C-state cells were subject to growth arrest (*c*_C_ = 1), as has been observed in some species in which competence is regulated [37,38]. C-state cells underwent transformation at a higher rate (τ_C_) than cells not in C state (orange arrows in S15 Fig), and drove cell–cell killing of cells not in the “C state” as a mass action process at a rate *k*_C_ (dashed lines in S15 Fig); this mirrors “fratricide” in some streptococcal species or “cannabilism” in *B*. *subtilis* [15]. For the *k* cell compartments in the model, the number of non-C-state cells (*N*_i_) in the *i*th compartment killed by this mechanism (*K*_i_) depended on the total population of C-state cells across all compartments (*C*_j_), as well as *k*_C_:

$$K_{i}\sim Bin\left(k_{C}\sum_{j=1}^{j=k}C_{j}.dt,N_{i}\right)$$

Therefore, the overall change in the population of compartment *i*, if such cells were not in the C state, could be summarised as:

$$N_{i,t+1}=N_{i,t}+P_{i,t}-K_{i,t}-A_{i,t}-C_{i,t}^{entry}+C_{i,t}^{exit}-T_{i\to !i,t}+T_{!i\to i,t}-M_{i\to !i,t}+M_{!i\to i,t}$$

where *P*_i_ is the demographic change resulting from cell growth and death; *K*_i_ is the reduction as a consequence of cell-cell killing; *A*_i_ is the loss of cells due to MGE activation; *C*_i_^entry^ is the number of cells entering C state, while *C*_i_^exit^ is the number of cells of the same genotype exiting C state; *T*_i→!i_ is the consequence of transformation driving divergence to different genotypes, while *T*_!i→i_ is the number of cells being converted to compartment *i* by transformation; and *M*_i→!i_ represents MGE infection converting compartment *i* cells to other genotypes, whereas *M*_!i→i_ is the reciprocal conversion of other genotypes to compartment *i* through infection. If compartment *i* corresponds to C-state cells, the formula for the population change no longer includes cell–cell killing, and the impact of the state change terms is reversed:

$$C_{i,t+1}=C_{i,t}+P_{i,t}-A_{i,t}-C_{i,t}^{exit}+C_{i,t}^{entry}-T_{i\to !i,t}+T_{!i\to i,t}-M_{i\to !i,t}+M_{!i\to i,t}$$

For MGEs of compartment *q* (*M*_q_) interacting with *k* cell compartments, the change between timesteps can be summarised as:

$$M_{q,t+1}=M_{q,t}+R_{q,t}+E_{q,t}-d_{q}$$

where *R*_q_ represents the release of MGEs from host cells through activation, *E*_q_ is the spontaneous entry of MGEs into the model, and *d*_q_ represents MGEs lost to infection of cells and MGE washout and degradation.

For DNA of compartment *q* (*D*_q_) interacting with *k* cell compartments, the change between timesteps can be summarised as:

$$D_{q,t+1}=D_{q,t}+L_{q,t}-d_{q}$$

where *L*_q_ represents the release of DNA through cell lysis (either a consequence of cell-density-dependent death, MGE activation or cell–cell killing), and *d*_q_ represents the uptake of DNA by competent cells and DNA washout and degradation.

The model was implemented using C++, with the GNU scientific library. Each simulation was run from *t* = 0 to *t* = 1,000, with 10^3^ timesteps per unit time. Neither increasing the number of timesteps per unit time 10-fold, nor doubling the endpoint value of *t*, substantially altered the displayed results of simulations involving different MGEs and growth patterns. In some simulations involving high rates of transformation (τ ≥ 0.01), the number of timesteps per unit time resulted in detectable approximation errors in some model compartments, but validatory simulations confirmed this did not affect reported summary statistics. At *t* = 0, each genotype started at a frequency of 100 cells. If a single MGE featured in the simulation, then the two genotypes were uninfected and infected; if two MGEs featured in the simulation, then the starting cells were the two singly infected genotypes. Within each timestep, all cells were considered capable of replication at the appropriate growth rate. Molecules were first bound to cells and underwent transformation and MGE infection. Those cells remaining unbound to molecules entered and exited the C state, were eliminated through cell–cell killing, activation of some MGEs, or died through cell-density-dependent death at the appropriate per capita rates. Reported summary values were calculated over the full extent of the simulation and represent the mean of three simulations. The source code is available for download from https://github.com/nickjcroucher/mgeTransformation. Parameters are summarised, along with typical values, in S1 Table; simulation outputs are recorded in S1 Data.

### Characterising Within-Host Pneumococcal Variation

The original dataset comprised 3,085 de novo assemblies of pneumococcal isolates from the Mae La refugee camp [98]. In order to detect short-term changes in mobile genetic element content, this study identified 374 hosts associated with two or more isolates of the same multilocus sequence type [98] and considered all 1,751 genomes from bacteria carried by these individuals. A quality threshold of a draft assembly N50 greater than 10 kb and a total draft assembly length between 1.75 Mb and 2.75 Mb was imposed on this set; genomes that did not meet these criteria were reassembled with Velvet as described previously [4]. This produced a final set of 1,715 sequences from 371 hosts. CDSs within these genomes were annotated using Prodigal [141] with a model trained on the reference sequence of *S*. *pneumoniae* ATCC 700669 [142] and translated to generate a database of 3,660,212 proteins. Using BLASTP [143] with an E-value threshold of 10^−10^, this database was searched with a single representative sequence from each of the 355 clusters of orthologous proteins previously found to be specific for ICEs [57]. This process was repeated using 590 proteins specific for prophages, 142 proteins specific for phage-related chromosomal islands, and three proteins specific for a particular prophage remnant [57]. Putative variation in MGE content was inferred where two isolates of the same sequence type varied by at least five BLASTP matches to proteins characteristic of a single type of MGE; this identified 281 hosts with candidate short-term accessory genome variation. The original Illumina sequence reads were then mapped against the variable protein coding sequences using BWA [144]. This allowed the many cases likely representing the variable results of de novo assembly to be distinguished from genuine cases of MGE acquisition or loss. Of the genuine cases, almost all corresponded to changes in prophage content.

In order to determine whether such changes were likely to have occurred within a single carriage episode, it was necessary to construct a phylogeny to determine the level of relatedness between isolates from the same host. This required focusing on lineages commonly identified within the dataset; based on the candidate instances of within-host MGE variation, BCs 1-19F and 4-6B were selected (S3 Table), along with one particular case in BC14. A reference genome assembly was needed for each BC being analysed; *S*. *pneumoniae* Taiwan^19F^-14 was appropriate for BC1-19F [145], whereas novel references were required for the other two clusters. These were constructed by combining the original Velvet assemblies [98] with SGA assemblies [146] using Zorro [147], then ordering the contigs using ACT [148], as described previously [138]. For all cases in which at least two isolates of the relevant BAPS cluster had been isolated from a single host, the Illumina reads were then mapped against the reference sequence using SMALT [149] as described previously [138]. The resulting whole genome alignment was then analysed using Gubbins [99] to generate a maximum likelihood phylogeny while accounting for the frequent transformation events occurring in pneumococcal lineages. These analyses each corresponded well with the isolates’ metadata, identifying closely related clusters of isolates from individual hosts that represented likely individual carriage episodes.

In the instances in which these matched probable cases of MGE variation, regions of similarity between the de novo assemblies of the relevant isolates were identified through a BLAT [150] comparison of all the contigs in each sequence, using standard settings. This comparison file was then used to inspect the assemblies using ACT [148]. The originally identified MGE-associated sequences within the assemblies were then located, and, if part of a larger insertion that had characteristics of an MGE, the element was manually annotated. In some cases, genomes had to be reassembled as described for the references, then organised into scaffolds with SSPACE2 [151] as described previously [138], in order to extract the relevant MGE sequence. This also allowed their insertion sites to be ascertained and classified as described previously [57]. These annotated MGEs have been submitted to Genbank with the accession codes listed in S2 Table. To ensure they represented genuine pneumococcal prophages, they were included in a hierarchical clustering of known pneumococcal prophages, constructed based on CDS content as described previously [134]. Sequence reads were then mapped against these prophages using BWA with a requirement for absolute sequence identity for alignment, to minimise mapping of sequence reads originating from other prophages not in the reference set. These read alignments were then used to generate the heatmaps shown in Figs 6, 8, S5, S6 and S8.

### Distribution of Transformation and MGEs between Streptococcal Species

The 143 complete or high-quality draft streptococcal genomes listed in S4 Table were scanned for prophages using Phage_Finder v2.1 [152]. Naturally transformable species within the streptococcal genus were identified based on past reviews and recent experimental work [28,32,84,121–123]. The comparison of these raw data found naturally transformable isolates to have significantly fewer prophages than nontransformable isolates (mean number of prophages per transformable genome: 0.74; mean number of prophages per nontransformable genome: 1.77; Wilcoxon rank sum test: W = 3316, *p* = 0.00089). To ensure this result was not a consequence of biased sampling, three genomes from unnamed species were excluded (*Streptococcus* sp. I and *Streptococcus* sp. VT), as their competence for transformation could not be established. Pneumococcal isolates that represented duplicate samples of very closely related genotypes were also removed (*S*. *pneumoniae* R6, a duplicate of D39 [153], and *S*. *pneumoniae* 03–4156, 03–4183, 99–4038, and 99–4039, which all share a prophage insertion with the closely related isolate OXC141 [134]). The results described in the Discussion confirm that the observed association persists in this curated dataset.

## Supporting Information

S1 Data Summarised values output from simulations used to generate heatmaps in Figs 1, 3, 4, 5, 7, 10, S1–S3 and S13. (XLSX)

S1 Fig The effects of changing noncellular component washout rates and cell growth rates on the spread of MGEs between constitutively competent cells. (**A**) This heatmap is displayed as in Fig 3B, but with the rate at which noncellular components are washed out reduced by an order of magnitude to ω = 0.06. (**B**) This heatmap is displayed as in Fig 3B, but with the rate at which noncellular components are washed out increased to ω = 0.99. (**C**) This heatmap is displayed as in Fig 3B, but with the cell growth rate γ halved to 0.1. (**D**) This heatmap is displayed as in Fig 3B, but with the cell growth rate γ doubled to 0.4. Raw data are tabulated in S1 Data.
(PDF)

S2 Fig Effects of the frequency and amplitude of cell population oscillations on MGE transmission. (**A**) This heatmap summarises simulations in which the amplitude (*k*_C_) and frequency (*r*_C_) of cell population oscillations was varied. The colour of the cells represents the proportion of the cell population infected with MGEs over the course of the simulations. Each cell is split into two components based on the speed with which the strain entered the C state (*g*_C_ = 1 or 10). In this panel, the MGE present was MV (β = 10^−3^), and transformation was parameterised as τ = 10^−4^ and φ = 0.5. (**B**) This heatmap is displayed as in panel A, but the MGE present was MV (β = 10^−1^) and transformation was parameterised as τ = 10^−3^ and φ = 0.5. (**C**) This heatmap is displayed as in panel A, but the MGE present was MH (β = 5x10^-7^) and transformation was parameterised as τ = 10^−6^ and φ = 10^−1^. (**D**) This heatmap is displayed as in panel A, but the MGE present was MH (β = 10^−6^) and transformation was parameterised as τ = 10^−3^ and φ = 10^−1^. Raw data are tabulated in S1 Data.
(PDF)

S3 Fig HDT between transiently competent cells. Panels A–D show the effects of changing noncellular component washout rates and cell growth rates on the transmission of MGEs between transiently competent cells. (**A**) This heatmap is displayed as in Fig 4C, but with the rate at which DNA molecules and MGEs were washed out reduced by an order of magnitude to ω = 0.06; the C signal was still washed out at ω = 0.6 to avoid changing the pattern of bacterial growth. (**B**) This heatmap is displayed as that in Fig 4C, but with the rate at which DNA molecules and MGEs were washed out increased to ω = 0.99; again, the C signal was still washed out at ω = 0.6 to avoid changing the pattern of bacterial growth. (**C**) This heatmap is displayed as in Fig 4C, but with the cell growth rate γ halved to 0.1. (**D**) This heatmap is displayed as in Fig 4C, but with the cell growth rate γ doubled to 0.4. Panels E and F show the effect of oscillatory growth on the competition between two strains entering and leaving C state in synchrony, but with only one of the strains undergoing transformation in the C state (*g*_C_ = 10 and *r*_C_ = 0.5 in both cases). HDT occurs both symmetrically (“S” columns) and asymmetrically (“A” columns). (**E**) This heatmap is displayed as in Fig 1A. It shows the outcome of simulated competition between two strains, only one of which is competent for transformation in the C state, undergoing small population oscillations owing to a C-state-associated cell–cell killing rate of *k*_C_ = 10^−6^. (**F**) This heatmap is displayed as that in Fig 1A. It shows the outcome of simulated competition between two strains, only one of which is competent for transformation in the C state, undergoing large population oscillations owing to a C-state-associated cell–cell killing rate of *k*_C_ = 10^−3^. Raw data are tabulated in S1 Data.
(PDF)

S4 Fig Phylogenetic analysis of BC1-19F isolates from longitudinally sampled hosts using Gubbins. (**A**) Maximum likelihood phylogeny of isolates based on point mutations outside of putative recombination events. Each leaf node is labelled to indicate whether the *comYC* gene, required for efficient transformation, is intact. (**B**) Annotation of the reference genome of *S*. *pneumoniae* Taiwan^19F^-14. Mobile genetic element-related sequences (the Tn*916*-type ICE, PRCIs, and Pneumococcal Pathogenicity Island 1, PPI-1) are marked, as are loci encoding major antigens (the capsule polysaccharide synthesis, *cps*, locus, as well as *pspA* and *pspC*). (**C**) Putative recombinations occurring during the evolutionary history of BC1-19F. Red blocks represent putative recombinations reconstructed as occurring on an internal branch, which are, therefore, shared by multiple isolates through common descent. Blue blocks represent putative recombinations reconstructed as occurring on a terminal branch, and are, therefore, unique to a single isolate.
(PDF)

S5 Fig Distribution of prophage sequences within BC1-19F. (**A**) Maximum likelihood phylogeny generated by Gubbins, as displayed in S4 Fig. (**B**) Hierarchical clustering of prophages identified within BC1-19F and BC4-6B with previously identified pneumococcal prophages, based on CDS content. Tips with dashed lines represent those prophages identified within BC1-19F. (**C**) CDS annotations of the 14 prophages extracted from representatives of BC1-19F. (**D**) Bars marking the extent of the individual prophage, coloured to represent their site of insertion within the pneumococcal chromosome. Vertical lines within these bars represent breaks between contigs. (**E**) Heatmap representing the distribution of prophage sequences across BC1-19F. Each row corresponds to an isolate in the phylogeny and is coloured blue where there is a low depth of sequence read mapping (indicating the sequence is absent from the isolate’s genome) and red where there is a high depth of sequence read mapping (indicating the sequence is present in the isolate’s genome). Due to sequence similarity between prophages, there is extensive crossmapping between related MGEs. Each case of *comYC* disruption can be associated with the insertion of a prophage into the gene.
(PDF)

S6 Fig Apparent removal of an MGE through an interstrain transformation event. (**A**) Maximum likelihood phylogeny of BC14 representatives isolated from longitudinally sampled hosts based on point mutations outside of putative recombination events. Each leaf node is labelled to indicate whether the *comYC* gene is intact. Seven transformable closely related isolates from host ARI-0248 are annotated. (**B**) Distribution of the putative PRCI PRCI_ARI-0248_ between the seven isolates from host ARI-0248, arranged by date of isolation. Each row beneath the PRCI annotation is a heatmap showing the depth of read coverage across the MGE sequence. This indicates the PRCI is absent from two isolates, 09B10533 and 09B13198. (**D**) Alignment of a putative PRCI from *S*. *pneumoniae* TIGR4 with the draft reference genome of *S*. *pneumoniae* 10B00189, which carries PRCI_ARI-0248_, and is, in turn, aligned with the draft genome of *S*. *pneumoniae* 09B13198, which does not. In both draft genomes, the alternating orange and brown boxes indicate different contigs within the assemblies. Red bands link regions of sequence similarity, as calculated using BLAT; the intensity of the colour represents the extent of the similarity. The green box demarcates the extent of an interstrain transformation event, relative to the reference genome of 10B00198, shared by 09B10533 and 09B13198 (and no other isolates) based on the Gubbins analysis. The recombination spanned PRCI_ARI-0248_ and appears to have caused its deletion in these two isolates.
(PDF)

S7 Fig Phylogenetic analysis of BC4-6B isolates from longitudinally sampled hosts using Gubbins. (**A**) Maximum likelihood phylogeny of isolates based on point mutations outside of putative recombination events. Each leaf node is labelled to indicate whether the *comYC* gene, required for efficient transformation, is intact. (**B**) Annotation of the reference genome of *S*. *pneumoniae* 10B02680. Alternating orange and brown blocks represent different ordered contigs in the curated de novo draft assembly. Mobile genetic element-related sequence (the ICE, PRCIs, prophages, and PPI-1) are marked, as are loci encoding major antigens (the capsule polysaccharide synthesis, *cps*, locus, as well as *pspA* and *pspC*). (**C**) Putative recombinations occurring during the evolutionary history of BC4-6B. Red blocks represent putative recombinations reconstructed as occurring on an internal branch, which are, therefore, shared by multiple isolates through common descent. Blue blocks represent putative recombinations reconstructed as occurring on a terminal branch and are, therefore, unique to a single isolate.
(PDF)

S8 Fig Distribution of prophage sequences within BC4-6B. (**A**) Maximum likelihood phylogeny generated by Gubbins, as displayed in S7 Fig. (**B**) Hierarchical clustering of prophages identified within BC1-19F and BC4-6B with previously identified pneumococcal prophages, based on CDS content. Tips with dashed lines represent those prophages identified within BC4-6B. (**C**) CDS annotations of the twelve prophages extracted from representatives of BC4-6B. (**D**) Bars marking the extent of the prophages, coloured to represent their site of insertion within the pneumococcal chromosome. Vertical lines within these bars represent breaks between contigs. (**E**) Heatmap representing the distribution of prophage sequences across BC4-6B. Each row corresponds to an isolate in the phylogeny and is coloured blue where there is a low depth of sequence read mapping and red where there is a high depth of sequence read mapping. Due to sequence similarity between prophages, there is extensive crossmapping between related MGEs. Each case of *comYC* disruption can be associated with the insertion of a prophage into the gene.
(PDF)

S9 Fig Prophages with integrases similar to that found in the prophage disrupting *comYC* in *S*. *pneumoniae* 670-6B (SP670_2190). (**A**) Comparison of *Streptococcus mutans* isolates UA159 and NLML9, the latter of which has a prophage inserted into the *comYC* gene encoding the major structural component of the competence pilus. The accession codes of each sequence are given in brackets underneath the isolate names. Blue and orange boxes represent cellular CDSs, with the direction of transcription indicated by their vertical position relative to the horizontal line; pink boxes represent MGE CDSs in the same way. Brown boxes linked by dashed lines mark fragments of a pseudogene disrupted by an MGE insertion. The red bands link regions of similar sequence in the two loci, with the intensity of the colour representing the strength of the match. The level of protein identity between this prophage integrase and that disrupting the *comYC* gene of *S*. *pneumoniae* 670-6B (SP670_2190) is annotated. (**B**) Comparison of *Streptococcus parauberis* isolates KRS-02109 and KRS-02083, the latter of which has a prophage inserted into the *comYC* gene. (**C**) Comparison between *Lactococcus lactis* isolates IL1403 and KLDS 4.0325, the latter of which has a prophage inserted into the *comYC* gene. This comparison is also shown in Fig 9A. (**D**) Comparison between *Streptococcus agalactiae* isolates COH1 and FSL S3-277, the latter of which has a prophage inserted into the *cas3* gene of the *S*. *agalactiae* CRISPR2 locus. This comparison is also shown in Fig 9B.
(PDF)

S10 Fig MGE insertion sites within competence-associated genes. (**A**) Insertion of prophages into *comYC*. All prophages had an integrase similar to SP670_2190. This section of the *comYC* codon alignment shows the prophages identified in *Streptococcus parauberis*, *Streptococcus mutans*, and *Lactococcus lactis* all insert into an orthologous, but not perfectly conserved, location within the gene. (**B**) Insertion of MGEs into *comM*. All MGEs had an integrase similar to CF65_00446. This section of the *comM* codon alignment shows the MGEs identified in *Pseudomonas syringae*, *Francisella philomiragia*, *Mannheimia haemolytica*, and *Acinetobacter baumannii* all insert into an orthologous, but not perfectly conserved, location within the gene. (**C**) Insertion of prophages into *comFA*. The prophages identified in *Bacillus thuringiensis* and *Bacillus cereus* have integrases similar to LMRG_01511 (and are 80.9% identical to one another), and both insert at orthologous, but nonidentical, sites within the *comFA* codon alignment. However, the prophage inserted into *comFA* in *Streptococcus suis* has a distinct integrase (only 34.1% identity with that identified in *B*. *cereus*), and correspondingly inserts into a different site much further downstream in the codon alignment.
(PDF)

S11 Fig Prophages with integrases similar to that found in the prophage disrupting *comK* in *Listeria monocytogenes* 10403S (LMRG_01511). (**A**) Comparison of *Listeria innocua* isolates 9KSM and Clip11262, the latter of which has a prophage inserted into the *comK* gene, encoding the orthologue of the main regulator of competence in *Bacillus* *subtilis*. The comparison is displayed as described in S9 Fig. (**B**) Comparison of *Bacillus cereus* isolates MHI 226 and VD214, the latter of which has a prophage inserted into the *comFA* gene at a site distinct from that targeted by the prophage displayed in Fig 9C. This comparison is also shown in Fig 9D. (**C**) Comparison of *Bacillus thuringiensis* isolate BMB171 and a representative of serovar tolworthi, the latter of which has a prophage inserted into the *comFA* gene. (**D**) Comparison of *Enterococcus faecalis* isolates V583 and RMC65, the latter of which has a prophage inserted into the *radC* gene, often upregulated during competence in multiple species.
(PDF)

S12 Fig MGEs with integrases similar to that found in the MGE disrupting *comM* in *Aggregatibacter actinomycetemcomitans* HK1651 (CF65_00446). (**A**) Comparison of *Acinetobacter baumannii* isolates LAC-4 and 1598530, the latter of which has an MGE inserted into a CDS encoding an orthologue of ComM, a protein identified as increasing transformation efficiency in *H*. *influenzae*. The comparison is displayed as described in S9 Fig. (**B**) Comparison of *Mannheimia haemolytica* isolates D171 and USMARC-185, the latter of which has an MGE inserted into a CDS encoding an orthologue of ComM. (**C**) Comparison of *Francisella philomiragia* isolates ATCC 25015 and FAJ, the latter of which has an MGE inserted into a CDS encoding an orthologue of ComM. (**D**) Comparison of *Pseudomonas syringae* isolates UMAF0158 and BRIP34881, the latter of which has an MGE inserted into a CDS encoding an orthologue of ComM.
(PDF)

S13 Fig Exploring parameter variation and model limitations relating to interactions between MGEs and cells. Panels A–D show further simulations investigating MGE strategies for reducing elimination by transformation. A particular issue with the simulations presented in Fig 10B and 10C was that the high value of *f* was especially detrimental to an MGE in early timesteps, when cells are at a low density; these simulations test for the success of different strategies when MGEs are able to invade a cell population after it had reached its carrying capacity. (**A**) Heatmap showing the same simulations as in Fig 10A, except that bursts of MGEs were introduced at a rate of 10^−3^ *t* ^-1^ rather than being polymorphic in the initial population. The colours of the cells represent the proportion of the cell population infected by MGEs over the duration of the simulations. (**B**) Heatmap showing the overall cell population through the simulations shown in panel A on a log_10_ scale. (**C**) Heatmap showing the same simulations as in Fig 10B, except that bursts of MGEs were introduced at a rate of 10^−3^ *t* ^-1^ rather than being polymorphic in the initial population. (**D**) Heatmap showing the overall cell population through the simulations shown in panel C on a log_10_ scale. Panels E and F evaluate the impact of artefactual antagonism between MGE infection and transformation. The model was altered such that whenever cells bound both DNA and MGEs, MGE infection occurred preferentially in place of transformation. (**E**) The set of simulations displayed in Fig 3B are repeated with the altered model. (**F**) The set of simulations displayed in Fig 4C are repeated with the altered model. Raw data are tabulated in S1 Data.
(PDF)

S14 Fig Distribution of prophages in complete or high-quality draft streptococcal genomes. The genomes listed in S4 Table are plotted in terms of their overall size and the number of prophages detected within them. Points are coloured red if the isolate was known to be naturally transformable, or otherwise blue.
(PDF)

S15 Fig Structure of the stochastic compartmental model. (**A**) Links between cellular, DNA, and MGE compartments in the basic model. Each compartment type is represented by a different colour; the cell genotype can change through either interaction with a DNA compartment (transformation) or an MGE compartment (MGE infection). Not shown are genetically “silent” transformation and infection events that deplete noncellular compartment populations but do not affect cell genotypes. Cells replicate according to their growth rate, as modified by the cost of carried MGEs, and die through density-dependent cell death and activation of some MGEs. Density-dependent cell death releases one DNA molecule of the allele present at each locus of the genotype; cell deaths associated with MGE activation release a burst of MGEs, and one molecule of the allele present at the nonactivating locus. (**B**) Incorporation of transient competence into the model. All cells generate a C signal, and above a threshold level, this signal drives cells to enter the C state. Cells left C state at a constant per capita rate, independent of the level of C signal. Genetic alterations through transformation were only possible when cells were in the C state. The C state also affected the population dynamics, as, in some simulations, the replication of cells was transiently arrested while they were in C state (if *c*_C_ = 1 in the “bet hedging” and oscillatory growth patterns), and C-state cells also inhibited the growth of non-C-state cells through cell–cell killing (if *k*_C_ > 0 in oscillatory growth patterns).
(PDF)

S1 Table Description of model parameters with typical values. (DOCX)

S2 Table Properties and accession codes of prophages identified as part of this work. The annotated prophage sequences shown in S5 and S8 Figs have been deposited in Genbank with the listed accession codes. The insertion sites of the prophages, described as in [57], are detailed along with the properties of the host bacterium.
(DOCX)

S3 Table Epidemiological information association with the sequence data displayed in Figs 6 and 8. (XLSX)

S4 Table Distribution of prophages in streptococcal genomes. This table displays the properties of annotated streptococcal genomes, whether the isolate is known to be naturally transformable, and the summarized output of the Phage_Finder algorithm when applied to this sequence. These data were used to test for any difference in the distribution of prophages between isolates known to be naturally transformable and those that are not.
(DOCX)

## Abbreviations

BC: BAPS cluster
BLAT: BLAST-like alignment tool
CDS: coding sequence
CRISPR: clustered regularly interspaced short palindromic repeats
dsDNA: double-stranded DNA
GI: genomic island
HDT: horizontal DNA transfer
ICE: integrative and conjugative elements
I cell: infected cell
MGE: mobile genetic element
MH: more horizontal MGE
MI: intermediate MGE
MV: more vertical MGE
PRCI: phage-related chromosomal island
S cell: susceptible cell
ssDNA: single-stranded DNA
