# Supplementary figures and images for: Horizontal DNA Transfer Mechanisms of Bacteria as Weapons of Intragenomic Conflict
Source: PLoS Biol. 2016 Mar 2;14(3):e1002394. doi: 10.1371/journal.pbio.1002394 (PMC4774983; doi:10.1371/journal.pbio.1002394)

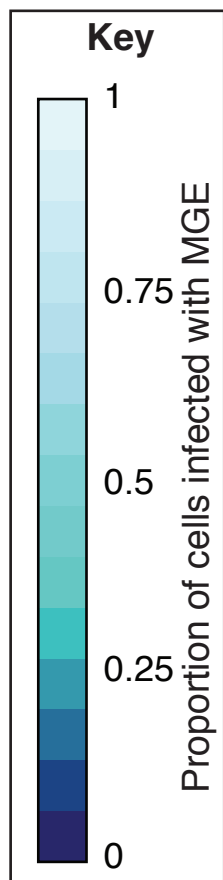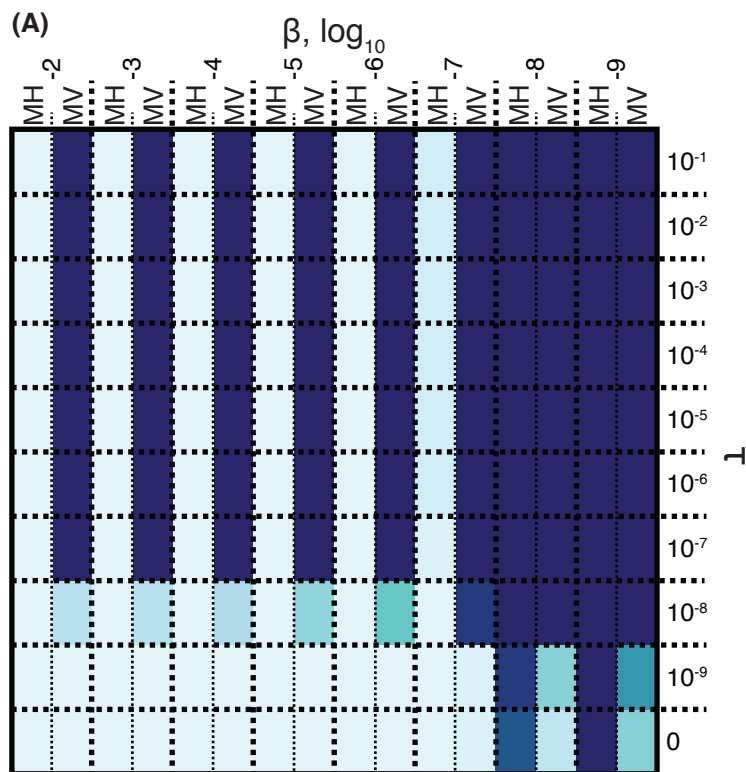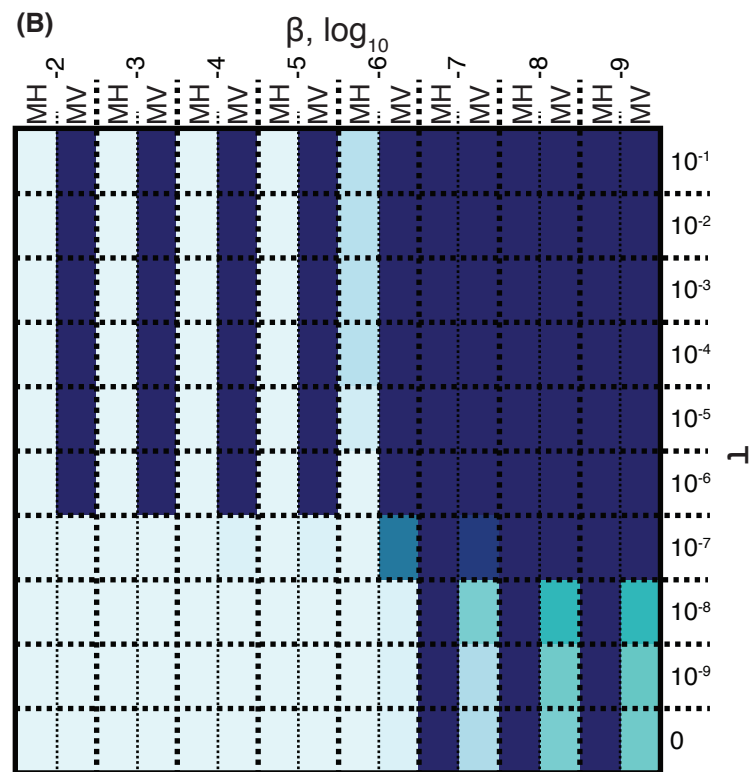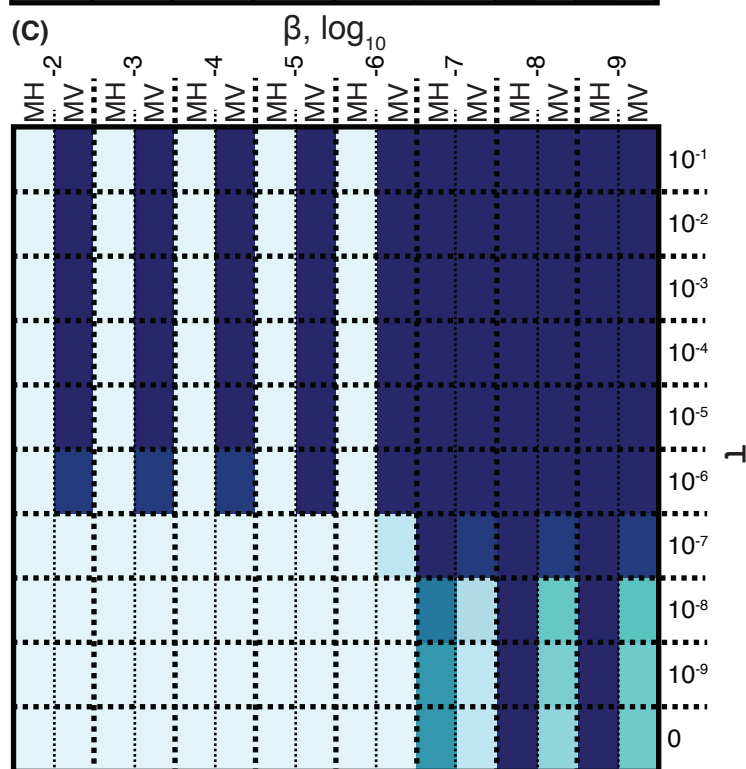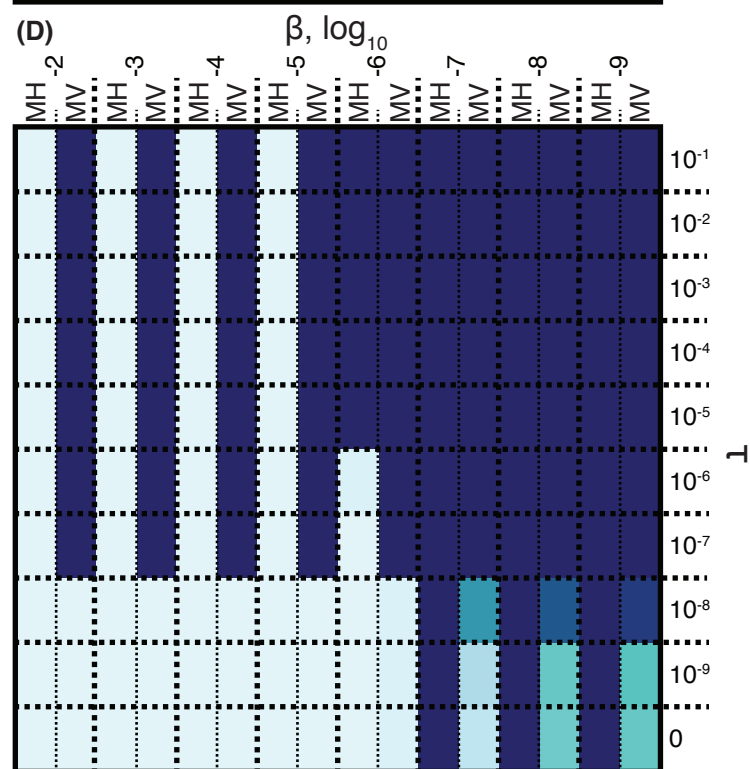

Supplement: S1 Fig — (A) This heatmap is displayed as in Fig 3B, but with the rate at which noncellular components are washed out reduced by an order of magnitude to ω = 0.06. (B) This heatmap is displayed as in Fig 3B, but with the rate at which noncellular components are washed out increased to ω = 0.99. (C) This heatmap is displayed as in Fig 3B, but with the cell growth rate γ halved to 0.1. (D) This heatmap is displayed as in Fig 3B, but with the cell growth rate γ doubled to 0.4. Raw data are tabulated in S1 Data. (PDF) [file pbio.1002394.s002.pdf]

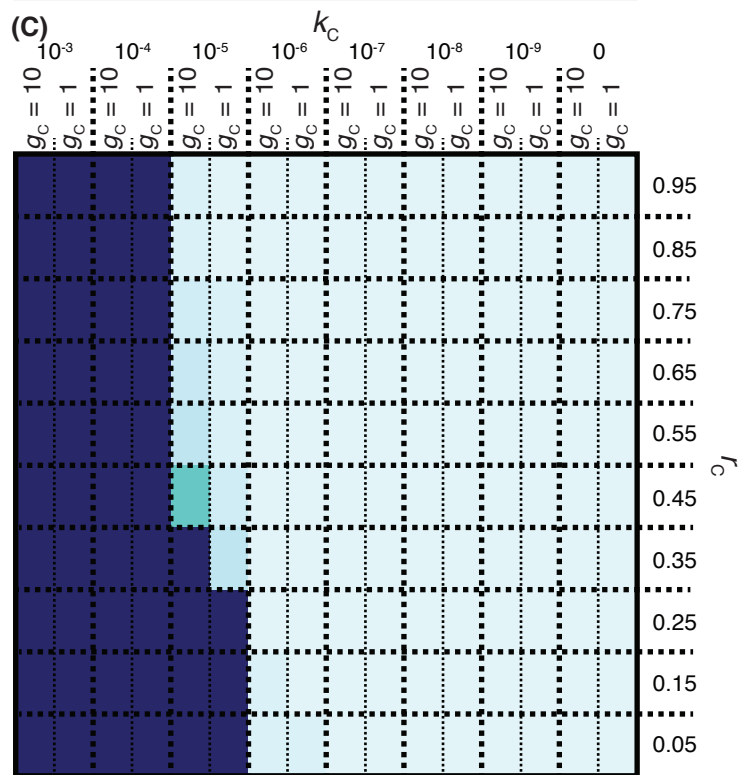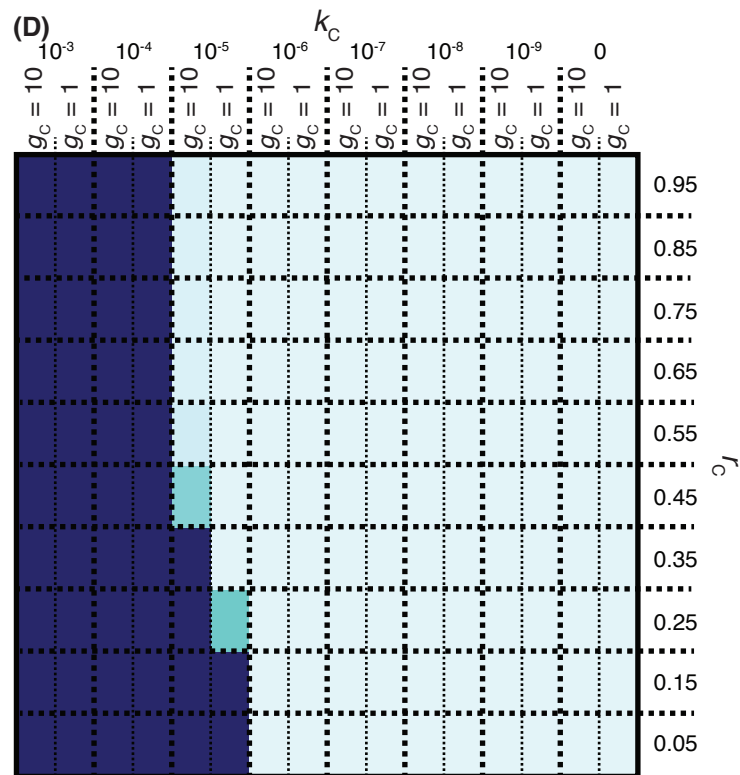

Supplement: S2 Fig — (A) This heatmap summarises simulations in which the amplitude (k C) and frequency (r C) of cell population oscillations was varied. The colour of the cells represents the proportion of the cell population infected with MGEs over the course of the simulations. Each cell is split into two components based on the speed with which the strain entered the C state (g C = 1 or 10). In this panel, the MGE present was MV (β = 10−3), and transformation was parameterised as τ = 10−4 and φ = 0.5. (B) This heatmap is displayed as in panel A, but the MGE present was MV (β = 10−1) and transformation was parameterised as τ = 10−3 and φ = 0.5. (C) This heatmap is displayed as in panel A, but the MGE present was MH (β = 5x10^-7^) and transformation was parameterised as τ = 10−6 and φ = 10−1. (D) This heatmap is displayed as in panel A, but the MGE present was MH (β = 10−6) and transformation was parameterised as τ = 10−3 and φ = 10−1. Raw data are tabulated in S1 Data. (PDF) [file pbio.1002394.s003.pdf]

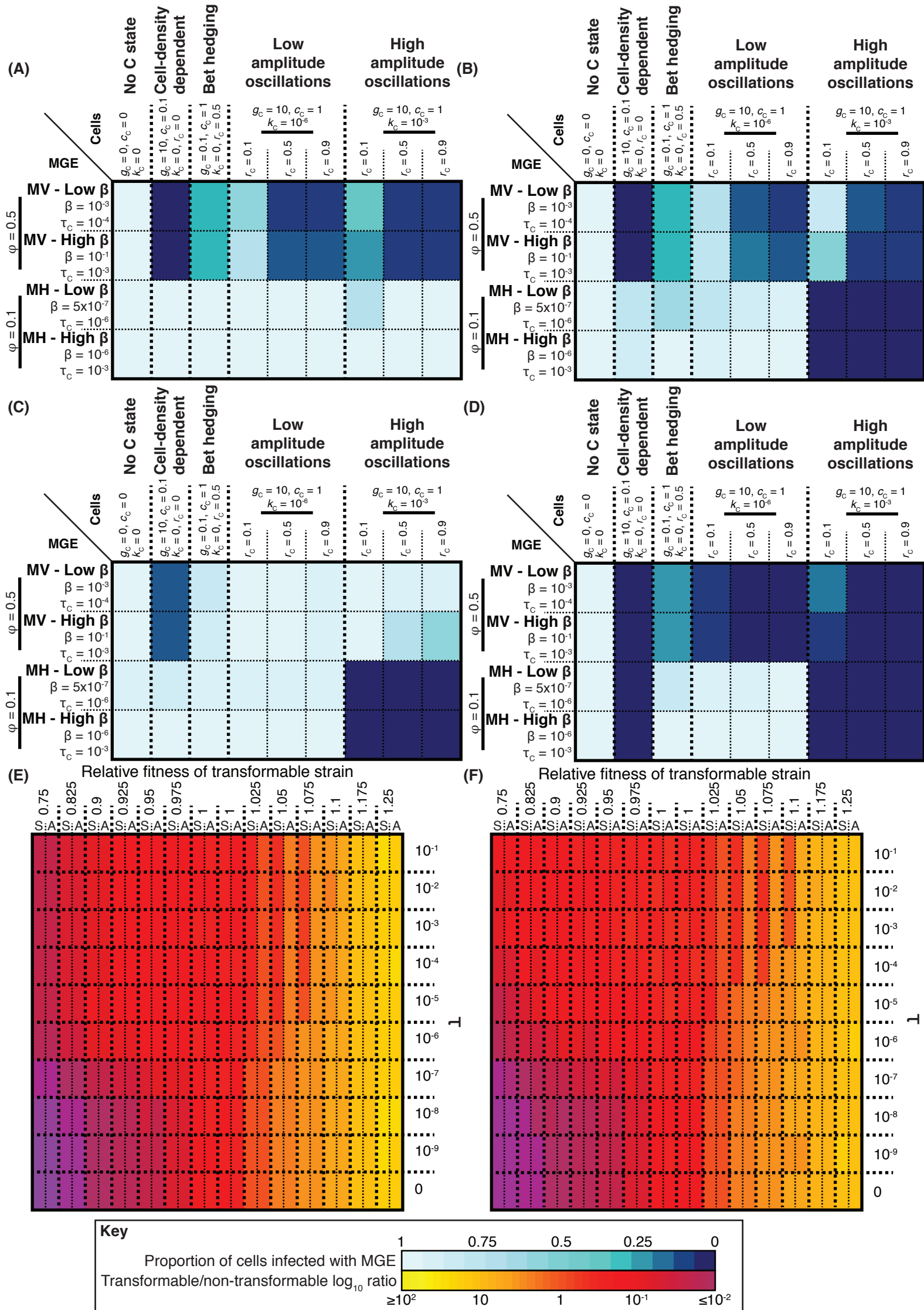

Supplement: S3 Fig — Panels A–D show the effects of changing noncellular component washout rates and cell growth rates on the transmission of MGEs between transiently competent cells. (A) This heatmap is displayed as in Fig 4C, but with the rate at which DNA molecules and MGEs were washed out reduced by an order of magnitude to ω = 0.06; the C signal was still washed out at ω = 0.6 to avoid changing the pattern of bacterial growth. (B) This heatmap is displayed as that in Fig 4C, but with the rate at which DNA molecules and MGEs were washed out increased to ω = 0.99; again, the C signal was still washed out at ω = 0.6 to avoid changing the pattern of bacterial growth. (C) This heatmap is displayed as in Fig 4C, but with the cell growth rate γ halved to 0.1. (D) This heatmap is displayed as in Fig 4C, but with the cell growth rate γ doubled to 0.4. Panels E and F show the effect of oscillatory growth on the competition between two strains entering and leaving C state in synchrony, but with only one of the strains undergoing transformation in the C state (g C = 10 and r C = 0.5 in both cases). HDT occurs both symmetrically (“S” columns) and asymmetrically (“A” columns). (E) This heatmap is displayed as in Fig 1A. It shows the outcome of simulated competition between two strains, only one of which is competent for transformation in the C state, undergoing small population oscillations owing to a C-state-associated cell–cell killing rate of k C = 10−6. (F) This heatmap is displayed as that in Fig 1A. It shows the outcome of simulated competition between two strains, only one of which is competent for transformation in the C state, undergoing large population oscillations owing to a C-state-associated cell–cell killing rate of k C = 10−3. Raw data are tabulated in S1 Data. (PDF) [file pbio.1002394.s004.pdf]

**Leaf node label key**  
— Intact *comYC* gene  
— Disrupted *comYC* gene

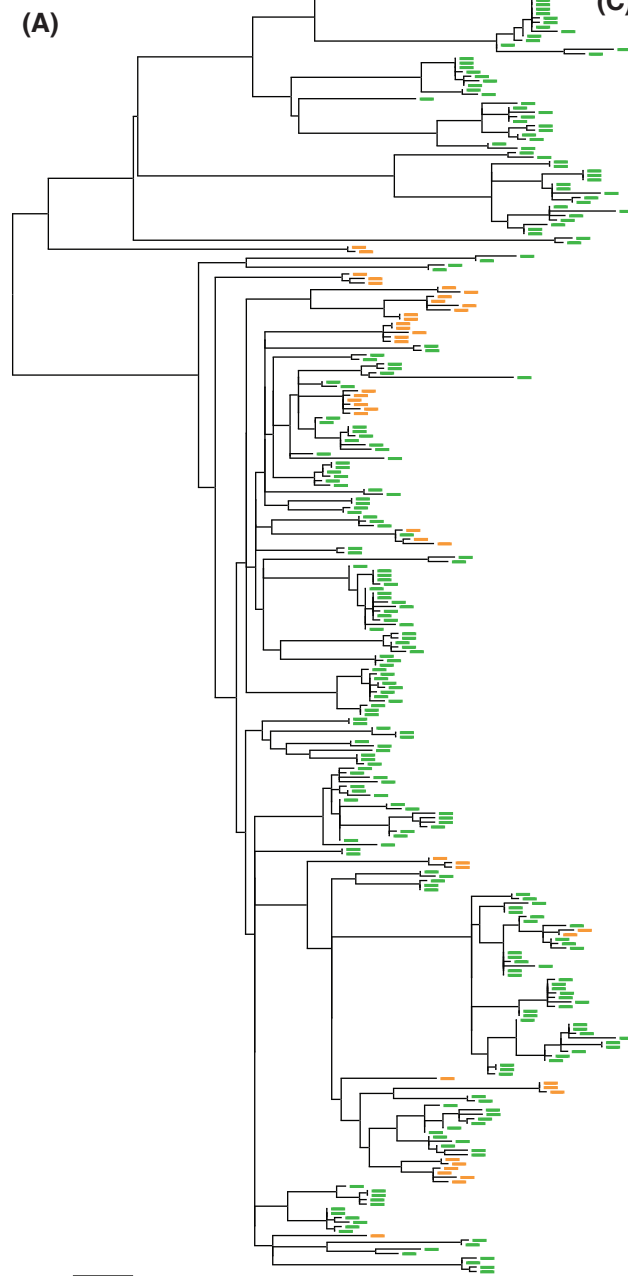

7.9 base substitutions

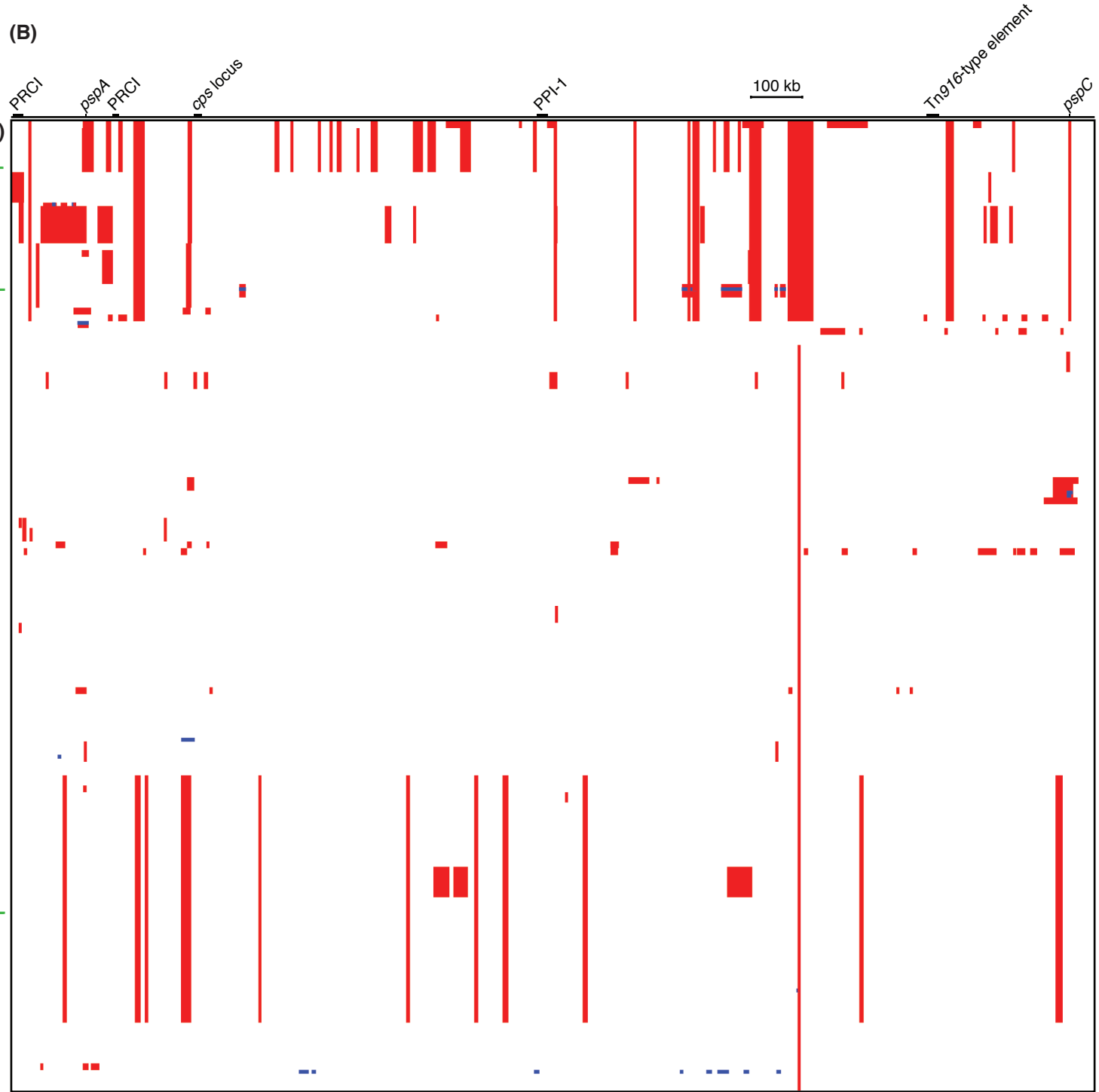

Supplement: S4 Fig — (A) Maximum likelihood phylogeny of isolates based on point mutations outside of putative recombination events. Each leaf node is labelled to indicate whether the comYC gene, required for efficient transformation, is intact. (B) Annotation of the reference genome of S. pneumoniae Taiwan19F-14. Mobile genetic element-related sequences (the Tn916-type ICE, PRCIs, and Pneumococcal Pathogenicity Island 1, PPI-1) are marked, as are loci encoding major antigens (the capsule polysaccharide synthesis, cps, locus, as well as pspA and pspC). (C) Putative recombinations occurring during the evolutionary history of BC1-19F. Red blocks represent putative recombinations reconstructed as occurring on an internal branch, which are, therefore, shared by multiple isolates through common descent. Blue blocks represent putative recombinations reconstructed as occurring on a terminal branch, and are, therefore, unique to a single isolate. (PDF) [file pbio.1002394.s005.pdf]

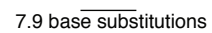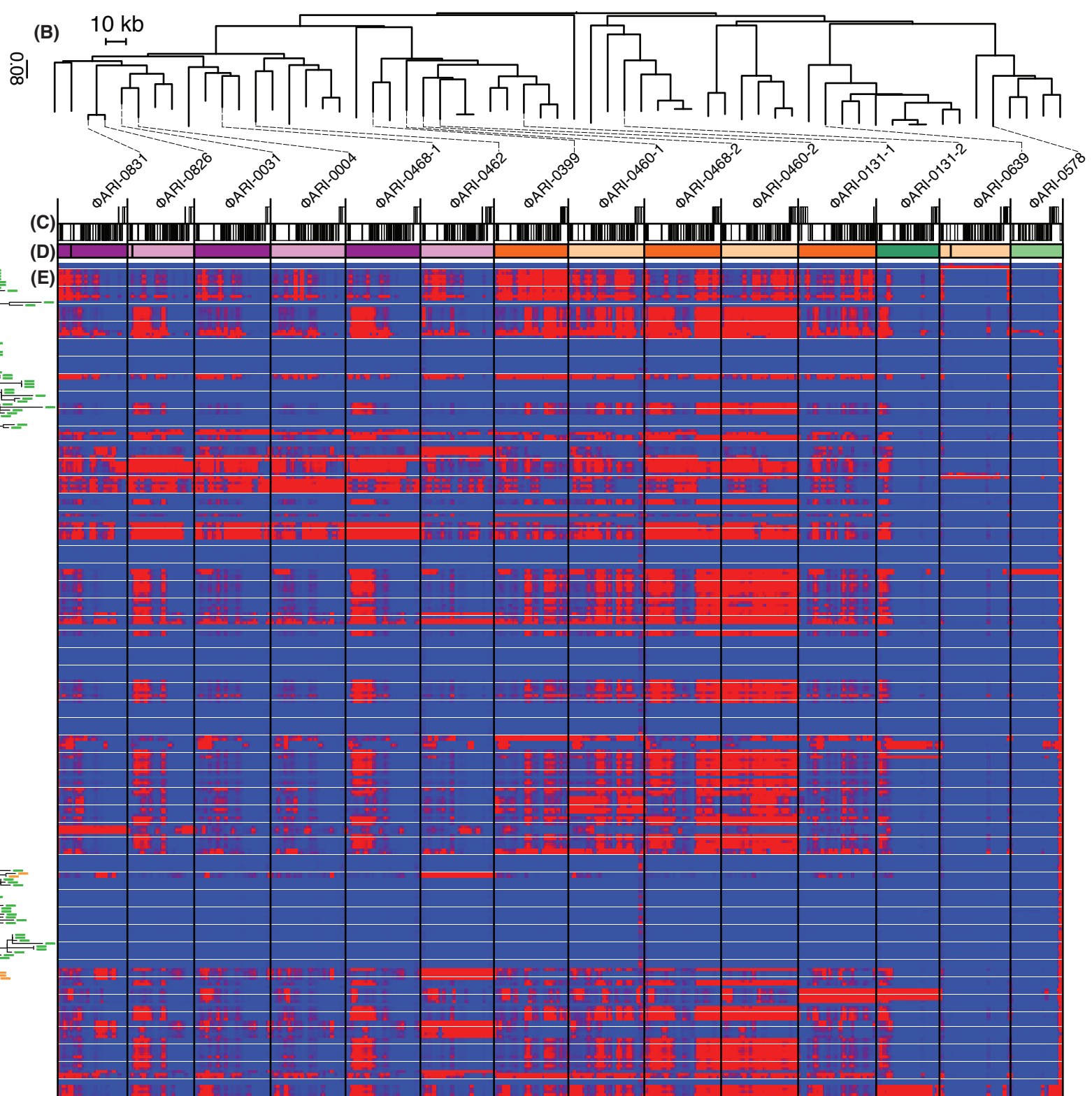

Supplement: S5 Fig — (A) Maximum likelihood phylogeny generated by Gubbins, as displayed in S4 Fig. (B) Hierarchical clustering of prophages identified within BC1-19F and BC4-6B with previously identified pneumococcal prophages, based on CDS content. Tips with dashed lines represent those prophages identified within BC1-19F. (C) CDS annotations of the 14 prophages extracted from representatives of BC1-19F. (D) Bars marking the extent of the individual prophage, coloured to represent their site of insertion within the pneumococcal chromosome. Vertical lines within these bars represent breaks between contigs. (E) Heatmap representing the distribution of prophage sequences across BC1-19F. Each row corresponds to an isolate in the phylogeny and is coloured blue where there is a low depth of sequence read mapping (indicating the sequence is absent from the isolate’s genome) and red where there is a high depth of sequence read mapping (indicating the sequence is present in the isolate’s genome). Due to sequence similarity between prophages, there is extensive crossmapping between related MGEs. Each case of comYC disruption can be associated with the insertion of a prophage into the gene. (PDF) [file pbio.1002394.s006.pdf]

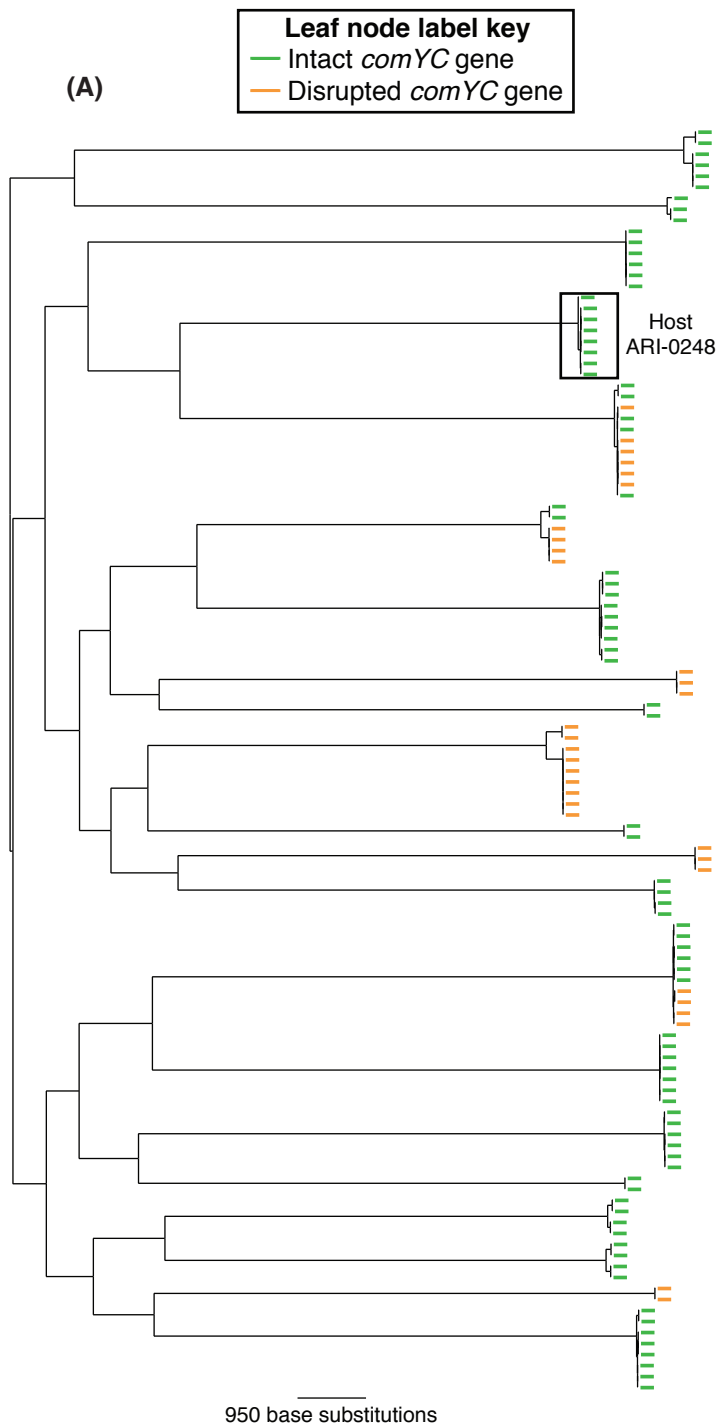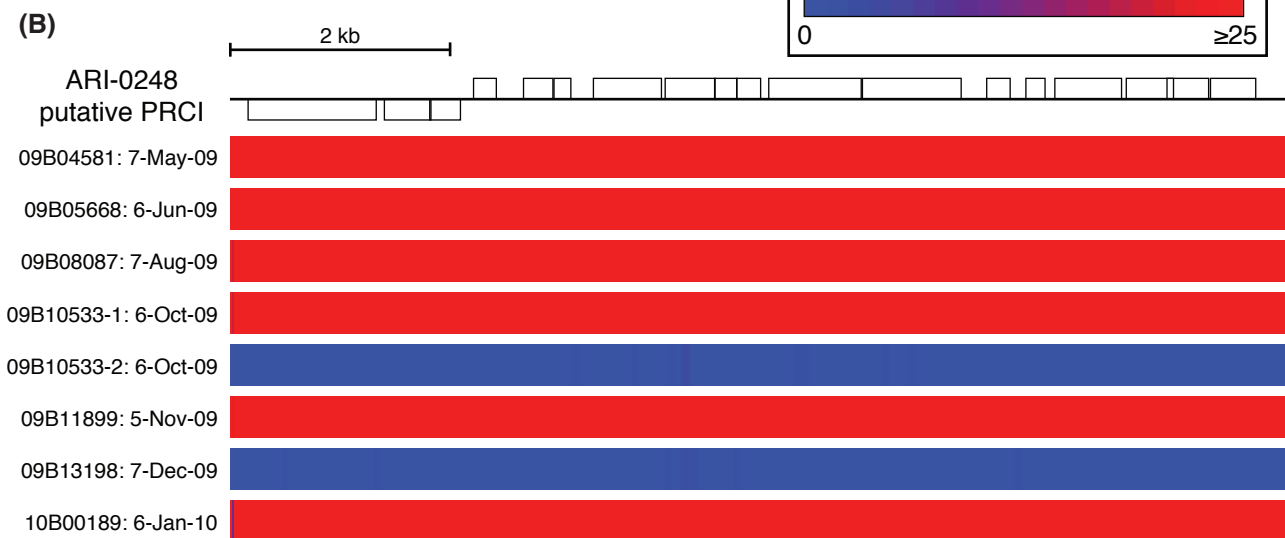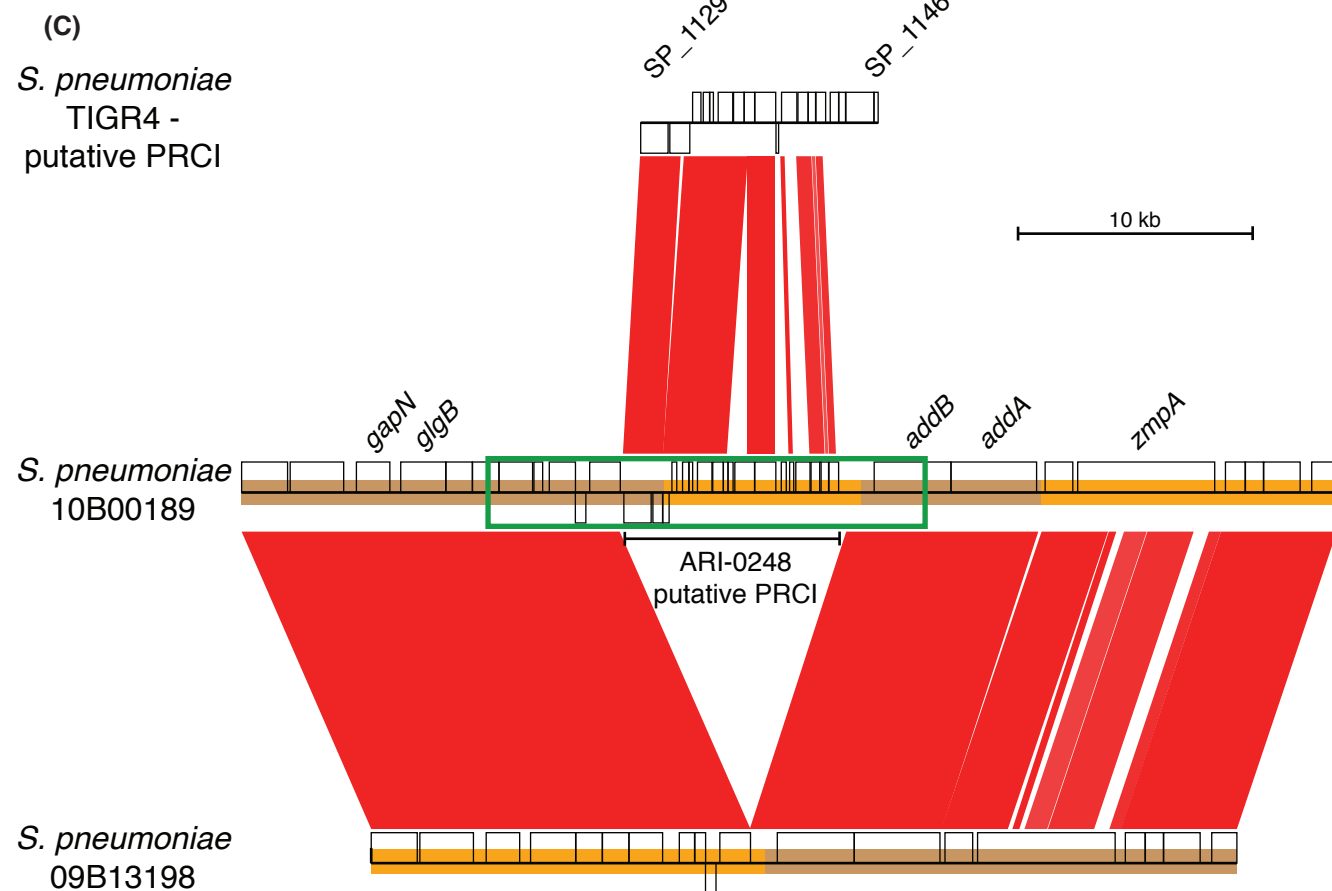

Supplement: S6 Fig — (A) Maximum likelihood phylogeny of BC14 representatives isolated from longitudinally sampled hosts based on point mutations outside of putative recombination events. Each leaf node is labelled to indicate whether the comYC gene is intact. Seven transformable closely related isolates from host ARI-0248 are annotated. (B) Distribution of the putative PRCI PRCIARI-0248 between the seven isolates from host ARI-0248, arranged by date of isolation. Each row beneath the PRCI annotation is a heatmap showing the depth of read coverage across the MGE sequence. This indicates the PRCI is absent from two isolates, 09B10533 and 09B13198. (D) Alignment of a putative PRCI from S. pneumoniae TIGR4 with the draft reference genome of S. pneumoniae 10B00189, which carries PRCIARI-0248, and is, in turn, aligned with the draft genome of S. pneumoniae 09B13198, which does not. In both draft genomes, the alternating orange and brown boxes indicate different contigs within the assemblies. Red bands link regions of sequence similarity, as calculated using BLAT; the intensity of the colour represents the extent of the similarity. The green box demarcates the extent of an interstrain transformation event, relative to the reference genome of 10B00198, shared by 09B10533 and 09B13198 (and no other isolates) based on the Gubbins analysis. The recombination spanned PRCIARI-0248 and appears to have caused its deletion in these two isolates. (PDF) [file pbio.1002394.s007.pdf]

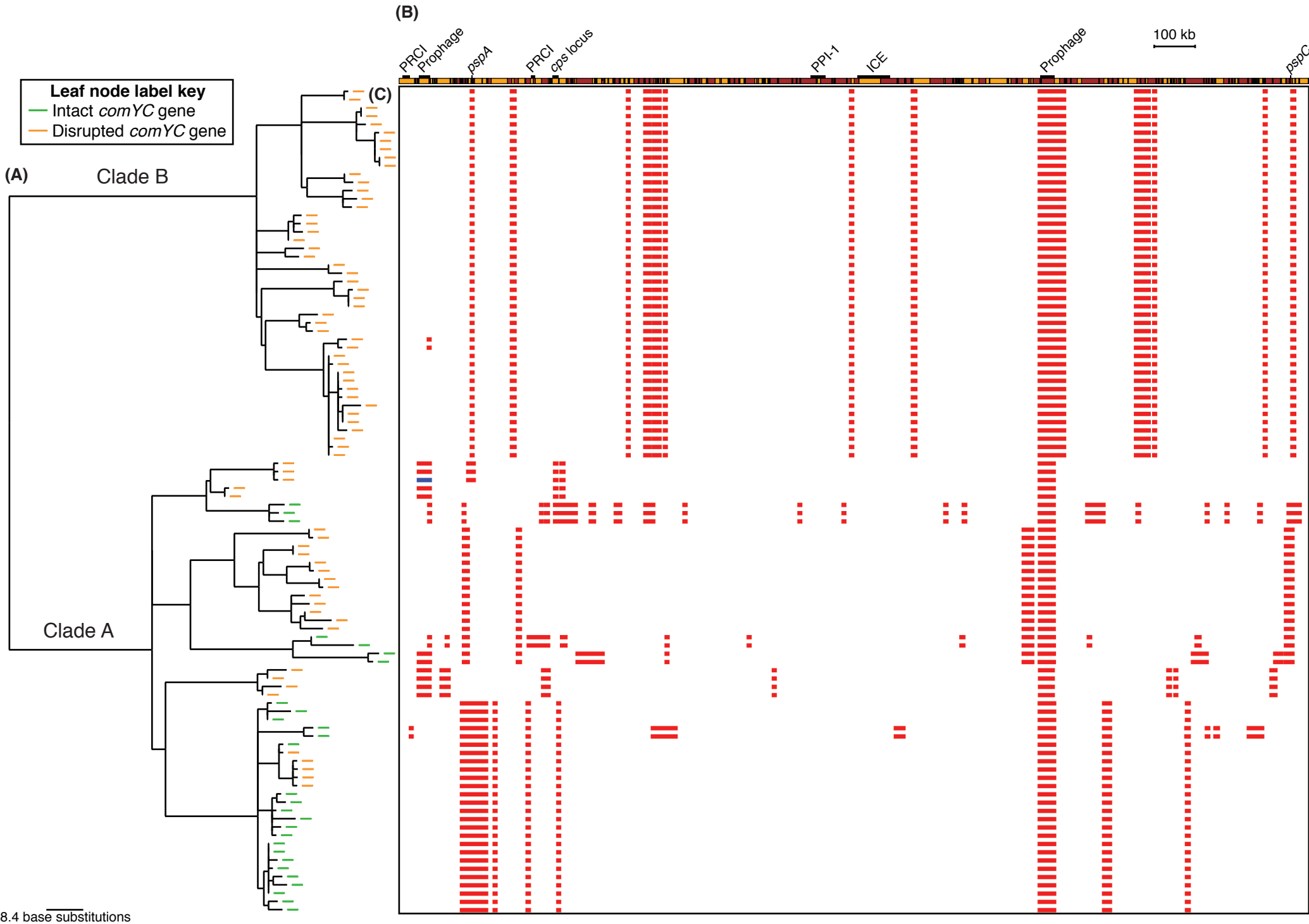

Supplement: S7 Fig — (A) Maximum likelihood phylogeny of isolates based on point mutations outside of putative recombination events. Each leaf node is labelled to indicate whether the comYC gene, required for efficient transformation, is intact. (B) Annotation of the reference genome of S. pneumoniae 10B02680. Alternating orange and brown blocks represent different ordered contigs in the curated de novo draft assembly. Mobile genetic element-related sequence (the ICE, PRCIs, prophages, and PPI-1) are marked, as are loci encoding major antigens (the capsule polysaccharide synthesis, cps, locus, as well as pspA and pspC). (C) Putative recombinations occurring during the evolutionary history of BC4-6B. Red blocks represent putative recombinations reconstructed as occurring on an internal branch, which are, therefore, shared by multiple isolates through common descent. Blue blocks represent putative recombinations reconstructed as occurring on a terminal branch and are, therefore, unique to a single isolate. (PDF) [file pbio.1002394.s008.pdf]

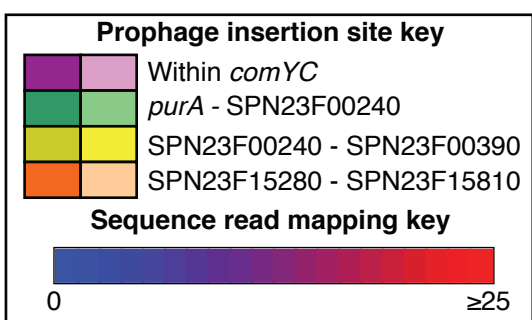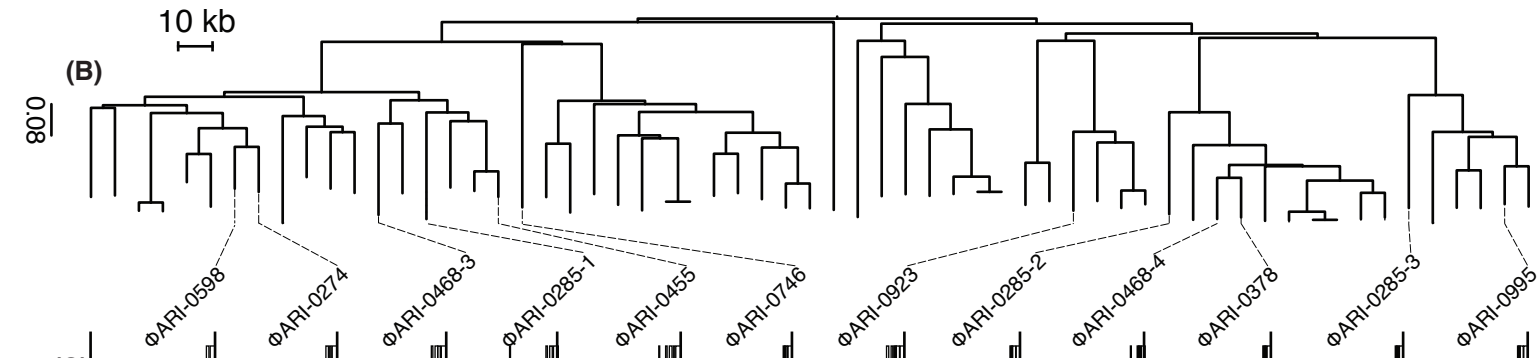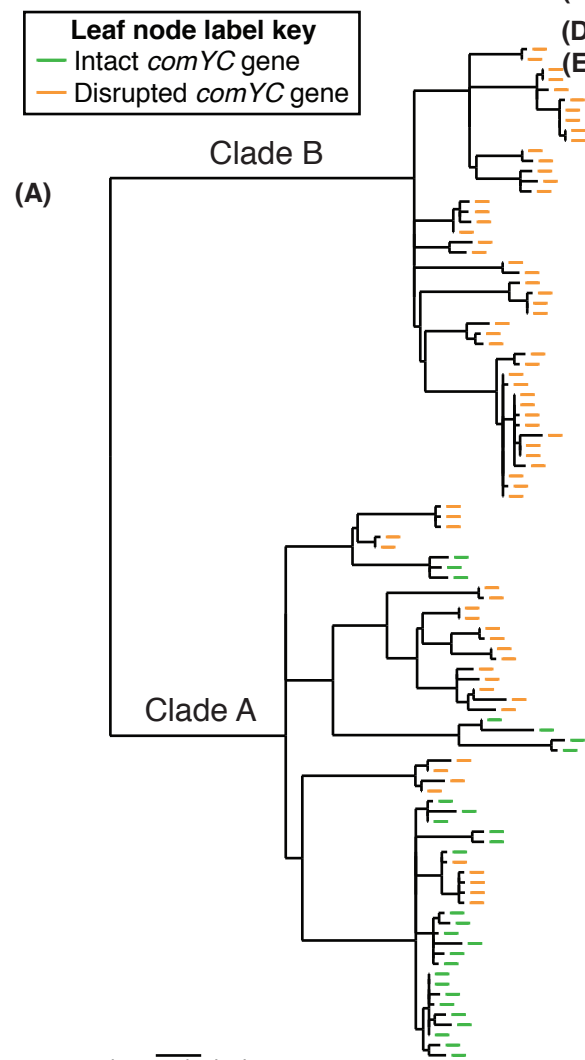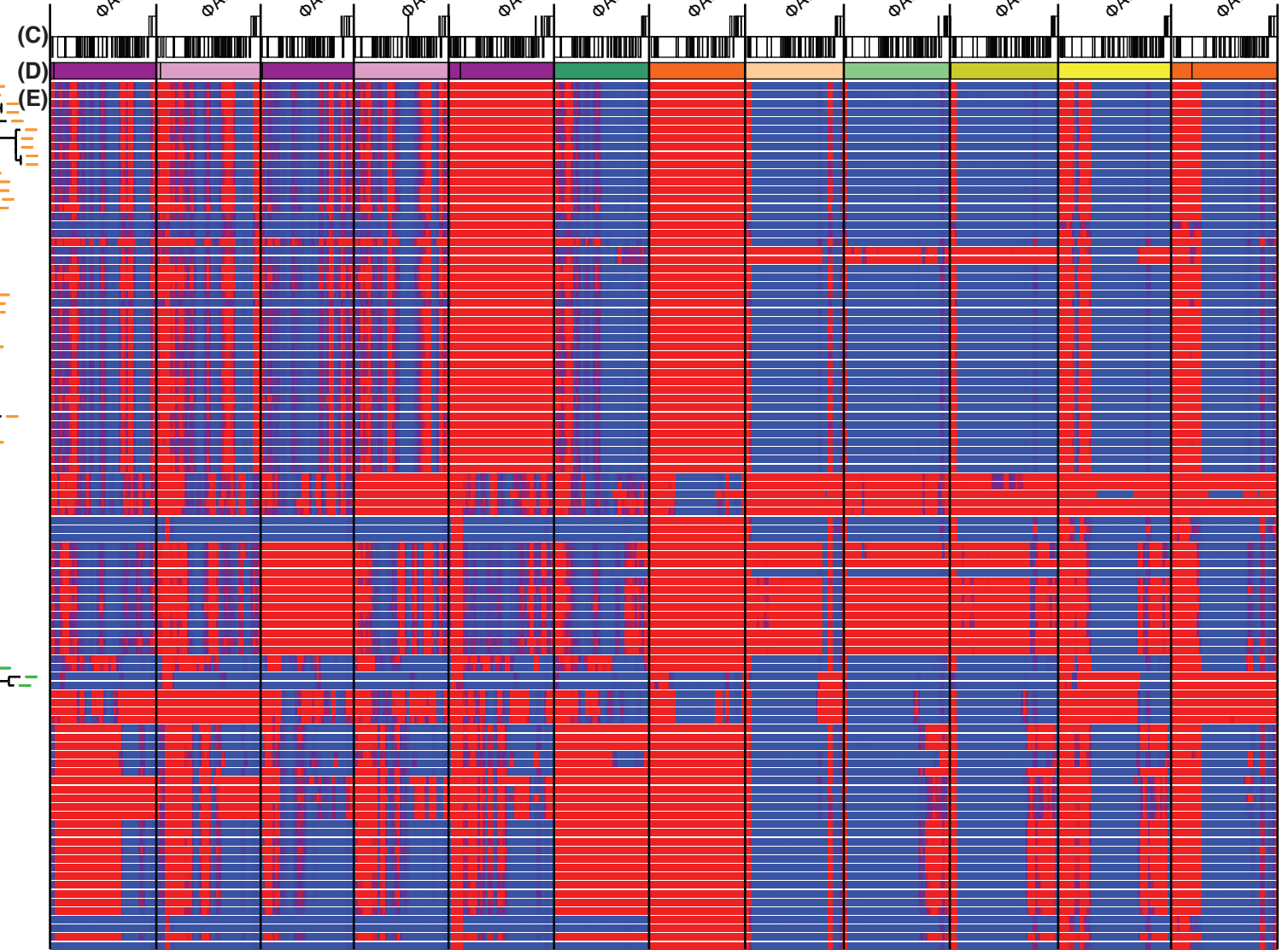

Supplement: S8 Fig — (A) Maximum likelihood phylogeny generated by Gubbins, as displayed in S7 Fig. (B) Hierarchical clustering of prophages identified within BC1-19F and BC4-6B with previously identified pneumococcal prophages, based on CDS content. Tips with dashed lines represent those prophages identified within BC4-6B. (C) CDS annotations of the twelve prophages extracted from representatives of BC4-6B. (D) Bars marking the extent of the prophages, coloured to represent their site of insertion within the pneumococcal chromosome. Vertical lines within these bars represent breaks between contigs. (E) Heatmap representing the distribution of prophage sequences across BC4-6B. Each row corresponds to an isolate in the phylogeny and is coloured blue where there is a low depth of sequence read mapping and red where there is a high depth of sequence read mapping. Due to sequence similarity between prophages, there is extensive crossmapping between related MGEs. Each case of comYC disruption can be associated with the insertion of a prophage into the gene. (PDF) [file pbio.1002394.s009.pdf]

(A)

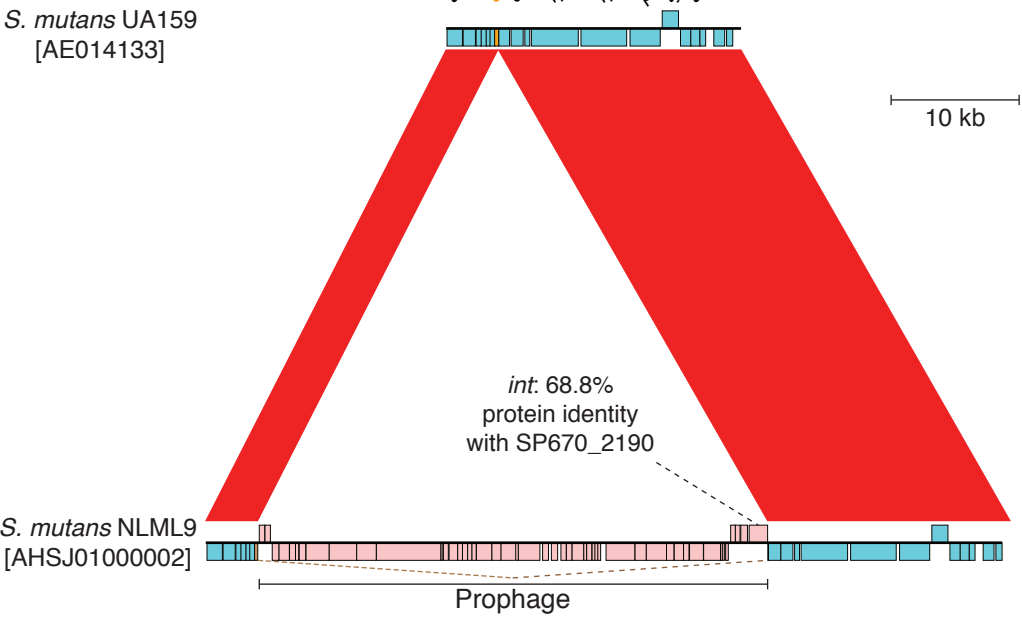

(B)

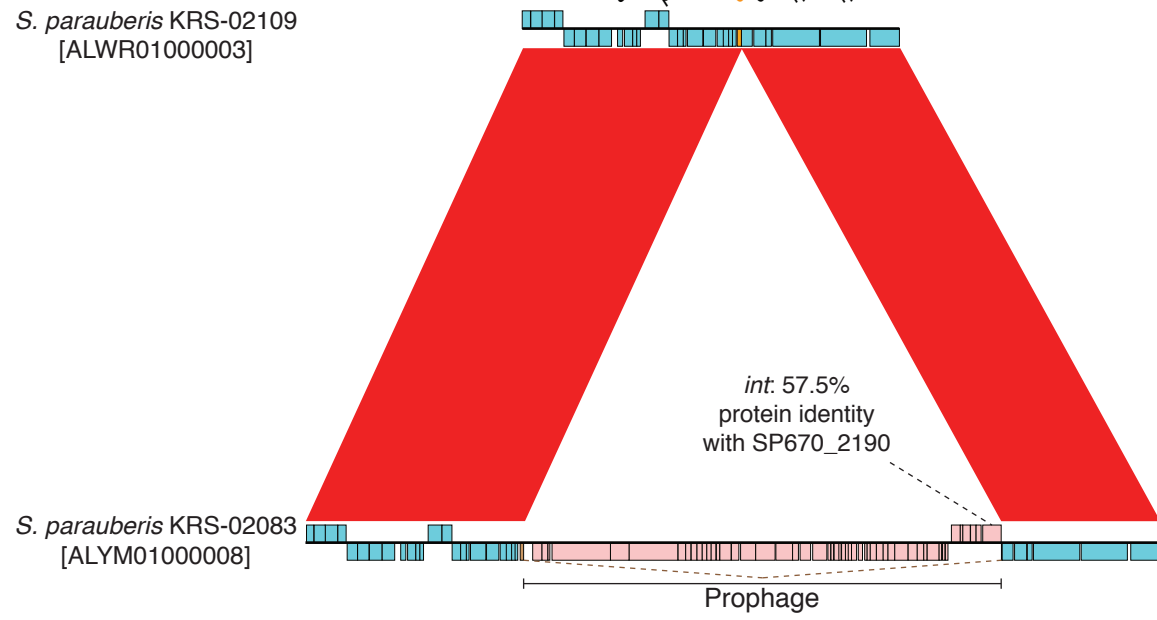

(C)

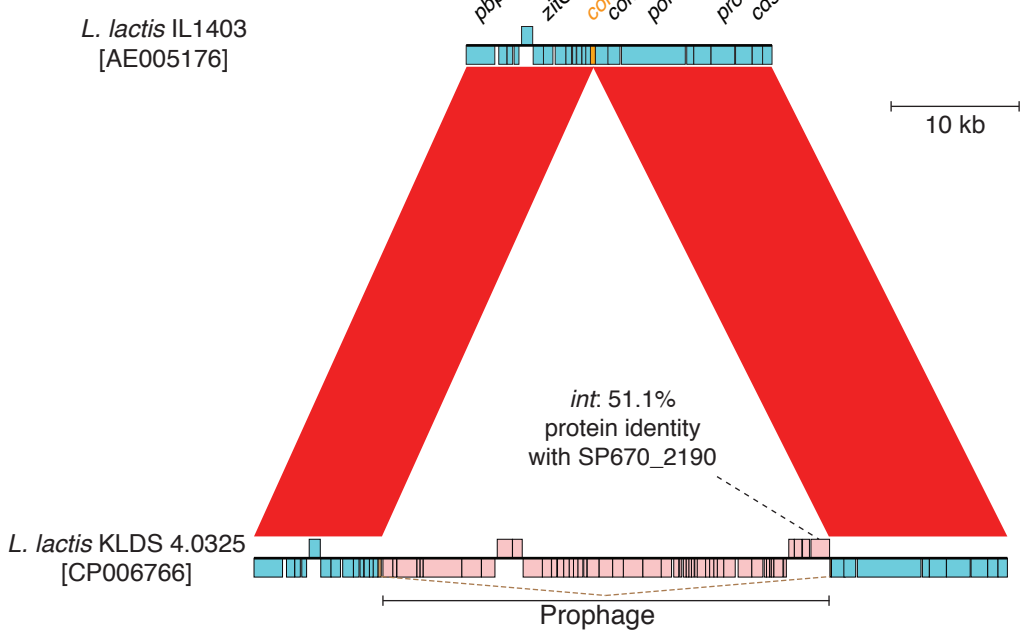

(D)

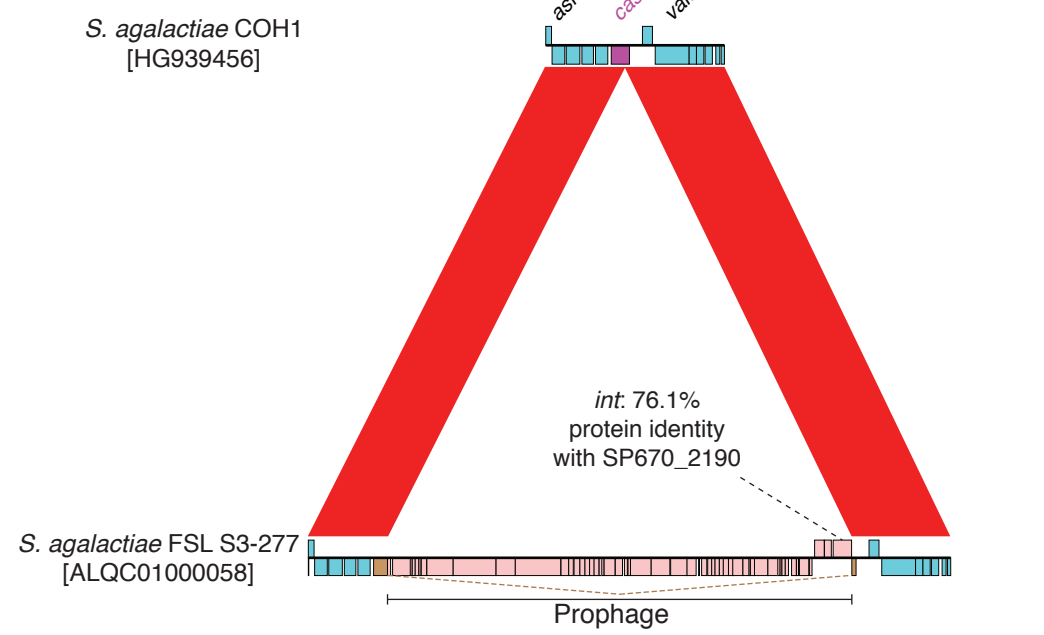

Supplement: S9 Fig — (A) Comparison of Streptococcus mutans isolates UA159 and NLML9, the latter of which has a prophage inserted into the comYC gene encoding the major structural component of the competence pilus. The accession codes of each sequence are given in brackets underneath the isolate names. Blue and orange boxes represent cellular CDSs, with the direction of transcription indicated by their vertical position relative to the horizontal line; pink boxes represent MGE CDSs in the same way. Brown boxes linked by dashed lines mark fragments of a pseudogene disrupted by an MGE insertion. The red bands link regions of similar sequence in the two loci, with the intensity of the colour representing the strength of the match. The level of protein identity between this prophage integrase and that disrupting the comYC gene of S. pneumoniae 670-6B (SP670_2190) is annotated. (B) Comparison of Streptococcus parauberis isolates KRS-02109 and KRS-02083, the latter of which has a prophage inserted into the comYC gene. (C) Comparison between Lactococcus lactis isolates IL1403 and KLDS 4.0325, the latter of which has a prophage inserted into the comYC gene. This comparison is also shown in Fig 9A. (D) Comparison between Streptococcus agalactiae isolates COH1 and FSL S3-277, the latter of which has a prophage inserted into the cas3 gene of the S. agalactiae CRISPR2 locus. This comparison is also shown in Fig 9B. (PDF) [file pbio.1002394.s010.pdf]

(A)

91

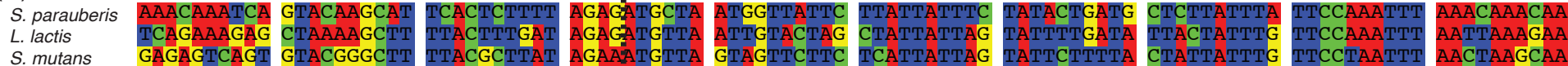

(B)

801

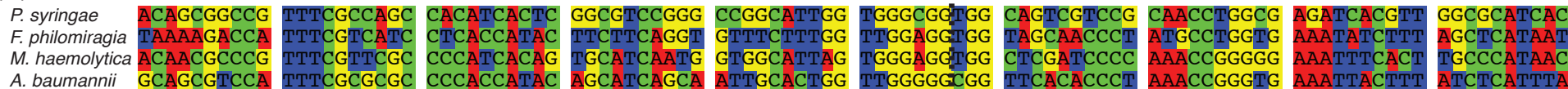

(C)

-401

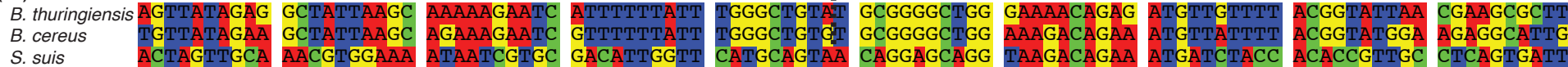

1201

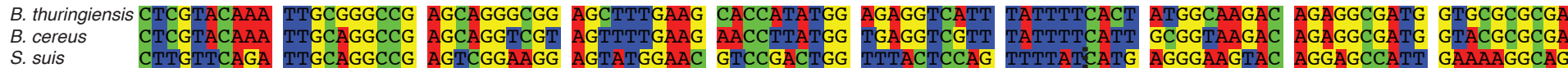

Supplement: S10 Fig — (A) Insertion of prophages into comYC. All prophages had an integrase similar to SP670_2190. This section of the comYC codon alignment shows the prophages identified in Streptococcus parauberis, Streptococcus mutans, and Lactococcus lactis all insert into an orthologous, but not perfectly conserved, location within the gene. (B) Insertion of MGEs into comM. All MGEs had an integrase similar to CF65_00446. This section of the comM codon alignment shows the MGEs identified in Pseudomonas syringae, Francisella philomiragia, Mannheimia haemolytica, and Acinetobacter baumannii all insert into an orthologous, but not perfectly conserved, location within the gene. (C) Insertion of prophages into comFA. The prophages identified in Bacillus thuringiensis and Bacillus cereus have integrases similar to LMRG_01511 (and are 80.9% identical to one another), and both insert at orthologous, but nonidentical, sites within the comFA codon alignment. However, the prophage inserted into comFA in Streptococcus suis has a distinct integrase (only 34.1% identity with that identified in B. cereus), and correspondingly inserts into a different site much further downstream in the codon alignment. (PDF) [file pbio.1002394.s011.pdf]

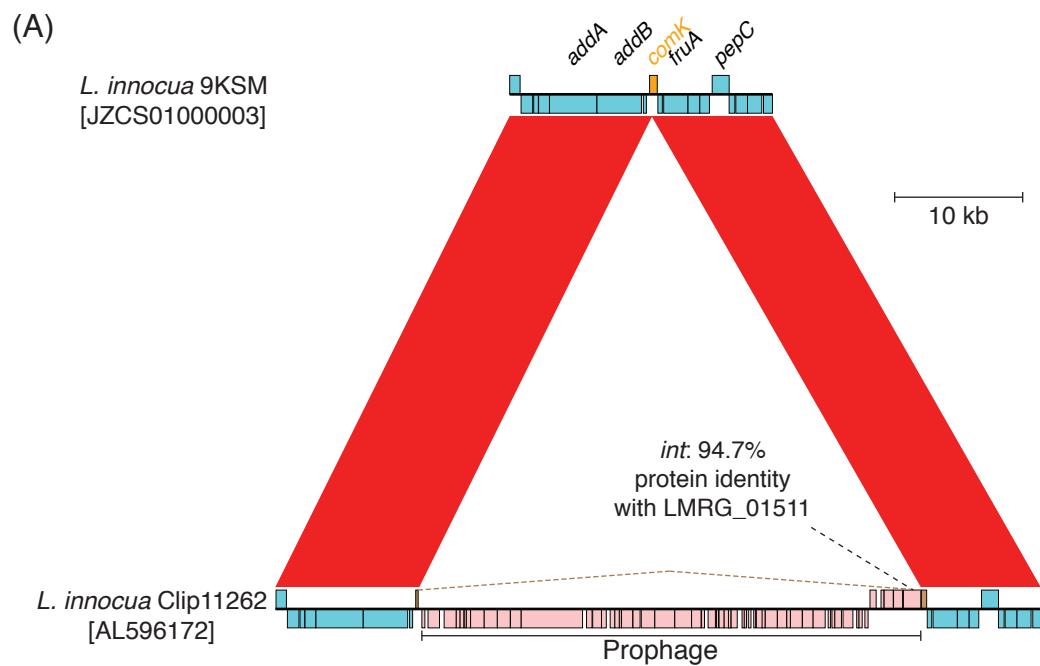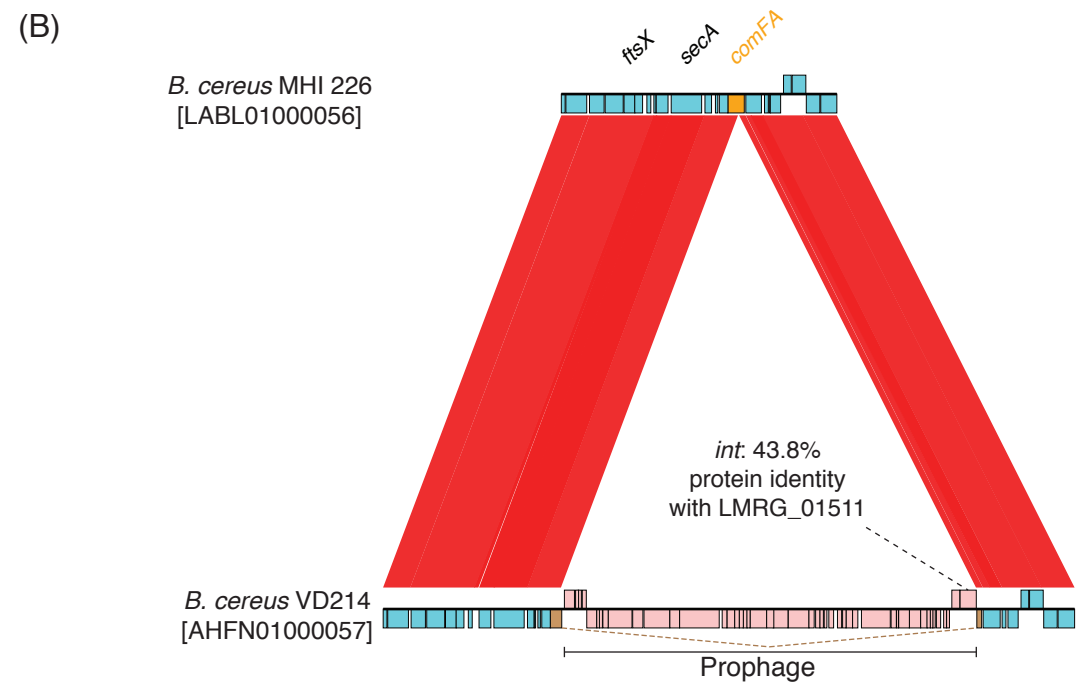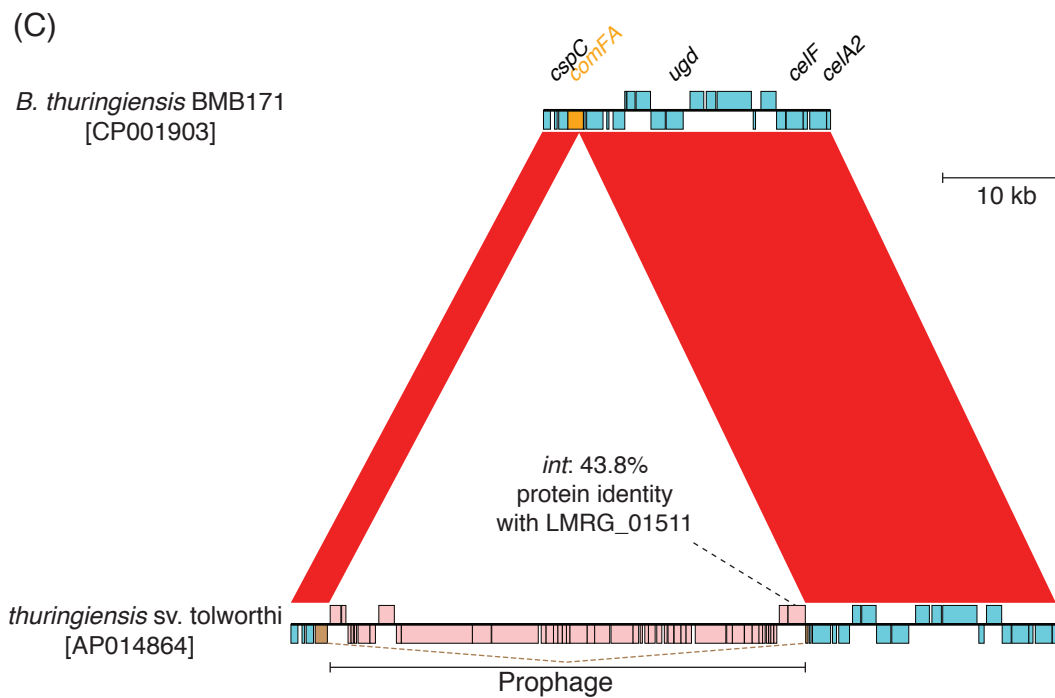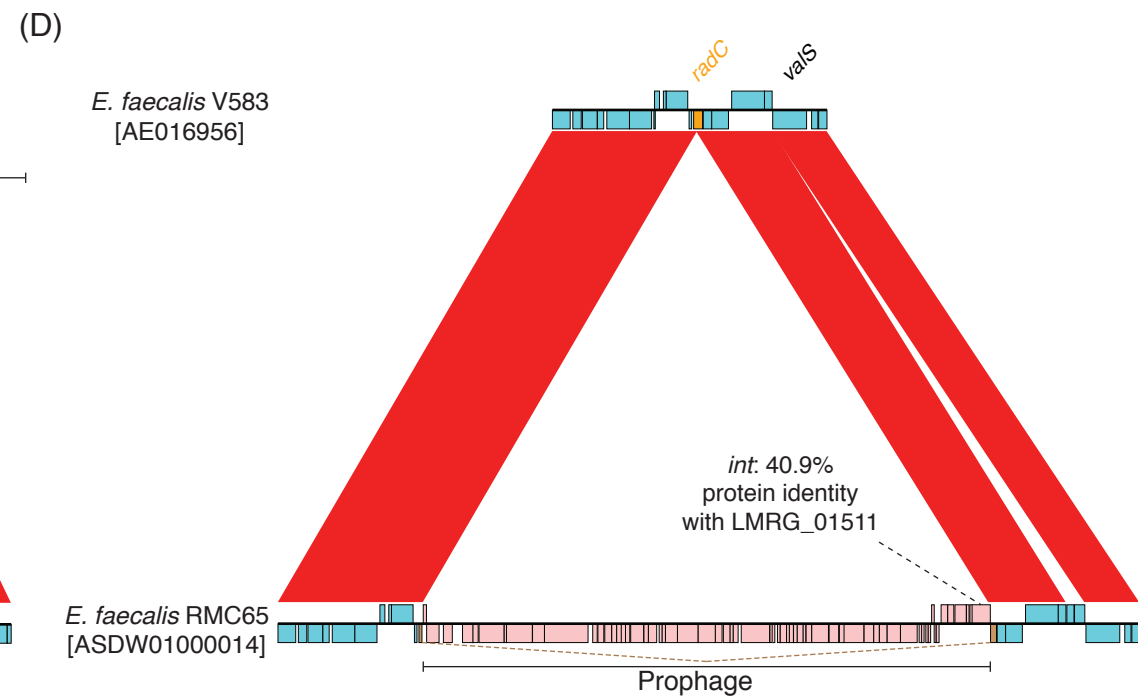

Supplement: S11 Fig — (A) Comparison of Listeria innocua isolates 9KSM and Clip11262, the latter of which has a prophage inserted into the comK gene, encoding the orthologue of the main regulator of competence in Bacillus subtilis. The comparison is displayed as described in S9 Fig. (B) Comparison of Bacillus cereus isolates MHI 226 and VD214, the latter of which has a prophage inserted into the comFA gene at a site distinct from that targeted by the prophage displayed in Fig 9C. This comparison is also shown in Fig 9D. (C) Comparison of Bacillus thuringiensis isolate BMB171 and a representative of serovar tolworthi, the latter of which has a prophage inserted into the comFA gene. (D) Comparison of Enterococcus faecalis isolates V583 and RMC65, the latter of which has a prophage inserted into the radC gene, often upregulated during competence in multiple species. (PDF) [file pbio.1002394.s012.pdf]

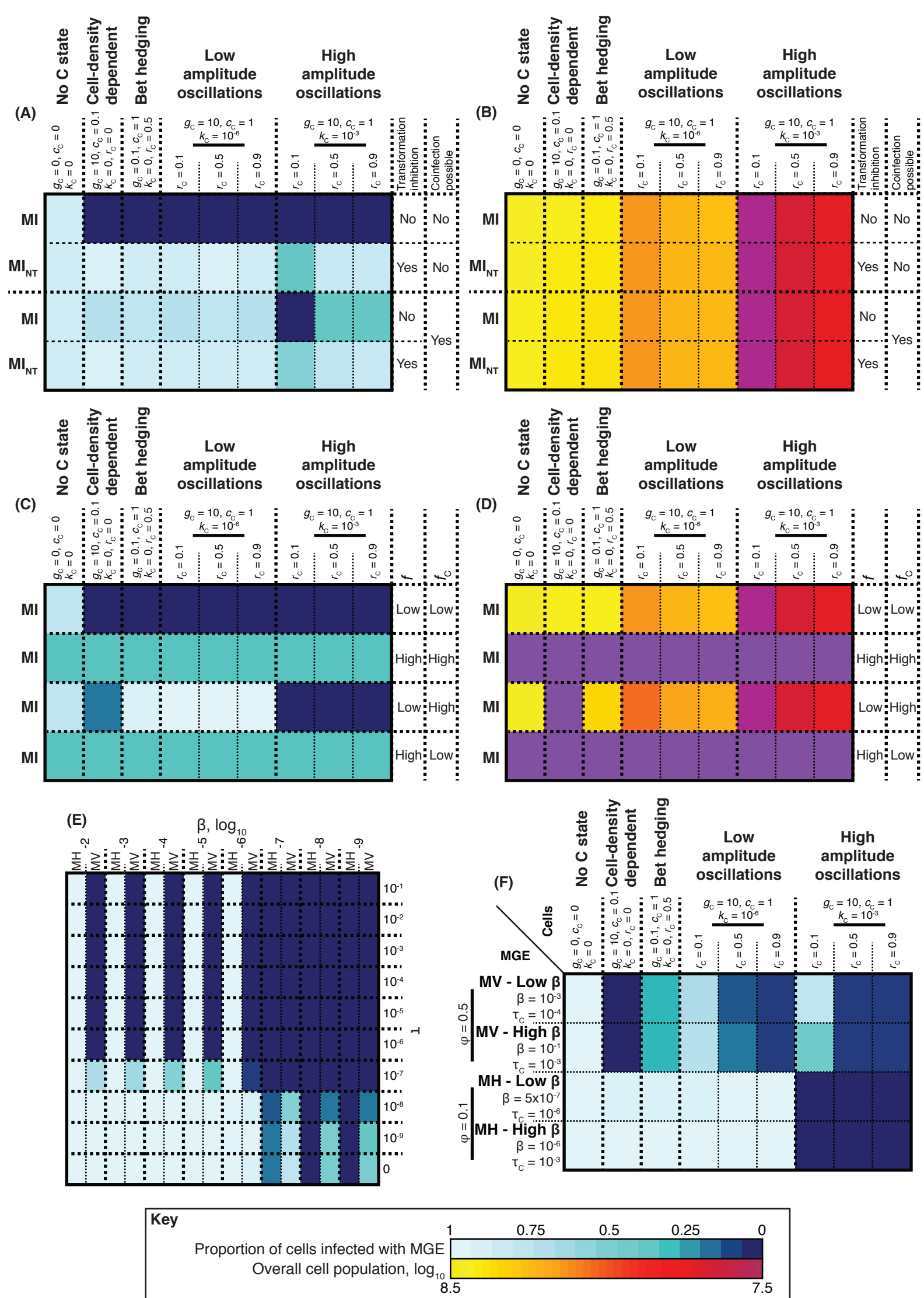

Supplement: S13 Fig — Panels A–D show further simulations investigating MGE strategies for reducing elimination by transformation. A particular issue with the simulations presented in Fig 10B and 10C was that the high value of f was especially detrimental to an MGE in early timesteps, when cells are at a low density; these simulations test for the success of different strategies when MGEs are able to invade a cell population after it had reached its carrying capacity. (A) Heatmap showing the same simulations as in Fig 10A, except that bursts of MGEs were introduced at a rate of 10−3 t -1 rather than being polymorphic in the initial population. The colours of the cells represent the proportion of the cell population infected by MGEs over the duration of the simulations. (B) Heatmap showing the overall cell population through the simulations shown in panel A on a log10 scale. (C) Heatmap showing the same simulations as in Fig 10B, except that bursts of MGEs were introduced at a rate of 10−3 t -1 rather than being polymorphic in the initial population. (D) Heatmap showing the overall cell population through the simulations shown in panel C on a log10 scale. Panels E and F evaluate the impact of artefactual antagonism between MGE infection and transformation. The model was altered such that whenever cells bound both DNA and MGEs, MGE infection occurred preferentially in place of transformation. (E) The set of simulations displayed in Fig 3B are repeated with the altered model. (F) The set of simulations displayed in Fig 4C are repeated with the altered model. Raw data are tabulated in S1 Data. (PDF) [file pbio.1002394.s014.pdf]

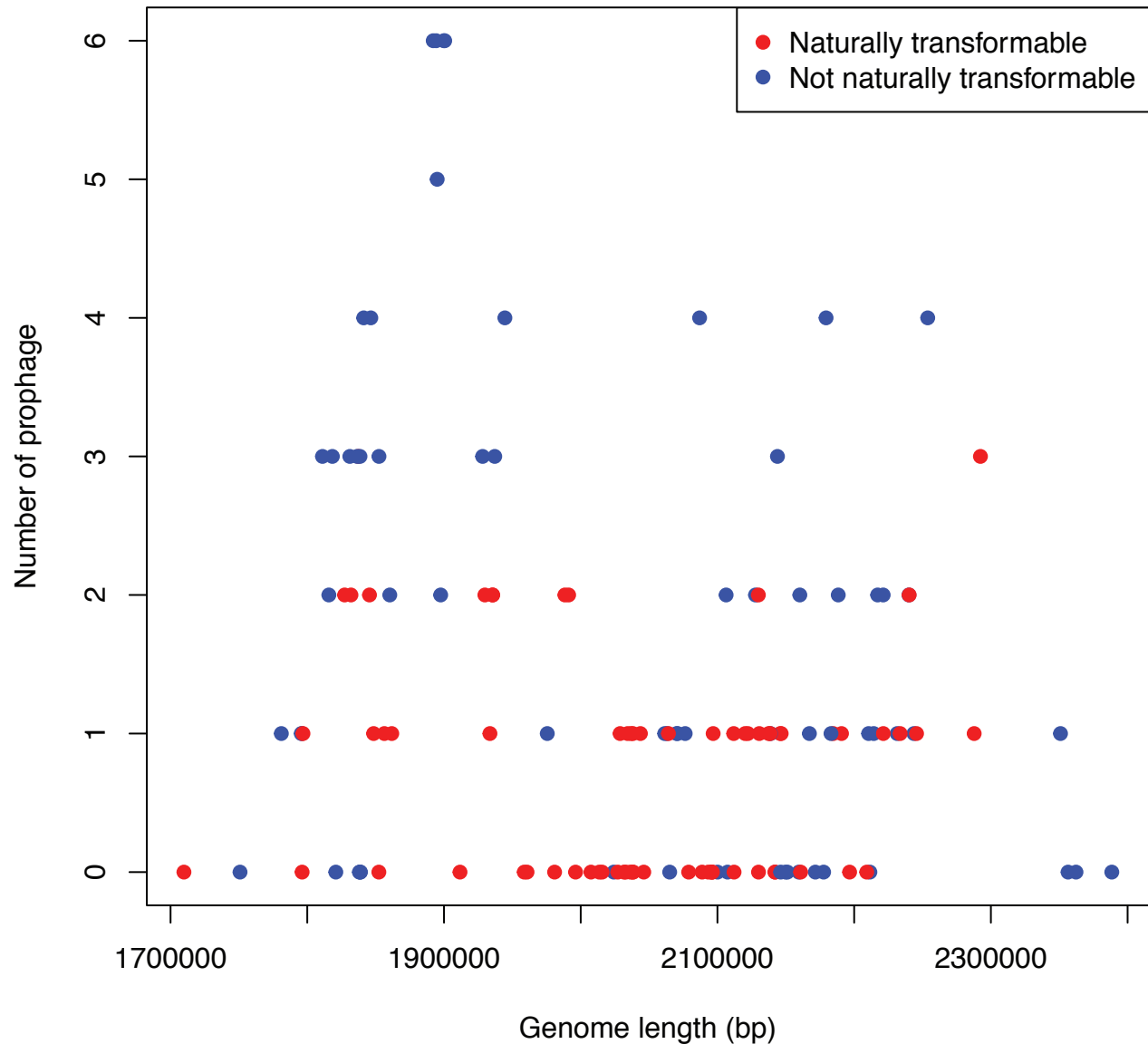

Supplement: S14 Fig — The genomes listed in S4 Table are plotted in terms of their overall size and the number of prophages detected within them. Points are coloured red if the isolate was known to be naturally transformable, or otherwise blue. (PDF) [file pbio.1002394.s015.pdf]

(A)

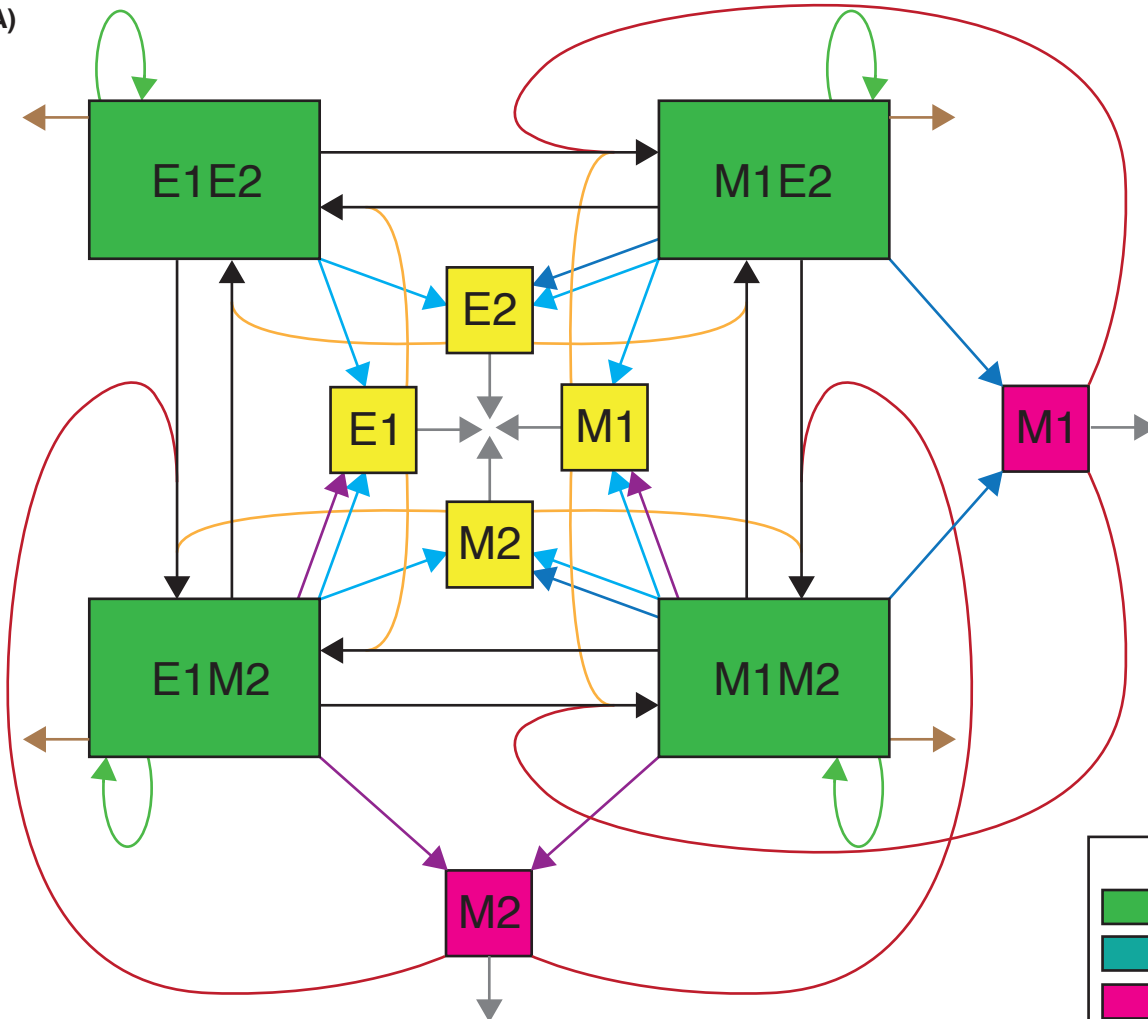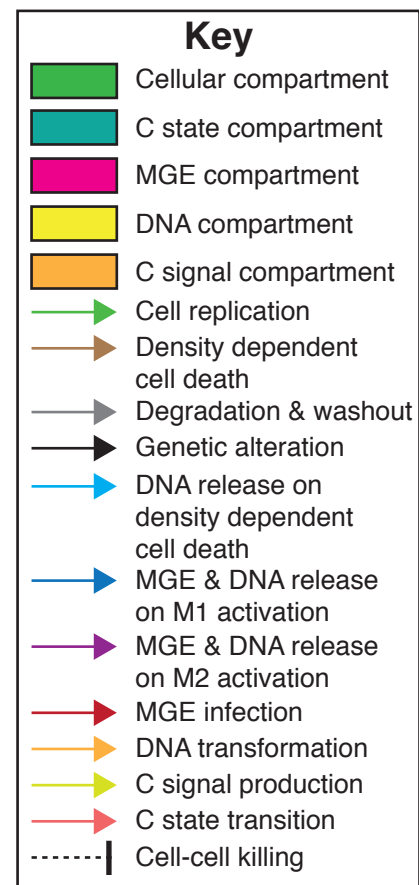

**(B)**

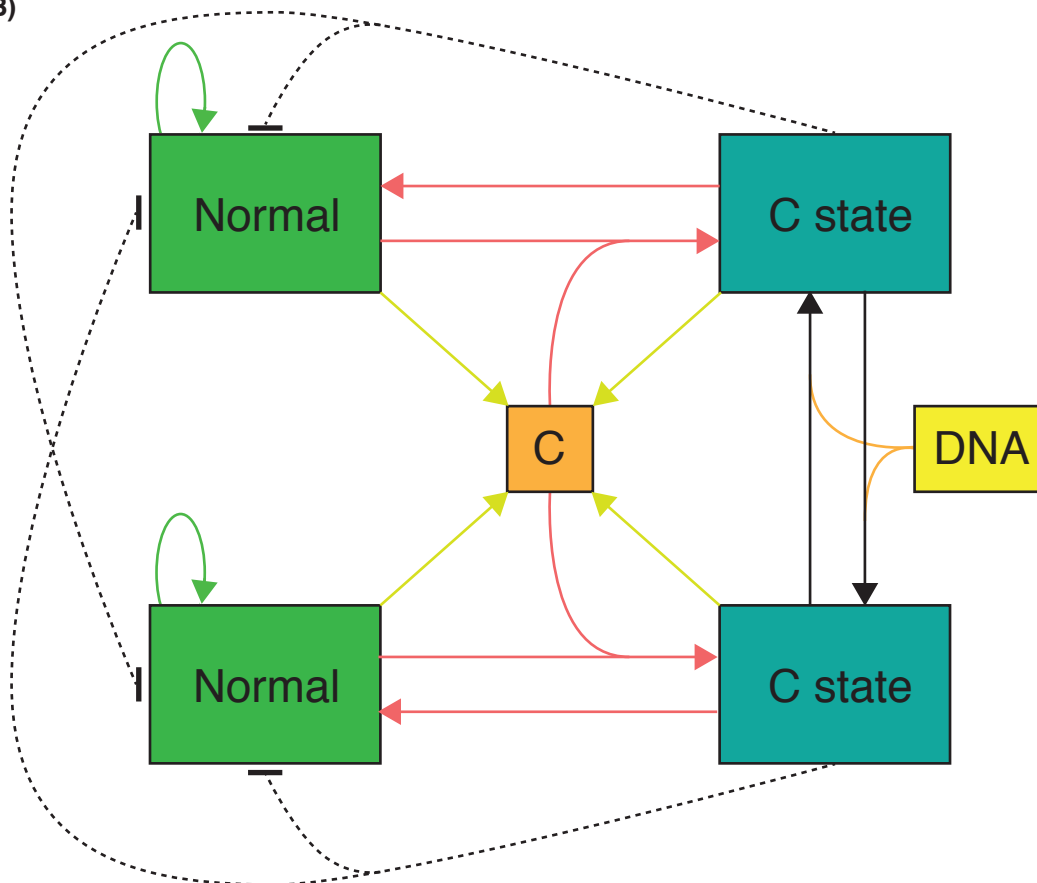

Supplement: S15 Fig — (A) Links between cellular, DNA, and MGE compartments in the basic model. Each compartment type is represented by a different colour; the cell genotype can change through either interaction with a DNA compartment (transformation) or an MGE compartment (MGE infection). Not shown are genetically “silent” transformation and infection events that deplete noncellular compartment populations but do not affect cell genotypes. Cells replicate according to their growth rate, as modified by the cost of carried MGEs, and die through density-dependent cell death and activation of some MGEs. Density-dependent cell death releases one DNA molecule of the allele present at each locus of the genotype; cell deaths associated with MGE activation release a burst of MGEs, and one molecule of the allele present at the nonactivating locus. (B) Incorporation of transient competence into the model. All cells generate a C signal, and above a threshold level, this signal drives cells to enter the C state. Cells left C state at a constant per capita rate, independent of the level of C signal. Genetic alterations through transformation were only possible when cells were in the C state. The C state also affected the population dynamics, as, in some simulations, the replication of cells was transiently arrested while they were in C state (if c C = 1 in the “bet hedging” and oscillatory growth patterns), and C-state cells also inhibited the growth of non-C-state cells through cell–cell killing (if k C > 0 in oscillatory growth patterns). (PDF) [file pbio.1002394.s016.pdf]
