# Supplementary material for: Horizontal DNA Transfer Mechanisms of Bacteria as Weapons of Intragenomic Conflict
Source: PLoS Biol. 2016 Mar 2;14(3):e1002394. doi: 10.1371/journal.pbio.1002394 (PMC4774983; doi:10.1371/journal.pbio.1002394)
Supplement: S12 Fig — (A) Comparison of Acinetobacter baumannii isolates LAC-4 and 1598530, the latter of which has an MGE inserted into a CDS encoding an orthologue of ComM, a protein identified as increasing transformation efficiency in H. influenzae. The comparison is displayed as described in S9 Fig. (B) Comparison of Mannheimia haemolytica isolates D171 and USMARC-185, the latter of which has an MGE inserted into a CDS encoding an orthologue of ComM. (C) Comparison of Francisella philomiragia isolates ATCC 25015 and FAJ, the latter of which has an MGE inserted into a CDS encoding an orthologue of ComM. (D) Comparison of Pseudomonas syringae isolates UMAF0158 and BRIP34881, the latter of which has an MGE inserted into a CDS encoding an orthologue of ComM. (PDF) [file pbio.1002394.s013.pdf]

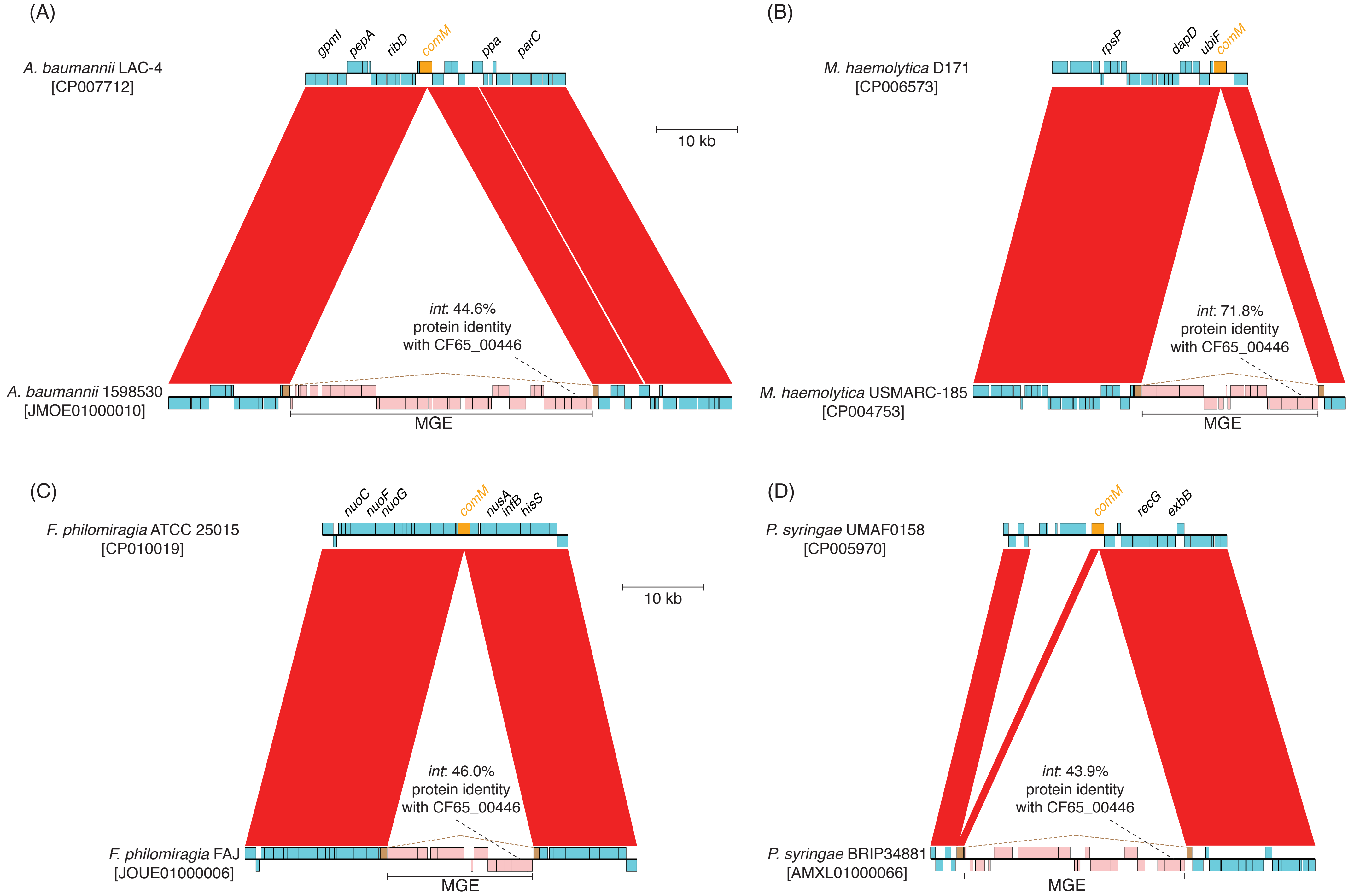

(A)
A. baumannii LAC-4
[CP007712]
gpmI
pepA
ribD
comM
ppa
parC
10 kb
int: 44.6%
protein identity
with CF65_00446
A. baumannii 1598530
[JMOE01000010]
MGE
(B)
M. haemolytica D171
[CP006573]
rpsP
dapD
ubiF
comM
int: 71.8%
protein identity
with CF65_00446
M. haemolytica USMARC-185
[CP004753]
MGE
(C)
F. philomiragia ATCC 25015
[CP010019]
nuoC
nuoF
nuoG
comM
nusA
infB
hisS
10 kb
int: 46.0%
protein identity
with CF65_00446
F. philomiragia FAJ
[JOUE01000006]
MGE
(D)
P. syringae UMAF0158
[CP005970]
comM
recG
exbB
int: 43.9%
protein identity
with CF65_00446
P. syringae BRIP34881
[AMXL01000066]
MGE
