# Supplementary material for: Horizontal DNA Transfer Mechanisms of Bacteria as Weapons of Intragenomic Conflict
Source: PLoS Biol. 2016 Mar 2;14(3):e1002394. doi: 10.1371/journal.pbio.1002394 (PMC4774983; doi:10.1371/journal.pbio.1002394)
Supplement: S1 Table — (DOCX) [file pbio.1002394.s017.docx]

| **Symbol** | **Model Parameter** | **Value unless specified** |
| --- | --- | --- |
| **Cell demography parameters** | | |
| γ | Cell growth rate | 0.2 *t*^-1^ |
| κ | Environment carrying capacity | 10^6^ |
| **C state parameters** | | |
| *c*_C_ | Growth inhibition during C state | 1 |
| *g*_C_ | Rate of entry into C state | 10 *t*^-1^ |
| *r*_C_ | Rate of exit from C state | Always specified |
| *k*_C_ | Per capita cell-cell killing during C state | Always specified |
| *e*_C_ | C signal production rate | 10 *t*^-1^ |
| *t*_C_ | Threshold C signal concentration for entering C state | 10^7^ |
| **DNA and transformation parameters** | | |
| τ | Transformation rate | 10^-4^ |
| τ_C_ | Transformation rate during C state | 10^-4^ |
| ω | Rate of washout and degradation | 0.6 |
| φ | Transformation asymmetry as a consequence of length polymorphism | 0.1 |
| **MGE parameters** | | |
| *c*_M_ | Cost of carrying MGE | Always specified |
| *f* | Frequency of activation | Always specified |
| *f*_C_ | Frequency of activation during C state | Always specified |
| β | Rate of horizontal transmission | Always specified |
| b | Mean burst size | Always specified |
