# Supplementary material for: Horizontal DNA Transfer Mechanisms of Bacteria as Weapons of Intragenomic Conflict
Source: PLoS Biol. 2016 Mar 2;14(3):e1002394. doi: 10.1371/journal.pbio.1002394 (PMC4774983; doi:10.1371/journal.pbio.1002394)
Supplement: S2 Table — The annotated prophage sequences shown in S5 and S8 Figs have been deposited in Genbank with the listed accession codes. The insertion sites of the prophages, described as in [57], are detailed along with the properties of the host bacterium. (DOCX) [file pbio.1002394.s018.docx]

| **Prophage Name** | **Insertion Site** | **Host Isolate** | **Host Isolate Lineage** | **Assembly Method** | **Accession Code** |
| --- | --- | --- | --- | --- | --- |
| ΦARI-0831 | *comYC* | 10B04734 | BC16 | Velvet | KT337368,KT337369 |
| ΦARI-0826 | *comYC* | 10B06669 | BC16 | Velvet | KT337366,KT337367 |
| ΦARI-0031 | *comYC* | 08B08535 | BC16 | Velvet | KT337340 |
| ΦARI-0004 | *comYC* | 08B01757 | BC16 | Velvet | KT337339 |
| ΦARI-0468-1 | *comYC* | 08B06220 | BC16 | Velvet | KT337355 |
| ΦARI-0462 | *comYC* | 09B05356 | BC16 | Velvet | KT337354 |
| ΦARI-0399 | SPN23F15280 - SPN23F15810 | 08B09226 | BC16 | SGA coassembly | KT337349 |
| ΦARI-0460-1 | SPN23F15280 - SPN23F15810 | 08B03577 | BC16 | SGA coassembly | KT337352 |
| ΦARI-0468-2 | SPN23F15280 - SPN23F15810 | 08B07540 | BC16 | Velvet | KT337356 |
| ΦARI-0460-2 | SPN23F15280 - SPN23F15810 | 08B02743 | BC16 | SGA coassembly | KT337353 |
| ΦARI-0131-1 | SPN23F15280 - SPN23F15810 | 09B07772 | BC16 | SGA coassembly | KT337341 |
| ΦARI-0131-2 | *purA* - SPN23F00240 | 10B01058 | BC16 | Velvet | KT337342 |
| ΦARI-0639 | SPN23F15280 - SPN23F15810 | 09B10381 | BC16 | SGA coassembly | KT337363,KT337364 |
| ΦARI-0578 | SPN23F15280 - SPN23F15810 | 09B10380 | BC16 | Velvet | KT337360 |
| ΦARI-0598 | *purA* - SPN23F00240 | 10B04316 | BC25 | SGA coassembly | KT337361,KT337362 |
| ΦARI-0274 | *comYC* | 10B00619 | BC25 | SGA coassembly | KT337343,KT337344 |
| ΦARI-0468-3 | *comYC* | 09B10874 | BC25 | SGA coassembly | KT337357,KT337359 |
| ΦARI-0285-1 | *comYC* | 10B01328 | BC25 | SGA coassembly | KT337345 |
| ΦARI-0455 | *comYC* | 09B13723 | BC25 | SGA coassembly | KT337350,KT337351 |
| ΦARI-0746 | *comYC* | 10B04751 | BC25 | Velvet | KT337365 |
| ΦARI-0923 | *comYC* | 10B00617 | BC25 | Velvet | KT337370 |
| ΦARI-0285-2 | *comYC* | 10B01328 | BC25 | SGA coassembly | KT337346 |
| ΦARI-0468-4 | *comYC* | 09B10874 | BC25 | Velvet | KT337358 |
| ΦARI-0378 | *comYC* | 08B01883 | BC25 | SGA coassembly | KT337348 |
| ΦARI-0285-3 | SPN23F15280 - SPN23F15810 | 10B01328 | BC25 | SGA coassembly | KT337347 |
| ΦARI-0995 | *purA* - SPN23F00240 | 10B06705 | BC25 | Velvet | KT337371,KT337372 |
