# Supplementary material for: Horizontal DNA Transfer Mechanisms of Bacteria as Weapons of Intragenomic Conflict
Source: PLoS Biol. 2016 Mar 2;14(3):e1002394. doi: 10.1371/journal.pbio.1002394 (PMC4774983; doi:10.1371/journal.pbio.1002394)
Supplement: S4 Table — This table displays the properties of annotated streptococcal genomes, whether the isolate is known to be naturally transformable, and the summarized output of the Phage_Finder algorithm when applied to this sequence. These data were used to test for any difference in the distribution of prophages between isolates known to be naturally transformable and those that are not. (DOCX) [file pbio.1002394.s020.docx]

| **Genome** | **Accession Code** | **Genome Length (bp)** | **Number of prophage** | **Total length of prophage (bp)** | **Naturally transformable** | **Included in Final Analysis** |
| --- | --- | --- | --- | --- | --- | --- |
| *Streptococcus agalactiae* 138P | CP007482 | 1838701 | 0 | 0 | No | Yes |
| *Streptococcus agalactiae* 138spar | CP007565 | 1838126 | 0 | 0 | No | Yes |
| *Streptococcus agalactiae* 2 22 | FO393392 | 1838867 | 0 | 0 | No | Yes |
| *Streptococcus agalactiae* 2603V/R | AE009948 | 2160267 | 2 | 73522 | No | Yes |
| *Streptococcus agalactiae* A909 | CP000114 | 2127839 | 2 | 79243 | No | Yes |
| *Streptococcus agalactiae* COH1 | HG939456 | 2065074 | 0 | 0 | No | Yes |
| *Streptococcus agalactiae* GBS1 NY | CP007570 | 2243708 | 1 | 20589 | No | Yes |
| *Streptococcus agalactiae* GBS2 NM | CP007571 | 2214297 | 1 | 39276 | No | Yes |
| *Streptococcus agalactiae* GBS6 | CP007572 | 2231475 | 1 | 39366 | No | Yes |
| *Streptococcus agalactiae* GD201008 001 | CP003810 | 2063112 | 1 | 37736 | No | Yes |
| *Streptococcus agalactiae* NEM316 | AL732656 | 2211485 | 0 | 0 | No | Yes |
| *Streptococcus agalactiae* NGBS061 | CP007631 | 2221207 | 2 | 75996 | No | Yes |
| *Streptococcus agalactiae* NGBS572 | CP007632 | 2061426 | 1 | 45911 | No | Yes |
| *Streptococcus agalactiae* SA20 06 | CP003919 | 1820886 | 0 | 0 | No | Yes |
| *Streptococcus anginosus* C1051 | CP003860 | 1911706 | 0 | 0 | Yes | Yes |
| *Streptococcus anginosus* C238 | CP003861 | 2233640 | 1 | 51008 | Yes | Yes |
| *Streptococcus anginosus* SA1 | CP007573 | 2036353 | 0 | 0 | Yes | Yes |
| *Streptococcus anginosus* subsp *whileyi* MAS624 | AP013072 | 2122284 | 1 | 24799 | Yes | Yes |
| *Streptococcus constellatus* subsp *pharyngis* C1050 | CP003859 | 1991156 | 2 | 95348 | Yes | Yes |
| *Streptococcus constellatus* subsp *pharyngis* C232 | CP003800 | 1935414 | 2 | 77119 | Yes | Yes |
| *Streptococcus constellatus* subsp pharyngis C818 | CP003840 | 1935662 | 2 | 77119 | Yes | Yes |
| *Streptococcus dysgalactiae* subsp *equisimilis* 167 | AP012976 | 2076397 | 1 | 38106 | No | Yes |
| *Streptococcus dysgalactiae* subsp *equisimilis* AC 2713 | HE858529 | 2179445 | 4 | 162791 | No | Yes |
| *Streptococcus dysgalactiae* subsp *equisimilis* ATCC 12394 | CP002215 | 2159491 | 0 | 0 | No | Yes |
| *Streptococcus dysgalactiae* subsp *equisimilis* GGS 124 | AP010935 | 2106340 | 2 | 72883 | No | Yes |
| *Streptococcus dysgalactiae* subsp *equisimilis* RE378 | AP011114 | 2151145 | 0 | 0 | No | Yes |
| *Streptococcus equi* subsp *equi* 4047 | FM204883 | 2253793 | 4 | 175325 | No | Yes |
| *Streptococcus equi* subsp *zooepidemicus* ATCC 35246 | CP002904 | 2167264 | 1 | 60707 | No | Yes |
| *Streptococcus equi* subsp *zooepidemicus* CY | CP006770 | 2107382 | 0 | 0 | No | Yes |
| *Streptococcus equi* subsp *zooepidemicus* H70 | FM204884 | 2149868 | 0 | 0 | No | Yes |
| *Streptococcus equi* subsp *zooepidemicus* MGCS10565 | CP001129 | 2024171 | 0 | 0 | No | Yes |
| *Streptococcus gallolyticus* subsp *gallolyticus* ATCC 43143 | AP012053 | 2362241 | 0 | 0 | No | Yes |
| *Streptococcus gallolyticus* subsp *gallolyticus* ATCC BAA 2069 | FR824043 | 2356444 | 0 | 0 | No | Yes |
| *Streptococcus gallolyticus* UCN34 | FN597254 | 2350911 | 1 | 47363 | No | Yes |
| *Streptococcus gordonii* str Challis substr CH1 | CP000725 | 2196662 | 0 | 0 | Yes | Yes |
| *Streptococcus infantarius* subsp *infantarius* CJ18 | CP003295 | 1988420 | 2 | 60578 | Yes | Yes |
| *Streptococcus iniae* ISET0901 | CP007586 | 2070822 | 1 | 16764 | No | Yes |
| *Streptococcus iniae* ISNO | CP007587 | 2070182 | 1 | 16764 | No | Yes |
| *Streptococcus iniae* YSFST01-82 | CP010783 | 2086959 | 4 | 83788 | No | Yes |
| *Streptococcus intermedius* B196 | CP003857 | 1996214 | 0 | 0 | Yes | Yes |
| *Streptococcus intermedius* C270 | CP003858 | 1960728 | 0 | 0 | Yes | Yes |
| *Streptococcus intermedius* JTH08 | AP010969 | 1933610 | 1 | 15935 | Yes | Yes |
| *Streptococcus lutetiensis* 033 | CP003025 | 1975547 | 1 | 40839 | No | Yes |
| *Streptococcus macedonicus* ACA DC 198 | HE613569 | 2130034 | 0 | 0 | Yes | Yes |
| *Streptococcus mitis* B6 | FN568063 | 2146611 | 1 | 67608 | Yes | Yes |
| *Streptococcus mutans* GS 5 | CP003686 | 2027088 | 0 | 0 | Yes | Yes |
| *Streptococcus mutans* LJ23 | AP012336 | 2015626 | 0 | 0 | Yes | Yes |
| *Streptococcus mutans* NN2025 | AP010655 | 2013587 | 0 | 0 | Yes | Yes |
| *Streptococcus mutans* UA159 | AE014133 | 2032925 | 0 | 0 | Yes | Yes |
| *Streptococcus mutans* UA159-FR | CP007016 | 2031692 | 0 | 0 | Yes | Yes |
| *Streptococcus oligofermentans* AS 1.3089 | CP004409 | 2142100 | 0 | 0 | Yes | Yes |
| *Streptococcus oralis* Uo5 | FR720602 | 1958690 | 0 | 0 | Yes | Yes |
| *Streptococcus parasanguinis* FW213 | CP003122 | 2171609 | 0 | 0 | No | Yes |
| *Streptococcus parauberis* KCTC 11537 | CP002471 | 2143887 | 3 | 156073 | No | Yes |
| *Streptococcus pasteurianus* ATCC 43144 | AP012054 | 2100077 | 0 | 0 | No | Yes |
| *Streptococcus pneumoniae* 670 6B | CP002176 | 2240045 | 2 | 112818 | No | Yes |
| *Streptococcus pneumoniae* 70585 | CP000918 | 2184682 | 1 | 42625 | Yes | Yes |
| *Streptococcus pneumoniae* AP200 | CP002121 | 2130580 | 1 | 47366 | Yes | Yes |
| *Streptococcus pneumoniae* ATCC 700669 | FM211187 | 2221315 | 1 | 52056 | Yes | Yes |
| *Streptococcus pneumoniae* CGSP14 | CP001033 | 2209198 | 0 | 0 | Yes | Yes |
| *Streptococcus pneumoniae* D39 | CP000410 | 2046115 | 0 | 0 | Yes | Yes |
| *Streptococcus pneumoniae* G54 | CP001015 | 2078953 | 0 | 0 | Yes | Yes |
| *Streptococcus pneumoniae* gamPNI0373 | CP001845 | 2064154 | 1 | 47799 | Yes | Yes |
| *Streptococcus pneumoniae* Hungary19A-6 | CP000936 | 2245615 | 1 | 86562 | Yes | Yes |
| *Streptococcus pneumoniae* INV104 | FQ312030 | 2142122 | 0 | 0 | Yes | Yes |
| *Streptococcus pneumoniae* INV200 | FQ312029 | 2093317 | 0 | 0 | Yes | Yes |
| *Streptococcus pneumoniae* JJA | CP000919 | 2120234 | 1 | 44592 | Yes | Yes |
| *Streptococcus pneumoniae* NT 110 58 | CP007593 | 2287774 | 1 | 52315 | Yes | Yes |
| *Streptococcus pneumoniae* OXC141 | FQ312027 | 2036867 | 1 | 45388 | Yes | Yes |
| *Streptococcus pneumoniae* P1031 | CP000920 | 2111882 | 1 | 46855 | Yes | Yes |
| *Streptococcus pneumoniae* R6 | AE007317 | 2038615 | 0 | 0 | Yes | No |
| *Streptococcus pneumoniae* 03-4156 | FQ312045 | 2024476 | 1 | 32836 | Yes | No |
| *Streptococcus pneumoniae* 03-4183 | FQ312043 | 2037254 | 1 | 45388 | Yes | No |
| *Streptococcus pneumoniae* 99-4038 | FQ312041 | 2026239 | 1 | 45388 | Yes | No |
| *Streptococcus pneumoniae* 99-4039 | FQ312044 | 2026505 | 1 | 45388 | Yes | No |
| *Streptococcus pneumoniae* A45 | HE983624 | 2129934 | 2 | 81069 | Yes | Yes |
| *Streptococcus pneumoniae* ST556 | CP003357 | 2145902 | 1 | 56073 | Yes | Yes |
| *Streptococcus pneumoniae* Taiwan19F 14 | CP000921 | 2112148 | 0 | 0 | Yes | Yes |
| *Streptococcus pneumoniae* TCH8431 | CP001993 | 2088772 | 0 | 0 | Yes | Yes |
| *Streptococcus pneumoniae* TIGR4 | AE005672 | 2160842 | 0 | 0 | Yes | Yes |
| *Streptococcus pseudopneumoniae* IS7493 | CP002925 | 2190731 | 1 | 54836 | Yes | Yes |
| *Streptococcus pyogenes* 1E1 | CP007241 | 1796152 | 1 | 42765 | No | Yes |
| *Streptococcus pyogenes* 7F7 | CP007240 | 1709790 | 0 | 0 | No | Yes |
| *Streptococcus pyogenes* A20 | CP003901 | 1837281 | 3 | 115259 | No | Yes |
| *Streptococcus pyogenes* Alab49 | CP003068 | 1827308 | 2 | 86181 | No | Yes |
| *Streptococcus pyogenes* HKU360 | CP009612 | 1944537 | 4 | 196793 | No | Yes |
| *Streptococcus pyogenes* HSC5 | CP006366 | 1818351 | 3 | 155374 | No | Yes |
| *Streptococcus pyogenes* JRS4 | AP012335 | 1811124 | 3 | 136686 | No | Yes |
| *Streptococcus pyogenes* M1 476 | AP012491 | 1831128 | 3 | 108289 | No | Yes |
| *Streptococcus pyogenes* M1 GAS | AE004092 | 1852433 | 3 | 117196 | No | Yes |
| *Streptococcus pyogenes* M23ND | CP008695 | 1846477 | 4 | 180023 | No | Yes |
| *Streptococcus pyogenes* Manfredo | AM295007 | 1841271 | 4 | 166689 | No | Yes |
| *Streptococcus pyogenes* MGAS10270 | CP000260 | 1928252 | 3 | 139783 | No | Yes |
| *Streptococcus pyogenes* MGAS10394 | CP000003 | 1899877 | 6 | 252771 | No | Yes |
| *Streptococcus pyogenes* MGAS10750 | CP000262 | 1937111 | 3 | 121236 | No | Yes |
| *Streptococcus pyogenes* MGAS15252 | CP003116 | 1750832 | 0 | 0 | No | Yes |
| *Streptococcus pyogenes* MGAS1882 | CP003121 | 1781029 | 1 | 44360 | No | Yes |
| *Streptococcus pyogenes* MGAS2096 | CP000261 | 1860355 | 2 | 101824 | No | Yes |
| *Streptococcus pyogenes* MGAS315 | AE014074 | 1900521 | 6 | 232999 | No | Yes |
| *Streptococcus pyogenes* MGAS5005 | CP000017 | 1838562 | 3 | 111077 | No | Yes |
| *Streptococcus pyogenes* MGAS6180 | CP000056 | 1897573 | 2 | 88299 | No | Yes |
| *Streptococcus pyogenes* MGAS8232 | AE009949 | 1895017 | 5 | 210407 | No | Yes |
| *Streptococcus pyogenes* MGAS9429 | CP000259 | 1836467 | 3 | 130273 | No | Yes |
| *Streptococcus pyogenes* NZ131 | CP000829 | 1815785 | 2 | 90460 | No | Yes |
| *Streptococcus pyogenes* SSI 1 | BA000034 | 1894275 | 6 | 240266 | No | Yes |
| *Streptococcus pyogenes* STAB901 | CP007024 | 1795609 | 1 | 42736 | No | Yes |
| *Streptococcus pyogenes* STAB902 | CP007041 | 1892124 | 6 | 240649 | No | Yes |
| *Streptococcus salivarius* 57.I | CP002888 | 2138805 | 1 | 27955 | Yes | Yes |
| *Streptococcus salivarius* CCHSS3 | FR873481 | 2217184 | 2 | 46965 | Yes | Yes |
| *Streptococcus salivarius* JIM8777 | FR873482 | 2210574 | 1 | 41809 | Yes | Yes |
| *Streptococcus sanguinis* SK36 | CP000387 | 2388435 | 0 | 0 | Yes | Yes |
| *Streptococcus* sp I G2 | CP006805 | 1992567 | 0 | 0 | No | No |
| *Streptococcus* sp I P16 | CP006776 | 2023580 | 0 | 0 | No | No |
| *Streptococcus* sp VT 162 | CP007628 | 2045418 | 0 | 0 | No | No |
| *Streptococcus suis* 05HAS68 | CP002007 | 2188363 | 2 | 32485 | Yes | Yes |
| *Streptococcus suis* 05ZYH33 | CP000407 | 2096309 | 0 | 0 | Yes | Yes |
| *Streptococcus suis* 6407 | CP008921 | 2292360 | 3 | 115270 | Yes | Yes |
| *Streptococcus suis* 98HAH33 | CP000408 | 2095698 | 0 | 0 | Yes | Yes |
| *Streptococcus suis* A7 | CP002570 | 2038409 | 0 | 0 | Yes | Yes |
| *Streptococcus suis* BM407 | FM252032 | 2146229 | 0 | 0 | Yes | Yes |
| *Streptococcus suis* D12 | CP002644 | 2183059 | 1 | 40166 | Yes | Yes |
| *Streptococcus suis* D9 | CP002641 | 2177656 | 0 | 0 | Yes | Yes |
| *Streptococcus suis* GZ1 | CP000837 | 2038034 | 0 | 0 | Yes | Yes |
| *Streptococcus suis* JS14 | CP002465 | 2137435 | 1 | 38021 | Yes | Yes |
| *Streptococcus suis* P1 7 | AM946016 | 2007491 | 0 | 0 | Yes | Yes |
| *Streptococcus suis* S735 | CP003736 | 1980887 | 0 | 0 | Yes | Yes |
| *Streptococcus suis* SC070731 | CP003922 | 2138568 | 1 | 41742 | Yes | Yes |
| *Streptococcus suis* SC84 | FM252031 | 2095898 | 0 | 0 | Yes | Yes |
| *Streptococcus suis* SS12 | CP002640 | 2096866 | 1 | 36529 | Yes | Yes |
| *Streptococcus suis* ST1 | CP002651 | 2034321 | 1 | 36963 | Yes | Yes |
| *Streptococcus suis* ST3 | CP002633 | 2028815 | 1 | 15252 | Yes | Yes |
| *Streptococcus suis* T15 | CP006246 | 2240234 | 2 | 82433 | Yes | Yes |
| *Streptococcus suis* TL13 | CP003993 | 2038146 | 1 | 20168 | Yes | Yes |
| *Streptococcus suis* YB51 | CP006645 | 2043655 | 1 | 15252 | Yes | Yes |
| *Streptococcus thermophilus* ASCC 1275 | CP006819 | 1845495 | 2 | 29522 | Yes | Yes |
| *Streptococcus thermophilus* CNRZ1066 | CP000024 | 1796226 | 0 | 0 | Yes | Yes |
| *Streptococcus thermophilus* JIM 8232 | FR875178 | 1929905 | 2 | 30348 | Yes | Yes |
| *Streptococcus thermophilus* LMD 9 | CP000419 | 1856368 | 1 | 23872 | Yes | Yes |
| *Streptococcus thermophilus* LMG 18311 | CP000023 | 1796846 | 1 | 44172 | Yes | Yes |
| *Streptococcus thermophilus* MN ZLW 002 | CP003499 | 1848520 | 1 | 17062 | Yes | Yes |
| *Streptococcus thermophilus* ND03 | CP002340 | 1831949 | 2 | 29901 | Yes | Yes |
| *Streptococcus thermophilus* SMQ-301 | CP011217 | 1861792 | 1 | 48053 | Yes | Yes |
| *Streptococcus uberis* 0140J | AM946015 | 1852352 | 0 | 0 | No | Yes |
